# A Systematic Review of Research on the Meaning, Ethics and Practices of Authorship across Scholarly Disciplines

**DOI:** 10.1371/journal.pone.0023477

**Published:** 2011-09-08

**Authors:** Ana Marušić, Lana Bošnjak, Ana Jerončić

**Affiliations:** 1 Department of Research in Biomedicine and Health, University of Split School of Medicine, Split, Croatia; 2 Office for Science and Department of Research in Biomedicine and Health, University of Split School of Medicine, Split, Croatia; Cochrane Acute Respiratory Infections Group, Italy

## Abstract

**Background:**

The purpose of this systematic review was to evaluate evidence about authorship issues and provide synthesis of research on authorship across all research fields.

**Methods:**

We searched bibliographical databases to identify articles describing empirical quantitive or qualitative research from all scholarly fields on different aspects of authorship. Search was limited to original articles and reviews.

**Results:**

The final sample consisted of 123 articles reporting results from 118 studies. Most studies came for biomedical and health research fields and social sciences. Study design was usually a survey (53%) or descriptive study (27%); only 2 studies used randomized design. We identified four 4 general themes common to all research disciplines: authorship perceptions, definitions and practices, defining order of authors on the byline, ethical and unethical authorship practices, and authorship issues related to student/non-research personnel-supervisor collaboration. For 14 survey studies, a meta-analysis showed a pooled weighted average of 29% (95% CI 24% to 35%) researchers reporting their own or others' experience with misuse of authorship. Authorship misuse was reported more often by researcher outside of the USA and UK: 55% (95% CI 45% to 64%) for 4 studies in France, South Africa, India and Bangladesh vs. 23% (95% CI 18% to 28%) in USA/UK or international journal settings.

**Interpretation:**

High prevalence of authorship problems may have severe impact on the integrity of the research process, just as more serious forms of research misconduct. There is a need for more methodologically rigorous studies to understand the allocation of publication credit across research disciplines.

## Introduction

Recently, PubMed – the largest bibliographical database in biomedicine made a new record in the number of authors on the byline of an indexed article: 2080 authors needed 165 lines on the PubMed site to spell out their surnames and initials. The paper was from high energy physics [1] and the number of authors probably did not surprise any physicist. It also probably did not surprise those involved in clinical trials, where the number of authors can also reach thousands [2]. But researchers in many areas of social sciences and humanities may expect to be sole authors, or perhaps discuss the senior authorship between a supervisor and a doctoral student [3].

Regardless of the practices in the number of authors, authorship and publication credit is the currency system of research and academic community, with both positive and negative implications [4]. To improve the practices of responsible authorship, it is important to understand the definition(s) of authorship, its impact on research productivity and roles of different stakeholders in the allocation of publication credit. The purpose of this systematic review was to evaluate evidence about authorship issues and provide a synthesis of research on authorship across research fields.

## Methods

### Selection Criteria

All articles describing empirical quantitive or qualitative research from all scholarly fields on the definition of or criteria for authorship, authors' contribution to the research and manuscript, order of authors on the byline, opinions of researchers and/or editors on different aspects of authorship were selected for the review. We excluded articles describing research that used journal articles and their authors for analyzing collaborative or citation networks; authorship in the context of citation analysis; analysis of research collaboration outputs of institutions, groups, research fields; trends in authorship in journals, groups of journals, fields, institutions, countries, geographical regions; gender of authors in journals, groups of journals, fields, institutions, countries, geographical regions. Articles describing research on authorship attribution in literature, taxonomy, and psychology/cognitive research were also excluded. Articles that did not provide methodological and/or numerical information (such as found in letters and conference proceedings) were also excluded.

### Database Search and Retrieval of Articles

Electronic databases were searched on 17 January 2011 using a general text search term ‘authorship’ to increase the sensitivity of the search. Where possible, the search was limited to original research articles and reviews. The search included all databases available from the on-line source of the Croatian Academic Network (CARNet): Databases included Agricola (1970 to 2011 Week 3); Business Source Complete (since 1886); CINAHL (since 1981); Current Contents (1993 Week 27 to 2011 Week 3); EBM reviews (2005 to 2011 Week 3), including Cochrane Database of Systematic Reviews, ACP Journal Club, DARE, CCTR, CMR, HTA, and NHSEED; ERIC (1965 to 2011 Week3); GeoRef (since 1966); Food Science and Technology Abstracts (1969 to 2011 Week 3); INSPEC (1969 to 2011 Week 3); Library, Information Science & Technology (since mid-1960ties); MEDLINE (1950 to 2011 Week 3); PsycINFO (1967 to 2011 Week 3); SCOPUS (1960 to 17 Jan 2011); and Web of Knowledge (1991 to 17 Jan 2011), including Science Citation Index Expanded (SCI-EXPANDED), Social Sciences Citation Index (SSCI) and Arts & Humanities Citation Index (A&HCI). There were no language restrictions. There was no attempt to search grey literature because our study was focused on authorship research in the mainstream science. Hand search of relevant journals was not performed because authorship topics are published in a variety of journals and because we used a sensitive rather than specific search; only the theme issues of JAMA, related to peer review conferences were searched by hand.

The titles and available abstracts of retrieved records were examined for possible inclusion in the review. Selected full text articles were used as a starting point for the berrypicking search, a technique which included footnote, citation and author searching [5], as well as searching of ‘Related citations’ feature in MEDLINE, where appropriate. Our own work and knowledge of the literature, as well as other experts in the field, were also used to find possible articles for inclusion.

Titles and abstracts of all retrieved articles were screened by one author to determine if they met inclusion criteria, and the selection was verified by the other author. Disagreements were discussed and full text articles were retrieved in cases of doubt for review and decision on inclusion. Full texts of the articles were reviewed by both authors; disagreements were resolved by discussion. A description of the population and extractable data were the minimum for the inclusion in the systematic review.

### Analysis and presentation of findings

We used a data collection form (Table S1) to extract study type, intervention, setting, participant demographics, and outcome measures. Study quality was assessed on the basis of study design, sample size and sampling frame, response rate, and outcome measures. Disagreements in the assessment and data extraction were resolved by discussion and consensus. As most of the included studies were observational studies with heterogeneous measurements, we could not perform a statistical pooling of the results. Instead, we performed a qualitative synthesis of the results, providing a narrative description of the results. We also identified themes arising from the study results and assigned the studies to these defined categories.

For the percentage (proportion) of respondents who recalled their own problems or problems of colleagues with authorship issues (n = 14 studies), we were able to perform quantitative data synthesis. The data were transformed with Freeman-Tukey variant of the arcsine square root [6]. Pooled effect size was calculated as the back-transform of weighted mean of the transformed proportions, using DerSimonian-Laird weights for random effects model [6]. Homogeneity was tested with Cochran's Q test based upon inverse variance weights [7]. Differences between groups of studies were tested with Mann-Whitney U test using inverse variance weighted averages. Publication bias was assessed with funnel plot Harbord bias indicator [6]. The statistical analyses were run on an SPSS software package 17 for Windows (SPSS Inc., Chicago, IL, USA), using the ‘MeanES’, ‘MetaF’ and ‘MetaReg’ macros by David B. Wilson [7].

## Results

**Figure 1:**
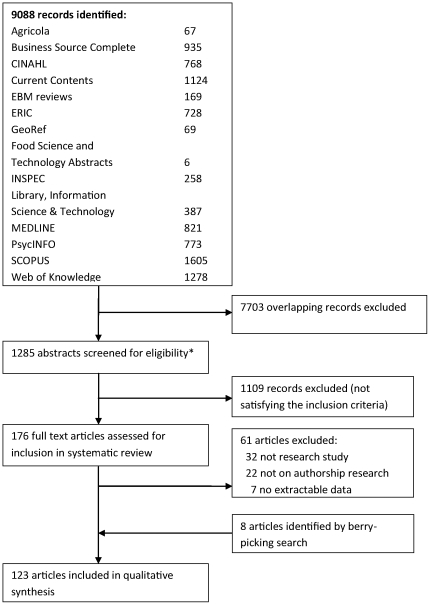
Selection of the articles for the systematic review. Search keyword was ‘authorship’, limited to article as a publication type, search performed 15 January 2010. Asterisk : inclusion criteria – quantitive or qualitative research on the definition of or criteria for authorship, authors' contribution to the research and manuscript, order of authors on the byline, opinions of researchers and/or editors on authorship criteria, opinions of researchers and/or editors on authorship order; exclusion criteria: 1. research topics which use journal articles and their authors as a starting point for studying: collaborative or citation networks; authorship in the context of citation analysis; analysis of research collaboration outputs of institutions, groups, research fields; trends in authorship in journals, groups of journals, fields, institutions, countries, geographical regions; gender of authors in journals, groups of journals, fields, institutions, countries, geographical regions; 2. analysis of authorship attribution in literature, taxonomy, and psychology/cognitive research.

8988 references were retrieved from the bibliographic database search (Figure 1). After excluding 7703 overlapping records, 1285 abstracts were screened for eligibility. After excluding 1109 records, 176 full text articles were assessed for the inclusion in systematic review. Out of these, 61 articles were excluded on the basis of full-text assessment because they did not present research results (n = 32), did not address authorship as defined in the inclusion criteria (n = 22) or had no extractable data (n = 7). The berrypicking search of full articles yielded 8 articles, and no additional relevant articles were identified by experts in the field. Thus the total number of included articles with original data was 123 [8]–[130], presenting 118 studies (list of articles in Table S2). All articles were published in English except 1 in Spanish, 1 in Portuguese and 1 in Dutch.

Most of the articles were published in health sciences (n = 66), including 52 studies from general medicine and/or biomedicine (1 study was presented in 2 articles [38], [52]), 6 from nursing, and 7 from more than one research field. There were 33 articles from social sciences, including 12 studies from psychology, 12 from economics/business/marketing, 3 from social work, 2 from education research, 1 from information research and 3 from more than one research field. Out of 9 articles from natural sciences, 3 were from physics (results from 1 study presented in 2 articles [79], [101]), 3 from chemistry (1 study presented in 3 articles [119], [126], [127]) and 1 each in agriculture and ecology. There were 15 articles covering more than one scientific area, where 2 articles presented results from 1 study [8], [9]. No studies on authorship in humanities could be identified.

Most of the studies were performed in international science journals (n = 47) or in the USA (46 studies reported in 49 articles). Five studies were performed in Canada, 4 in Australia, 2 in South Africa, 2 in the Netherlands and 1 (2 articles) in the international physics laboratory in Europe (CERN). A study was performed in each of the following countries: Bangladesh, Brazil, Croatia, France, India, Iran, Pakistan, Spain, Sweden and UK. Finally, 1 study had respondents from both the US and Canada, and for 1 study it was not clear whether it was performed in the UK, US or both countries.

The design of most studies was cross-sectional survey (63 studies published in 65 articles), with response rates ranging from 16% to 100%. There were 32 descriptive studies (published in 34 articles), mainly literature analysis. One involved mathematical modeling [43], 1 was a test-retest study [94] and 1 combined a survey and intervention design [93]. Five studies were qualitative (1 published in 2 articles) [34], [79], [101], [104], [116], [128] and 2 randomized [86], [102]; there were 3 before-and-after studies [90], [106], [121] and 1 cohort study [92].

Many studies (n = 85) had methodological limitations. Out of 65 studies involving survey designs, 27 did not report details on survey development or testing. All before-and-after studies had no controls. Out of 6 articles on qualitative studies, 5 did not report on the protocol and details of the sample or data analysis procedure or independent confirmation of identified themes and their analysis. Randomized studies involved questionnaires and were single blinded; 1 described piloting of the questionnaire. Quality assessment of the articles (Table S2) revealed that most studies had clearly stated objectives, but the description of the sample and sampling procedures sometimes lacked detail. Study findings were stated with varying levels of detail and in some reports it was difficult to discern the findings of qualitative and quantitive analyses.

**Figure 2:**
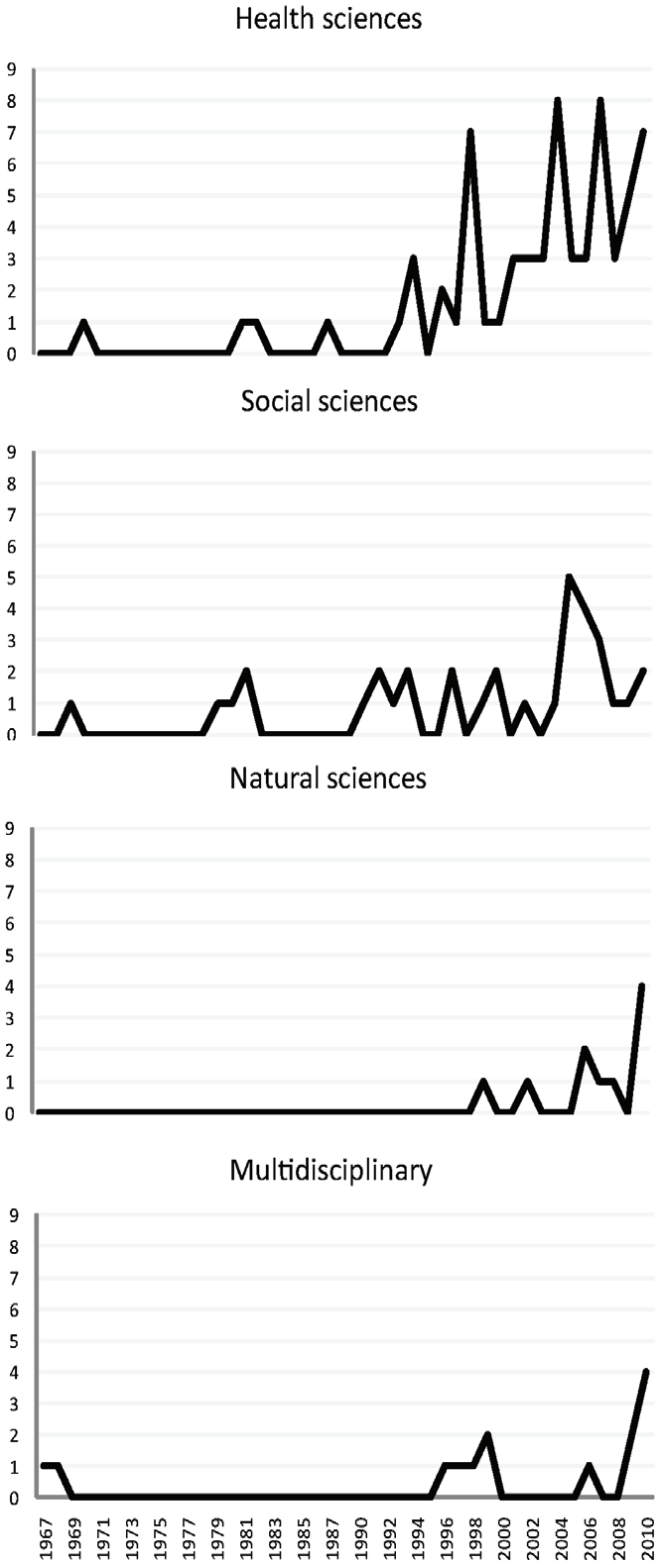
Trends in publications on authorship research in different research areas since 1967, when the first research report was identified [**8**]. No studies were identified in humanities.

The first identified study addressed the differences in name ordering of Nobel laureates from different disciplines in comparison to their colleagues in 1967 [8], [9], followed in 1970 by a study on name ordering in physiology journal [10] and a seminal survey of publication credit assignment practices in psychology [11]. In the 80ties, there were only 7 studies across all disciplines, whereas the 90ties witnessed the increasing trend in authorship research, particularly in health sciences (Figure 2).

We identified 4 general themes studied across research disciplines: authorship perceptions, definitions and practices (n = 58 articles), defining order of authors on the byline (n = 45), ethical and unethical authorship practices (n = 46), and authorship issues related to student/non-research personnel-supervisor collaboration (n = 19). Most of the articles explored one of these themes (n = 90), 21 explored 2, 11 explored 3 and 1 article addressed all 4 themes.

### Authorship definitions, perceptions and practices

Fifty-four studies examined the perceptions of authorship buy different stakeholders, authorship definitions in use and actual practices, and contributions for deserving authorship (Table 1 and Table S3): 31 studies from the health research field [13], [16], [23], [25], [26], [31], [35], [36], [39], [41], [47], [50], [52], [54], [57], [60], [65], [66], [77], [80], [82], [94], [100], [102]–[104], [106], [110]–[112], [121];12 studies from social sciences [11], [12], [14], [18], [24], [27], [33], [34], [48], [49], [55], [91], 6 studies from more than one research field [29], [45], [90], [116], [122], [128] and 5 studies from natural sciences, published in 6 articles [46], [58], [79], [101], [119], [126].

**Table 1:** Definitions of authorship, contributions for deserved authorship and authorship practices*.

Article	Study population	Study topic
Spiegel, 1970 [11]	Psychologists in USA	Single contribution that qualifies for authorship; Preferred solution to multiple authorship
Bridgewater,^a^ 1981 [12]	Academic psychologists in USA	Agreement of respondents on qualifying contributions for authorship
Werley,^a^ 1981 [13]	Nursing professionals in USA	Single contribution that qualifies for authorship; Preferred solution to multiple authorship
von Glinow, 1982 [14]	Professionals associated with management journals in USA	Opinion of editors vs. editorial review board on collection of data as deserving authorship contribution
Waltz,^a^ 1985 [16]	Health professionals in nursing in USA	Contributions that do not deserve authorship
van der Kloot, 1991 [18]	Social psychologists and psychometricians in The Netherlands	Scores on a continuum scale of deserving authorship for different contributions
Diguisto, 1994 [23]	University research staff in Australia	Value of contributions for deserving authorship
Floyd, 1994 [24]	Authors of articles published in management journals	Importance of contributions for authorship
Goodman, 1994 [25]	First authors or research articles in general medical journal	Prevalence of authors who satisfied ICMJE authorship criteria
Shapiro, 1994 [26]	First authors from USA of research articles in general medical journal	Most frequent contributions by all authors as reported by first author
Wagner, 1994 [27]	Single, first or second author in a psychology journal	Contribution importance for authorship
Eastwood, 1996 [29]	Postdoctoral fellows at a university	Sufficient contribution for authorship
Bhopal, 1997 [31]	Staff from university medical school in UK	Reported agreement with ICMJE authorship criteria; Contributions that alone merit authorship
Hamilton, 1997 [33]	Business and non-business university faculty in USA	Deserving joint authorship for a single contribution
Netting, 1997 [34]	University faculty and student in focus groups in USA	Emerging themes in authorship
Almeida, 1998 [35]	Mental health professionals (physicians and non-physicians) in Brazil	Opinions of physicians vs. non-physicians on contributions valid for granting authorship
Butler, 1998 [36]	Nurses expected to publish research in Canada	Agreement among nurses of different professional status on different authorship scenarios
Hoen, 1998 [39]	Authors of articles published in national general medical journal in The Netherlands	Awareness and fulfilment of ICMJE criteria
White, 1998 [41]	First authors of papers on nursing research from USA	Knowledge of authorship guidelines; Reported contributions to different aspects of manuscript; Prevalence of articles with all authors qualifying for authorship
Rose, 1999 [45]	Ethics statements from scientific professional organizations in USA	Prevalence of statements on authorship in ethics codes
Tarnow, 1999 [46]	Postdoctoral fellows in physics in USA	Knowledge of association authorship guidelines; Discussion of authorship criteria with supervisor; Criteria for designating postdocs or others as authors
Yank, 1999 [47]	Articles in general medical journal	Contributions declared for authors and persons in acknowledgment lists
Bartle, 2000 [48]	Faculty and students from psychology departments in USA	Most important contributions for authorship; Opinion of students vs. faculty on APA ethical guidelines
Hart, 2000 [49]	Co-authors of papers in library science	Importance of research tasks for authorship
Price, 2000 [50]	Faculty from institutions granting graduate degrees in nursing in USA	Criteria most important for authorship; Opinion on number of criteria needed for authorship; Role of journals in authorship issues
Phillips,^b^ 2001 [52]	Authors of articles in large and small medical journals	Acknowledgement of medical writing assistance as authorship
Altman, 2002 [54]	Authors of articles in general medical journals	Recognition of a methodologist as an author
Laband, 2002 [55]	Authors in economic and agricultural economics journals	Fraction of production team given authorship rights in economics vs. agricultural economics
Mowatt, 2002 [57]	Corresponding authors of Cochrane systematic reviews	Contributions of authors vs. Cochrane editorial team
Tarnow, 2002 [58]	Members of American Physical Society (APS)	Use of APS authorship guidelines; Preference of authorship guidelines
Foote, 2003 [60]	Biomedical journals	No. journals without definition of authorship in guidelines
Cohen, 2004 [65]	Members of US and Canadian Academy of Pathology (USCAP)	Use of authorship guidelines; Expressed preference of authorship guideline
Etemadi, 2004 [66]	Editors of medical journals in Iran	Opinions on criteria for authorship
Pignatelli, 2005 [77]	Senior clinical researchers in France	Practices in authorship; Agreement with ICMJE criteria
Birnholtz, 2006 [79]	Researchers in high energy physics	Themes in authorship in high energy physics
Burbonniere, 2006 [80]	Researchers at a clinical centre in Canada	Satisfaction with use of in-house authorship guideline
Dhaliwal, 2006 [82]	Faculty in teaching hospital in India	Acceptable criteria for authorship
Funk, 2007 [90]	NIH postdoctoral fellows in USA	Awareness and use of authorship guidelines after RCR training
Geelhoed, 2007 [91]	Authors of research articles in clinical psychology journals	Most common opinions on authorship decision process
Ilakovac, 2007 [94]	Authors of research articles in general medical journal	Reliability of contribution declaration form for corresponding author
Wager, 2007 [100]	Guidelines for authors in medical journals	Presence of authorship guidance; Reference to ICMJE authorship criteria
Birnholtz,^c^ 2008 [101]	Researchers in high energy physics	Emerging themes in authorship
Ivaniš, 2008 [102]	Authors of research articles in general medical journal	Prevalence of authors satisfying ICMJE criteria when declaring contributions in a binary vs. ordinal rating scale
Lang, 2008 [103]	Experienced medical writers from USA	Opinion on deserved authorship for medical writers
Louis, 2008 [104]	High profile researchers in biomedicine in USA	Identified guiding factors for authorship decisions
Baerloccher, 2009 [106]	Original research articles in general medical journals	Number of authors after introduction of contribution disclosure requirement
Pulido, 2009 [110]	Spanish authors in health who publish in international journals	Most important contributions for any author vs. first author; Knowledge of ICMJE criteria
Rowan-Legg, 2009 [111]	Guidelines published in biomedical journals	Prevalence of journals with authorship addressed in guidelines
Samad, 2009 [112]	Pakistani medical and dental journals	Prevalence of journals with no guidance on authorship
Castleden, 2010 [116]	Researchers involved in research with Indigenous communities in Canada	Collective/community authorship as emerging practice
House,^d^ 2010 [119]	Faculty from departments of chemistry in USA	Factors explaining deserved authorship; Factors explain and influences on authorship
McDonald, 2010 [121]	Articles from medical journals	Influence of authorship restriction policies on number of authors, 1986 to 2006
Morris, 2010 [122]	All (n = 39) Australian universities	No. universities with authorship policy and policy rating
Seeman,^d^ 2010 [126]	Faculty from departments of chemistry in USA	Situational differences in authorship decisions
Street, 2010 [128]	Staff and doctoral candidates in health research at Australian universities	Emerging themes in authorship

*Abbreviations: ICMJE, International Committee of Medical Journal Editors; APA, American Psychological Association; NIH, National Institutes of Health, USA; RCR, responsible conduct of research.

a Partial or full replication or modification of questionnaire by Spiegel and Keith Spiegel, 1970 [11].

b Sub-analysis of data from Flanagin et al [38].

c The same study as Birnholtz, 2006 [79].

d House and Seeman [119] and Seeman and House [126] present results from the same study.

Conception of research/research design and writing the manuscript were identified as most qualifying contributions for authorship across different sciences, geographical regions and the time span from 1970ties to present [12], [16], [18], [23], [24], [26], [27], [35], [47]–[50], [66], [82], [110]. Deserving authorship was not restricted or granted to researchers but to other member of the research team who made important contribution [13], [14], [16], [36], [41], [55], [126]. Recently, collective or community authorship has emerged in different disciplines involved in research with Indigenous communities [116]. In health research, the position of medical writers and statisticians/methodologists has been explored in more detail. Most professional medical writers would expect authorship when they contributed to the collection and/or analysis of data and contribute to the manuscript writing [103] but authorship as acknowledgment for medical writing assistance was reported by 16% or authors [52]. Methodologists were recognized as authors in 65% to 88% articles in general medical journals [54], and editorial teams of Cochrane review groups for systematic review/meta-analyses made important contributions to published articles [57].

Five surveys asked for a single contribution that would qualify for authorship: the most frequent choice for psychologists was choice of statistical method and data analysis (55%) [11], manuscript drafting for nursing professionals (53%) [13], design of the study for postdoctoral fellows from different disciplines (92%) [29], providing statistical advice on an ongoing basis for researchers at a medical school (92%) [31] and data interpretation or doing 20–50% of the work for business/non-business faculty (90%) [33]. In the latter study, more business than non-business faculty would grant authorship for only final preparation and submission of a manuscript (44% vs. 21%).

Several studies explored if stakeholders in research provided authorship guidance. A 1999 study of the professional organizations in the USA showed that up to 56%of them had non-specific statements but that only 17% had clear criteria for authorship [45]. A recent study from Australia demonstrated that, even when there are national authorship policies, the universities do not fully comply with them [122]. Biomedical journals, which generally declare to follow the authorship criteria of the International Committee of Medical Journal Editors (ICMJE) [131], often do not explicitly state these criteria in their guidelines for authors or have outdated versions [60], [100], [111], [112]. It is thus not surprising that just over 60% of authors in health research journals satisfy authorship criteria [24], [39], [41] and that many authors and editors are not familiar with such criteria or think they are not realistic or fair [31], [39], [41], [65], [66], [77]. Similar lack of knowledge or use of guidelines was demonstrated for postdoctoral fellows or active researchers in physics in the USA [46], [58] and faculty and students in psychology [48]. A study of postdoctoral fellows at the National Institutes of Health in the USA in 2007 showed that training in responsible conduct of research did not significantly change the awareness and use of authorship guidelines [90]. For faculty in departments of chemistry in the USA, the factors that explained the variance in influences on authorship decisions was graduate school education (31%), institutional or other sources (19%) and personal values (14%) [119]. Experience from a medical setting in Canada indicated that researchers may be satisfied with guidelines developed in-house [80], whereas a study of authors from clinical psychology journals demonstrated that the satisfaction with both the process and outcomes of authorship decisions significantly increases with the use of guidelines [91]. Authors from clinical psychology journals identified the first authors as the most common deciders on co-authorship, and indicated factors other than effort and contributions which affected authorship decisions: taking project leadership, loyalty or obligation, power issues, and publish or perish pressures, with tenured faculty giving significantly less value to these factors, being more satisfied with the process and perceiving themselves to have more power relative to others [91]. One study described the influence of a specific sub-field, number of publications, county of PhD degree, and previous experiences with authorship in providing credit research contributions on the academic chemistry environment in the USA [126].

Although psychologists used to declare their contributions in published articles already in the 1970ties [11], contribution declaration was implemented by many medical journals only 20 years later [132]. This policy did not show any effect on the number of authors [106], [121] and a test-retest study demonstrated that the reliability of contribution declaration forms used in journals is too low to warrant their use in making conclusions on authorship [94]. A randomized study in a medical journals demonstrated that using ordinal rating scale instead of binary ‘yes-no’ declaration of contributions significantly increased the number of authors satisfying the ICMJE authorship criteria [102].

Four studies, published in 5 articles, qualitatively explored authorship issues [34], [79], [101], [104], [128]. Although most of them had methodological limitations, they identified emerging themes on authorship in social sciences, high energy physics, biomedicine, and multidisciplinary teams in health research. All studies identified common social factors in authorship decisions, best summarized in the study of Louis et al from 2008 [104], which identified fairness, reciprocity and sponsorship as main guiding factors in making authorship decision by high-profile researchers in biomedicine. For high energy physics, where collaborations increase to thousand co-authors [1], the individual still remains the unit of the research effort but larger collaborations increases the range of contributions and includes both infrastructure and discovery efforts [79], [101]. In such situation, it is particularly difficult for a young researcher to balance the practice of attributing credit to a large group with their individual need for recognition and promotion, so they have to develop pragmatic strategies for professional survival.

### Authorship order

The order of authors on the byline was specifically addressed by 46 studies (Table 2 and Table S4): 22 studies from the health research field [10], [13], [16], [17], [22], [26], [28], [30], [36], [37], [41], [47], [51], [57], [59], [63], [65], [77], [89], [95], [115], [129], 18 studies from social sciences [11], [14], [15], [21], [27], [49], [55], [60], [72], [74], [81], [83], [85], [87], [96], [105], [108], [117], 5 studies from more than one research field [8], [9], [43], [84], [118] and 1 study from natural sciences [58].

**Table 2:** Order of authors on the byline*.

Article	Study population	Study topic
Zuckerman, 1967 [8]	Nobel laureates in USA and matched scientists	1^st^ authorship of laureates vs. others
Zuckerman,^a^ 1968 [9]	Nobel laureates in USA and matched scientists	Ratio observed/expected frequency of papers with 6 or more authors and name order pattern for laureates vs. others
Over, 1970 [10]	Articles published in *J Physiol* 1961–1964	Percent authors with A–E vs. P–Z surnames in a journal with alphabetical author listing
Spiegel, 1970 [11]	Psychologists in USA	Preferred method for authorship order when contributions are equal
Werley,^b^ 1981 [13]	Nursing professionals in USA	Preferred method for authorship order when contributions are equal
von Glinow, 1982 [14]	Professionals associated with management journals in USA	Preferred method for ordering authors
Over, 1982 [15]	Articles in psychology journals	Change in number of articles with alphabetical ordering of authors from 1949 to 1979
Waltz,^b^ 1985 [16]	Health professionals in nursing in USA	Preferred method for authorship order when contributions are equal
Gay,^b^ 1987 [17]	Educators in nursing USA	Methods for determining authorship
McCarl, 1993 [21]	Citations in 5 journals on agricultural economics	Chance of having a citation when first-author has a Z or A surname
Shulkin, 1993 [22]	Articles by chairs of department of medicine in USA	Last-authorship papers of short-term vs. long-term chairs
Shapiro, 1994 [26]	First authors from USA of research articles in general medical journal	No. and type of contributions of first vs. last author
Wagner, 1994 [27]	Single, first or second author in a psychology journal	Mean percent contributions for different authorship positions
Davies, 1996 [28]	Chairs of pediatric departments and deans of medical faculties in Canada	Opinions on value of first author contribution in individual or group authorship
Slone, 1996 [30]	First authors from USA on papers from a radiology journal	Reported contributions of first authors vs. 5^th^–10^th^ author
Butler, 1998 [36]	Nurses in Canada, expected to publish research	Agreement among nurses that order of authorship should be based on contributions, not status
Drenth, 1998 [37]	Authors of articles in general medical journal 1975–1995	Prevalence of senior level authors as last authors in 1975 vs. 1995
White, 1998 [41]	First authors from USA on papers on nursing research	Knowledge of agency or institution guidelines for authorship sequencing
Engers, 1999 [43]	Articles from journals on law, economics, social sciences, natural sciences or medicine	Prevalence of alphabetical ordering of authors
Yank, 1999 [47]	Articles in general medical journal	Contributions for different authorship byline position
Hart, 2000 [49]	Co-authors of papers in library science	Most prevalent method of ordering authors
Chambers, 2001 [51]	Articles in general medical journal	Most common letters for surnames of first authorship
Laband, 2002 [55]	Authors of articles in economic and agricultural economics journals	Prevalence of alphabetized co-authorship
Mowatt, 2002 [57]	Corresponding authors of Cochrane systematic reviews	Reported practices in deciding on authors' order
Tarnow, 2002 [58]	Members of American Physical Society (APS)	Probability of change after initial authorship list is determined
Bhandari, 2003 [59]	Editorial board members of medical journal in USA	Agreement on method for authorship order
Bhandari, 2004 [63]	Chairs of surgery or medicine departments in Canada	Change in assignment of authorship credit to first or last author when they are corresponding authors
Cohen, 2004 [65]	Members of US and Canadian Academy of Pathology (USCAP)	Probability of change after initial authorship list is determined
Meyer, 2004 [68]	Editorial members of accounting journals and young accounting faculty members in USA	Perceived behaviour appropriateness and occurrence and actual knowledge of occurrence of co-authorship issues
Apgar, 2005 [72]	Members of Society for Social Work and Research in USA	Opinions on authorship order
Hilmer, 2005 [74]	Faculty members of agricultural economics departments in USA and their publications	Prevalence of alphabetical authorship in co-authored vs. multi-authored articles; Estimated annual salary return to an additional article depending on alphabetical authorship
Pignatelli, 2005 [77]	Senior clinical researchers in France	Practice of ordering authorship
Brown, 2006 [81]	Multiauthored articles from academic institutions published n marketing journals	Percent alphabetical ordering of authors
Einaw, 2006 [83]	Faculty of economic or psychology departments, Econometric Society (ES) fellows, Nobel laureates and Clark Winners, authors of articles in economics journals in USA	Increase in probability for tenure status with each letter closer to the front of the alphabet; Percent multiauthored articles with alphabetical authorship in economics journals
Laband, 2006 [84]	Articles in journals from medicine, natural sciences, economics, social sciences and general journals	Mean change in prevalence of alphabetical authorship in co-authored articles from 1974 to 1999
Manton, 2006 [85]	Business faculty in USA	Opinion on method of listing authors
Moore, 2006 [87]	Authors of articles in educational research journals	Preferred method of authorship order
Baerlocher, 2007 [89]	Articles in general medical journals	Satisfaction of ICMJE criteria 1 and 2, depending on byline position
Kurichi, 2007 [95]	Chairs of surgery departments in USA medical schools	Likelihood for authorship position in regard to serving as chair
Manton, 2007 [96]	Faculty of colleges of business in USA	Preferred method of listing co-authors
van Praag, 2008 [105]	Articles published in mainstream economics journals	Prevalence of articles with alphabetical authorship
Hu, 2009 [107]	Articles in biomedical or multidisciplinary journals	Increase in prevalence of equal first authorships
Maciejeovsky, 2009 [108]	Faculty members and advanced graduate students from economics, marketing and psychology in USA/UK	Prevalence of alphabetical authorship; Preferences for credit to a position in multiauthored papers; Inferences based on authorship order
Akhabue, 2010 [115]	Original research articles from general medical journal	Trends in equal authorships from 2000 to 2009
Chan, 2010 [117]	Multi-authored original research articles from academic real estate journals	Prevalence of alphabetical authorship from 1990 to 2006; Likelihood for alphabetical authorship
Frandsen, 2010 [118]	Articles from economics, library information science (LIS) and high-energy physics (HEP) journals	Yearly change in share of articles with alphabetic authorship from 1978 to 2007
Walker, 2010 [129]	Corresponding authors of original research articles in medical journals	Opinion on authorship position with greatest merit for promotion; Practice of ordering authorship position

*Abbreviations: ICMJE, International Committee of Medical Journal Editors.

a The same study as Zuckerman, 1967 [8].

b Partial or full replication or modification of questionnaire by Spiegel and Keith Spiegel, 1970 [11].

For researchers in most sciences, the amount of work and not prestige or position were the preferred method for determining authorship order [10], [11], [13], [15]–[17], [36], [49], [51], [57], [59], [72], [77], [85], [87], [96], [108]. Notable exceptions were the fields of management research [14] and most areas of economy [21], [43], [55], [74], [81], [83], [84], [105], [108], [117], [118], where alphabetical ordering of authors has been the norm for a long time. Economists calculated that with each letter closer to the front of the alphabet there was an increase in the probability to be tenured at top economy departments and receive professional recognition [83], as well as a significant increase of 0.41% in estimated salary return for an additional article with alphabetical authorship [74] and a 3.3% chance that 1% lower ranked alphabet letter would increase total and annual publication output in mainstream economics journals [105]. In real estate journals, likelihood for alphabetical authorship was greater in higher quality articles or higher academic ranking of authors or with authors from Europe [117]. Greater academic ranking or prestige, such as Nobel prize, was associated with more generosity in giving prominent place to collaborators or accepting alphabetical authorship [8], [9], [83]. Nobel laureates had more first authorship at 20 years of age but less when they were 40, compared to scientists matched in discipline, age, type of affiliation, and initial letter of the surname [8]. Alphabetical authorship seems to be a constant feature of economics journals and perhaps and emerging one for social sciences journals, with a mean increase in prevalence of 9.9% and 18.6%, respectively, from 1974 to 1999, compared to a sharp decrease of 47.8% in general journals such as *Science* and *Nature*, 82% in medical journals, and 39.1% in natural science journals in the same period [84]. A recent study analyzing changes from 1978 to 2007 confirmed that alphabetical authorship was stable in economics and common for authors in high energy physics, but decreasing for articles in library information research [118].

Several studies explored the importance of the author's position on the byline, particularly in the field of biomedical research. Most prestige and greatest contribution was expected from the first author [26], [28], [30], [47], [59], [63], [89], [129], whereas seniority brought prestige with the last author position [22], [26], [37], [47], [59], [63], [95]. In medicine and multidisciplinary journals, there is a recent trend of equal authorship of the first 2 or more authors [107], [115].

Most of the researchers psychology, nursing and social work favored pre-study agreement as the best policy for ordering names on the byline [11], [13], [16], [72]. In medicine, this was reported as a common practice [129]. Only 5% of first authors from the USA on nursing research papers reported that they were aware of any agency or institution guidelines for authorship sequencing [41]. In physics, the probability of change after initial authorship list was determined was 4% for decrease and 12% for increase [58], similar to pathology researchers in medicine (3% and 18%, respectively) [65].

### Ethics of authorship

Ethical and unethical practices in authorship and perceptions about them were analyzed in 51 studies (Table 3 Table S5): 34 studies from the health research field [13], [16], [17], [30], [31], [36], [38], [41], [42], [50], [53], [56], [57], [61], [62], [64], [65], [67], [69], [70], [73], [75], [77], [82], [86], [92], [93], [97], [99], [109], [114], [120], [123], [125], 10 studies from social sciences [11], [14], [18], [33], [68], [71], [76], [85], [91], [96], 3 studies from natural sciences [46], [58], [127] and 4 studies from more than one research field [29], [90], [107], [113].

**Table 3:** Ethical and unethical authorship practices*.

Article	Study population	Study topic
Spiegel, 1970 [11]	Psychologists in USA	Ethical practices in granting authorship
Werley,^a^ 1981 [13]	Nursing professionals in USA	Ethical practices in granting authorship
von Glinow,1982 [14]	Professionals associated with management journals in USA	Ethical practices in granting authorship
Waltz,a 1985 [16]	Health professionals in nursing in USA	Ethical practices in granting authorship
Gay,^a^ 1987 [17]	Health professionals in nursing in USA	Ethical practices in granting authorship and publishing multiple publications from the same study
van der Kloot, 1991 [18]	Social psychologists and psychometricians in The Netherlands	Agreement about authorship between professors and junior researchers
Eastwood, 1996 [29]	Postdoctoral fellows at a university in USA	Willingness to engage in giving undeserved authorship
Slone, 1996 [30]	First authors from USA on papers from a radiology journal	Reported undeserved authorship for co-authors; Reasons for undeserved authorship; Time of decision on authorship
Bhopal, 1997 [31]	Staff from university medical school in UK	Reported problems with authorship; Gift authorship
Hamilton, 1997 [33]	Business and non-business university faculty in USA	Views on unethical authorship practices
Bulter, 1998 [36]	Nurses expected to publish research in Canada	Agreement among nurses about ethical issues in authorship
Flanagin, 1998 [38]	Corresponding authors from USA on articles in large and small medical journals	Reported prevalence of research articles with undeserved or undisclosed or ghost authorship
White, 1998 [41]	First authors from USA on papers on nursing research	Reported issues, problems and concerns about author inclusion or ordering
Wilcox, 1998 [42]	Cases brought to university ombuds office in USA	Authorship issues in cases 1991/92 vs. 1996/97
Tarnow, 1999 [46]	Postdoctoral fellows in physics in USA	Reported papers where supervisor did not satisfy APS guidelines; Reasons for inappropriate authorship
Price, 2000 [50]	Faculty from institutions granting graduate degrees in nursing in USA	Experiences and opinions on unethical authorship practices
Reidpath, 2001 [53]	Authors of articles published in general medical journal	Reported authorship was among stipulations for sharing data-set from their article
Mainous, 2002 [56]	Corresponding authors of research articles in medical journals	Personal or professional concerns in authorship; Opinion on effective ways for authorship decisions
Mowatt, 2002 [57]	Corresponding authors of Cochrane systematic reviews	Prevalence of honorary authors or ghost and honorary authors
Tarnow, 2002 [58]	Members of American Physical Society (APS)	Probability that an additional author is inappropriate; Comfort for younger vs. older respondent to deny undeserving authorship
Hwang, 2003 [61]	Research articles in medical journal	Prevalence of undeserved ICMJE authorship
Bates, 2004 [62]	Research articles in medical journals with different contribution declaration forms	Prevalence of undeserved ICMJE authorship
Buchkowsky, 2004 [64]	Clinical trials published in medical journals	Increase in author affiliation with industry from 1981/1984 to 1997/2000
Cohen, 2004 [65]	Members of US and Canadian Academy of Pathology (USCAP)	Probability that an additional author is inappropriate; Reported denying undeserved authorship
Marušić, 2004 [67]	Research articles in general medical journal	Prevalence of undeserved ICMJE authorship
Meyer, 2004 [68]	Editorial members of accounting journals and young accounting faculty members in USA	Perceived behaviour appropriateness/behaviour occurrence/actual knowledge of occurrence of co-authorship issues
Procyshyn, 2004 [69]	Research articles on antipsychotic drugs in medical journals	Prevalence of authors affiliated with 3 pharmaceutical firms
Szirony, 2004 [70]	Nursing faculty members in USA	Formal teaching to graduate students about authorship credit in publications; Ethical decisions in authorship
Apgar, 2005 [71]	Members of Society for Social Work and Research in USA	Unethical granting of authorship
Freda, 2005 [73]	Editors of nursing journals	Reported prevalence of ethical issues about authorship encountered in editorial work
Joubert, 2005 [75]	Authors of research papers from university in South Africa	Reported prevalence of ethical issues in authorship
Mixon Jr, 2005 [76]	Articles published in more and less prestigious economics journals	Ratio between number of authors and contributors in acknowledgment
Pignatelli, 2005 [77]	Senior clinical researchers in France	Opinions and reported experience on gift and ghost authorship
Dhaliwal, 2006 [82]	Faculty in teaching hospital in India	Reported conflict over authorship
Manton, 2006 [85]	Business faculty in USA	Reported experience of unethical granting of authorship
Marušić, 2006 [86]	Authors of articles in general medical journal	Prevalence of authors not satisfying ICMJE criteria in different forms of contribution declaration
Funk, 2007 [90]	NIH postdoctoral fellows in USA	Ethically appropriate responses to case vignettes at 3 time points after training on RCR
Geelhoed, 2007 [91]	Authors of articles in clinical psychology journals	Experiences about fairness and ease of authorship decision process
Gotsche, 2007 [92]	Clinical trial protocols and publications from Sweden	Prevalence of ghost authorship
Hren, 2007 [93]	Medical students with or without instruction on ICMJE criteria, physicians and medical faculty in Croatia	Opinions on eligible contributions for authorship
Manton, 2007 [96]	Faculty of colleges of business in USA	Reported that co-authors did very little/no work
Peppercorn, 2007 [97]	Articles on breast cancer clinical trials in medical journals	Prevalence of pharmaceutical company authorship on published studies
Tungaraza, 2007 [99]	Published clinical trials on psychiatric drug treatment	Prevalence of industry-authored studies
O'Brien, 2009 [109]	Corresponding authors of original research articles in general medical journals	Reported experience or opinion unethical authorship
Wager, 2009 [113]	Editors of journals published by Blackwell	Reported experience of ethical issues in authorship
Ahmed, 2010 [114]	Participants in bioethics course in Bangladesh	Experiences of authorship conflicts
Lacasse, 2010 [120]	Public policies of academic medical centres in USA	Prevalence of policies banning ghostwriting
Nastasee, 2010 [123]	Articles in medical journals	Increase in acknowledgment of medical writing from 2000 to 2007
Rose, 2010 [125]	Clinical trials published in oncology journal	Odds for authors reporting financial ties to industry:
Seeman,^b^ 2010 [127]	Faculty from departments of chemistry in USA	Experience of unethical behaviour in authorship

*bbreviations: NIH, National Institutes of Health, USA; RCR, responsible conduct of research.

a Partial or full replication or modification of questionnaire by Spiegel and Keith Spiegel, 1970 [11].

b The same study as House and Seeman [119] and Seeman and House [126].

In 4 studies that used variations of the same survey questionnaire [11], researchers in psychology and nursing showed agreement in their opinion on ethical authorship decisions: not giving authorship to a colleague who failed to keep agreement on study work and multiple publications from the same study, provided that there is indication that they are part of the same study [11], [13], [16], [17]. Across disciplines, adding undeserving authors or excluding deserving authors was considered unethical [14], [33], [36], [50], [68], [70], [71], [77], [90], [109], but was reported to be a practice by 10% to 89% of the respondents [18], [31], [41], [46], [50], [58], [65], [68], [75], [82], [85], [91], [96], [109], [114], [127]. Prestige was an important factor in deciding on authorship, as articles from more prestigious economics journals had more authors and fewer contributors in the acknowledgement then those from less prestigious journals [76]. The reasons for agreeing on inappropriate authorship were similar across disciplines and included the feeling of obligation, crediting past and future relationships, team responsibility, power relations [45], [56], [68]. In two studies that assessed the opinions of physicists and pathologists about ICMJE authorship criteria and authorship guidelines of the American Physical Society (APS), the probability that an additional author would not satisfy APS or ICMJE criteria was 23% vs. 67% for physicists [58], and 45% vs. 65% for pathologists [65].

Journal editors also reported experiences with authorship disputes, from 5% in nursing journals [73] to 30% in journals from a major publisher [113]. Despite the reported prevalence of authorship problems, editors did not consider them to be severe and were confident in their management of the problems [68], [113]. Authorship disputes were reported as an increasing problem for institutions [42], but ethics training at institutions may not have effect on the willingness to engage in giving undeserved authorship [29]. In biomedicine, authors often asked for authorship as a stipulation for sharing data-sets [53].

In medicine, the number of authors who did not satisfy widely accepted ICMJE authorship criteria ranged from less than 1% to 63% [38], [57], [61], [62], [67], [86]. The variation may be due to the difference in counting the third ICMJE criterion (‘Approval of the article before publication’) as satisfied by default [38], [57], [61], [62] or checking if authors really declared on this criterion [67], [86]. The prevalence of undeserving authors also depended on the form of contribution declaration in medical journals: it was 21.5% in the journal with a list of contributions to choose from, 9.5% in the journal that provided for open-ended answers, and only 0.5% in the journal that instructed which and how many contributions are needed for each of the 3 ICMJE authorship criteria [62]. The results of this observations study were confirmed in a randomized study with three different declaration forms in a single general medical journal [86]. Undeserved authorship was considered to have potential adverse effects both for the undeserving author and the co-authors, as well as for patient care [109].

Industry relationship and ghost authorship were other important issues for medical journal. Increasing author affiliations with industry were reported in several studies [64], [97], [99], as well as increased odds for authors reporting financial ties to industry [125]. The prevalence of ghost authorship was reported in the range from 2% to 75% [38], [50], [57], [92], [113]. The highest prevalence was found in clinical trial protocols that were later published [92]. Editors considered that there was an increasing trend of ghost authorship, but did not perceive it as a severe problem in their work [113]. Although a recent study demonstrated increasing acknowledgments of medical writing [123], only 20% of academic medical centers in the USA had policies that explicitly banned ghostwriting [120].

Only a few studies looked at the possible interventions to prevent undeserved authorship. The measures proposed by researchers in medicine were publishing the statements on authors' contributions or limiting the number of authors on a byline [31], [56]. When authors made decision about authorship during planning rather than later stages, the prevalence of undeserving authors was smaller, 23% vs. 47% [30]. Although only 44% nursing faculty members in the USA reported formal teaching to graduate students about authorship credit [70], instruction on authorship criteria may increase awareness of ethical decisions about authorship. In a study that looked at how medical students rated different contributions which were both eligible or not eligible for ICMJE authorship criteria, students without any instruction rated critical revision of the manuscript and final approval significantly lower than students with such instruction [93]. In the cluster analysis of ratings by medical students with or without instruction on ICMJE criteria, physicians, and medical faculty, conception/design, analysis/interpretation, and manuscript drafting clustered together, with final approval clustering only for students with instruction [93].

**Figure 3:**
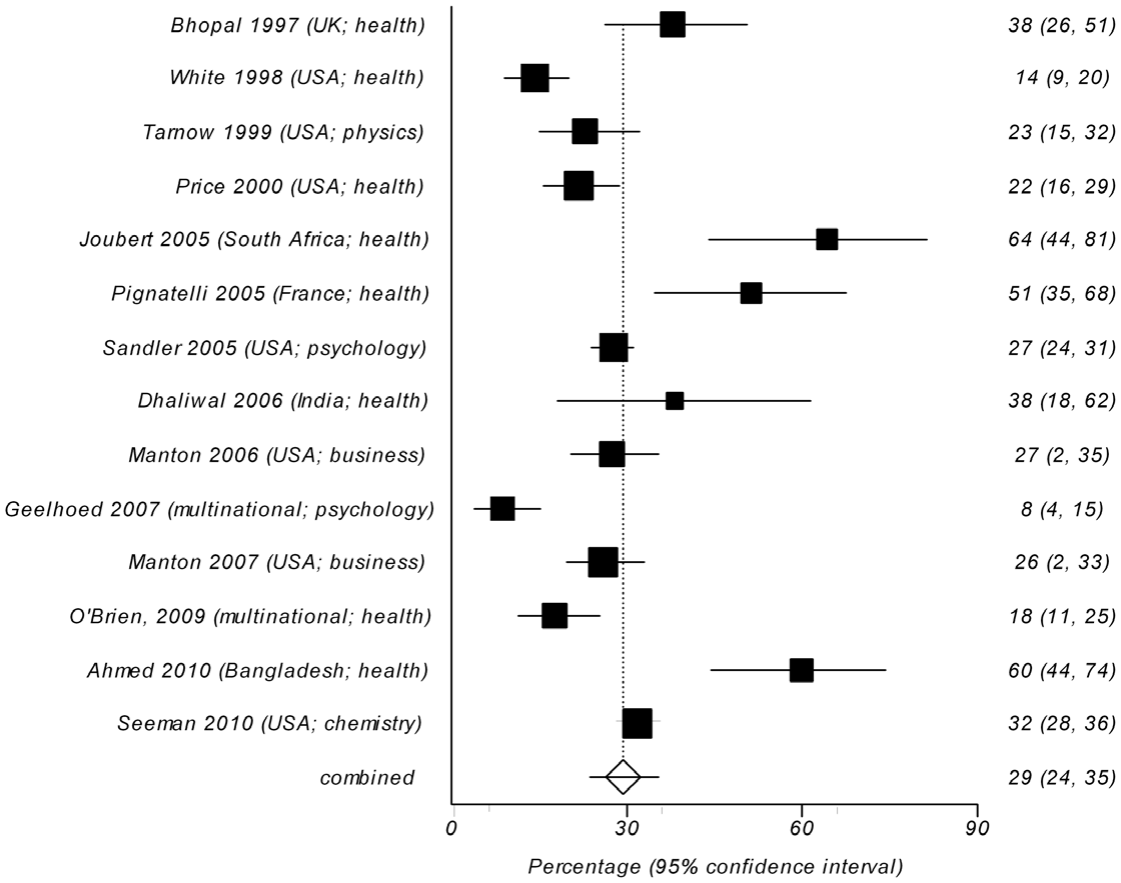
Forest plot of reported rates of problems with or misuse of authorship in self- or non-self reports in 14 survey studies [31], [41], [46], [50], [75], [77], [78], [82], [85], [91], [96], [109], [114], [126****]. The area of a square represent sample size, horizontal lines are 95% confidence interval, diamond and vertical dotted line show the pooled weighted estimate.

**Figure 4:**
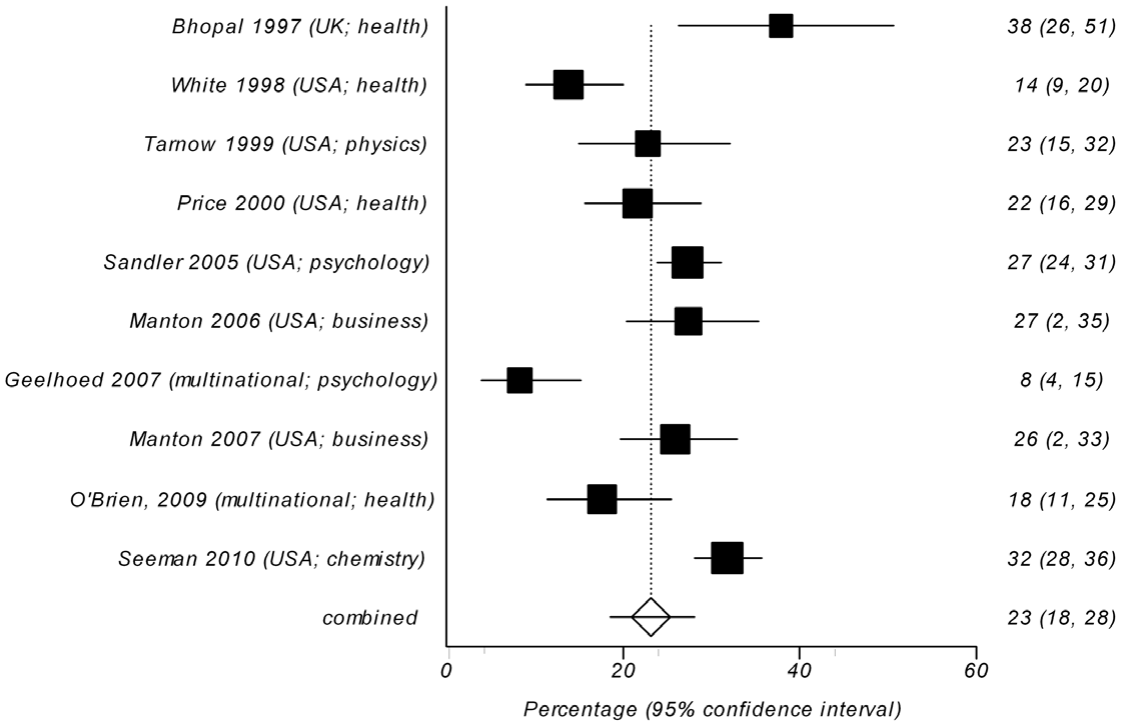
Forest plot of reported rates of problems with or misuse of authorship in self- or non-self reports in 12 survey studies from USA, UK or international journals [**31]**, [41], [46], [50], [78], [85], [109], [126****]. The area of a square represent sample size, horizontal lines are 95% confidence interval, diamond and vertical dotted line show the pooled weighted estimate.

**Figure 5:**
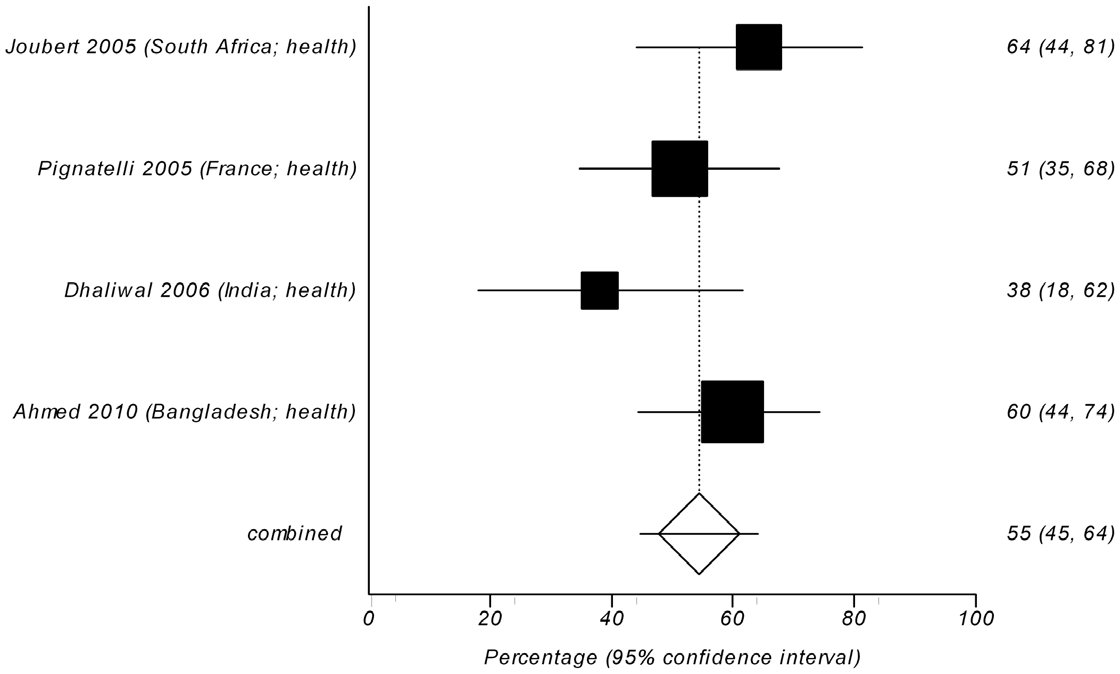
Forest plot of reported rates of problems with or misuse of authorship in self- or non-self reports in 4 survey studies from South Africa, France, India, or Bangladesh [**75]**, [77], [82], [114****]. The area of a square represent sample size, horizontal lines are 95% confidence interval, diamond and vertical dotted line show the pooled weighted estimate.

Fourteen survey studies asked the participants if they personally experienced problems and/or misuse of authorship or observed it for other colleagues [31], [41], [46], [50], [75], [77], [78], [82], [85], [91], [96], [109], [114], [126]. Between 1.5% and 71% of respondents replied affirmatively (crude unweighted mean = 31%, 95% CI = 21% to 41%). Meta-analysis yielded a pooled weighted estimate of 29% (95% CI 24% to 35%), with significant heterogeneity (Cochran's Q = 11.26, df = 13, *P*<0.0001) (Figure 3). The indicators of publication bias were not significant (Harbord bias = 1.54 (92.5% CI −1.83 to 4.91), *P* = 0.391). There was no difference in reported prevalence between studies from health and non-health research fields (W = 36; Z = −1.16; *P* = 0.245; inverse variance weighted Mann-Whitney U-test). However, the comparison between the groups of studies with different locations (USA/UK/international journals vs. non-USA/UK) demonstrated that non-USA/UK studies had significantly higher proportion of reported problems with authorship (W = 55; Z = −2.83; P = 0.002; inverse variance weighted Mann-Whitney U-test). Pooled weighted estimate for USA/UK/international studies was 23% (95% CI 18% to 28%) (Figure 4), compared with 55% (95% CI 45% to 64%) for non-USA/UK studies (Figure 5), with significant heterogeneity in the USA/UK/international sample (Cochran's Q = 61.23, df = 9, *P*<0.0001), which persisted even after stratifying studies by location. Non-USA/UK studies were homogeneous (Cochran's Q = 3.98, df = 3, P = 0.264). The indicators of publication bias were not significant for both study groups (Harbord bias = −3.26 (92.5% CI −7.22 to 0.69), *P* = 0.130, for USA/UK/international group and −3.78 (92.5% CI −18.25 to 10.69), *P* = 0.463, for non-USA/UK group).

### Power issues in authorship

The practices and perceptions about authorship decisions in supervisor/professor – student/non-research persons was assessed in 19 studies (Table 4 and Table S6): 11 studies from social sciences [11], [19], [20], [44], [48], [68], [71], [78], [91], [98], [130], 4 studies from the health research field [13], [16], [17], [70], 2 studies from more than one research field [32], [40] and 2 studies from natural sciences [88], [124].

**Table 4:** Authorship in researcher – student/non-researcher collaborations*.

Article	Study population	Study topic
Spiegel, 1970 [11]	Psychologists in USA	Opinion on deserved authorship for students/non-researchers; Preferred outcome for student-professor collaboration
Werley,^a^ 1981 [13]	Nursing professionals in USA	Opinion on deserved authorship for students/non-researchers; Preferred outcome for student-professor collaboration
Waltz,^a^ 1985 [16]	Health professionals in nursing in USA	Opinion on deserved authorship for students/non-researchers
Gay,a 1987 [17]	Educators in nursing in USA	Opinion on deserved authorship for students/non-researchers
Costa, 1992 [19]	Psychology students and faculty in USA	Faculty vs. students views of authorship order for published dissertation with different level of faculty input
Goodyear, 1992 [20]	Editorial board members and authors of psychology journals in USA	Reported critical incidents related to student research
Brown-Wright, 1997 [32]	Graduate assistants and faculty members in USA	Assistance in analysis of research data warrants authorship for graduate assistant – faculty vs. Assistants
Rose, 1988 [40]	Graduate students in physics, biological, engineering and social sciences in USA	Opinion on deserved authorship for students; Perceived reporting of authorship problems
Louw, 1999 [44]	Academic and non-academic psychologists and masters' degree students in South Africa	Deserving first authorship by academics, non-academics and students
Bartle, 2000 [48]	Faculty and students from psychology departments in USA	Agreement of faculty vs. students on authorship from student-faculty collaboration
Meyer, 2004 [68]	Editorial members of accounting journals and young accounting faculty members in USA	Perceived behaviour appropriateness/behaviour occurrence/actual knowledge of occurrence of co-authorship issues between faculty and students
Szirony, 2004 [70]	Nursing faculty members in USA	Opinions on unethical authorship in student-professor collaboration
Apgar, 2005 [71]	Members of Society for Social Work and Research in USA	Opinions on unethical authorship in student-professor collaboration
Sandler, 2005 [78]	APA members and students with a publication from student-faculty collaboration in USA	Involvement in and reporting of perceived unethical or unfair authorship assignment
Weltzin, 2006 [88]	Participants of ecology meeting in USA	Opinion on first authorship in student-professor collaboration
Geelhoed, 2007 [91]	Authors of articles in clinical psychology journals	Opinion of students vs. faculty on influences on authorship decision making
Tryon, 2007 [98]	Doctoral students in school psychology in USA	Different opinions on first authorship in publications from dissertations
Picard, 2010 [124]	Students and supervisors from agriculture school in Australia	Agreement on authorship issues between students and professors
Welfare, 2010 [130]	Students and faculty from US universities with graduate studies in education	Opinion of students vs. faculty for common and recommended practices in authorship

*Abbreviations: APA, American Psychological Association.

a Partial or full replication or modification of questionnaire by Spiegel and Keith Spiegel, 1970 [11].

Fairness of the research collaboration between professor-supervisor and a student was an important issue in psychology. Surveys since 1970 showed that psychologists generally regard students as sufficiently expert to warrant the 1^st^ authorship on their master or doctoral theses, even when faculty makes significant contribution to the work and manuscript writing [11], [19], [44], [48], [98]. They also generally regarded that any collaborator, regardless of their position or payment for the work, deserved authorship if they made substantial contribution to most aspects of research and writing [11]. Similar perceptions were reported in nursing [13], [16], [17], [70],multidisciplinary areas [32], [40], accounting research [68], social work [71], ecology [88], agriculture, and education research [130].

Using critical incident technique, psychologists identified “taking other's ideas or manuscripts”, “failure to give credit” and “giving unwarranted credit” as most important problems in faculty-student collaboration [20]. Doctoral students in psychology considered it more desirable and ethical for a student to develop the dissertation idea and also though that it was desirable and ethical for the student rather than advisor to be first authors [98]. Although authorship problems occurred [40], [68], [78], students were not likely to, or considered it effective to talk to the dean, file a complaint or contact a journal [40]. The reported reasons for no action were fear of negative consequences, events instigated by respondent, or incident not reaching the level of importance [78]. More psychology students than faculty thought that power differences influenced authorship and saw themselves as having less power than other authors [91]. For students in education research, all recommended authorship practices in offered scenarios was greater than perceived practice [130]. Also, students put a significantly higher authorship value to the research tasks usually given to students, such as collection of qualitative data, entering data into statistical program or analyzing them, writing literature review for the introduction section or writing methods section, and the total time spent on a project.

## Discussion

To the best of our knowledge, this is the first systematic review of research on authorship across all scholarly disciplines. Our search did not identify any systematic review in individual disciplines, although there were a number of overviews and theoretical discussions, including the recent series of the authorship history, current practices, and educational activities in social sciences, engineering and biomedical and life sciences [133]–[137]. The review of 118 studies reported in 123 articles revealed the absence of experimental research on authorship but also outlined our current knowledge about authorship across research disciplines. The available evidence demonstrated the diversity of authorship perceptions but also universal themes: there was a common perception that the conception of research/research design and writing the manuscript were the most important qualifying contributions for authorship – across disciplines, geographical regions and time. Also, respondents from most disciplines would grant authorship not only to the researchers but also to all members of the research team who had made an important contribution. Authorship order emerged as an important but formally undefined issue across disciplines, with clear difference between the minority enforcing alphabetical authorship, such as economy research, and the majority allocating the position on the byline according to the type and quantity of contribution. Power issues in authorship, especially in regard to the relationship between the supervisor/professor and students or non-research members of the team were particularly important in social sciences. Taking other's ideas or manuscripts, failure to give credit and giving unwarranted credit were identified as most important problems in faculty-student collaboration but were rarely reported.

Ethical issues in authorship were common to all disciplines. For the subset of 14 studies that reported results of surveys asking researchers about their own or others' experience of problems with or misuse of authorship, we were able to perform a meta-analysis, the first such analysis for authorship. On average, 29% of the respondents acknowledged such experience. This prevalence of ethical problems in authorship is more than 10-fold greater than the 2% prevalence of research misconduct of fabrication, falsification or data modification, reported in the recent meta-analysis [7]. While authorship misuse is not considered misconduct but a ‘questionable research practice’ by many official research integrity bodies, including the Office of Research Integrity (ORI) in the USA [138], the prevalence estimated in our meta-analysis indicates that authorship problems may have a greater impact on research than ‘classical’ misconduct activities of fabrication, falsification and plagiarism. Furthermore, it can be argued that omitting or adding authors on an article represents falsification or fabrication which directly damages the integrity of the research process, particularly because authorship credit is the foundation of career advancement, esteem in scientific community and funding for research [133]. Although authorship as a research topic is dominant in biomedicine and health [132], we did not find differences in reported problems with authorship between studies from health and other areas. However there was a clear difference between 23% authorship misuse prevalence reported in surveys conducted in the USA or UK settings or international journals with dominant US/UK authorship [91], [109] and 55% in settings outside of USA and UK, from France to South Africa and Bangladesh and India. The reasons why authorship problems are more prevalent in some countries and not in others is not clear. While the USA has two formal bodies to oversee and direct research integrity activities [139], [140], UK does not have a formal body [141], so official structures for preventing misconduct could not be an explanation for the observed difference. France, as most of the countries in Europe except for Scandinavian countries [142], does not have such national bodies, and we could find no evidence for similar national bodies in South Africa, Bangladesh and India. A possible explanation for the high prevalence of authorship misuse in these countries may rather be their position in the mainstream science, either because of the smallness of their scientific communities or language barriers [143].

The results of our systematic survey and meta-analysis are limited primarily by the poor methodological quality of retrieved studies and their heterogeneity. Of the 118 studies, 95 (81%) were either surveys or descriptive studies. Many studies did not report on the construction and pre-testing of surveys of their sampling frames and often with unclear or incomplete reporting of study findings; examples include the lack of interval range for Likert scales and reporting of only means without measures of variability. There were only 8 studies that evaluated some kind of intervention in authorship [86], [90], [93], [94], [102], [106], [121] but all had methodological limitations, so the conclusions on the effects sizes of any intervention to promote responsible authorship practices were not possible. The two single-blinded randomized studies [86], [102] and a test-retest study [94] of authorship declarations demonstrated that currently used forms for declaring authorship contributions as defined by the ICMJE criteria [131], most widely accepted in biomedical and health fields [4], [132], [136], were not reliable instruments to make conclusions on authorship. They also indicated that were several cognitive problems involved in reporting authorship contributions either for oneself or for others. This may in part explain the findings from several studies that researchers often were not familiar with ICMJE criteria or thought that they were not realistic or fair [34], [39], [41], [65], [66], [77]. These findings were also confirmed by qualitative studies, which identified issues in authorship that could not be addressed by normative instructions provided by formal authorship definitions and policies [34], [79], [101], [104], [116], [128].

We deliberately performed a systematic review with a wide scope, sensitive but not specific search, inclusive of all study designs and focused on mainstream publications in international bibliographical indexes because we wanted to provide the synthesis of existing evidence in all research fields and to identify gaps in knowledge. Despite the limitations of the review and retrieved evidence, the results provide an outline of common themes for future research across disciplines. To study authorship definitions, perceptions and practice, there appears to be little scope for conducting more small descriptive surveys or descriptive studies with heterogeneous methodology. To understand how authorship credit is awarded, we may benefit from methodologically rigorous qualitative studies, as well as studies to identify sociological factors associated with authorship and its use and misuse. All these studies would be more powerful if they were conducted across multiple sites and disciplines. This would be particularly relevant to address the observed differences in prevalence of authorship misuse among different geographical settings in the meta-analysis. Testing different sample characteristics in larger, multi-site studies with standardized methodology may reveal important correlates of misconduct in authorship.

As the evidence shows that decisions on authorship are often not made according to the official criteria, there is a need for research into the role of moral vs. normative judgments on authorship [144]. Our recent analysis of authorship statements and definitions in scholarly journals and ethics codes of professional organizations showed that the tone of authorship statements in journals was mostly aspirational, formulating suggestions for best or desired practices, while the statements in ethics codes predominantly used a normative language, conveying minimal standards for practice in authorship [145]. Further research into these differences may provide better tools to promote the moral autonomy of individual researchers and an environment where ethical behaviour in authorship is the norm.

The nature of authorship decisions is also relevant for educational interventions to promote integrity in authorship, which is a rather neglected area both in education and in research [133]. For example, if authorship issues are exclusively a matter of convention, then educational interventions should aim at informing students about authorship criteria and providing opportunities for applying them in practice. If, on the other hand, authorship is, at least partially, a moral issue, then educational interventions targeting moral judgment would be more appropriate [146], [147].

Research avenues outlined here are not possible without collaboration among different stakeholders and across geographical regions and research disciplines. Given the social responsibility of science and its collective impact on human lives, regardless of the discipline, professional development for responsible authorship and other aspects of research should be subjected to the same valid and rigorous forms of evaluation and testing expected for health interventions, such as medicines and medical devices.

## Supporting Information

Table S1 Data extraction form.
(DOC)

Table S2 Overview of included studies.
(DOC)

Table S3 Results of studies addressing the definition of authorship, contributions for deserved authorship and authorship practices.
(DOC)

Table S4 Results of studies addressing the order of authors on the byline.
(DOC)

Table S5 Results of studies addressing ethical authorship practices.
(DOC)

Table S6 Results of studies addressing authorship in researcher – student/non-researcher collaborations.
(DOC)
