# Supplementary material for: A Systematic Review of Research on the Meaning, Ethics and Practices of Authorship across Scholarly Disciplines
Source: PLoS One. 2011 Sep 8;6(9):e23477. doi: 10.1371/journal.pone.0023477 (PMC3169533; doi:10.1371/journal.pone.0023477)
Supplement: Table S1 — Data extraction form. (DOC) [file pone.0023477.s001.doc]

**Table S1.** Data extraction form: systematic review of authorship research

Reference number:

Reviewer initials:

Publication details:

First author:

Journal title and year:

Volume and first page:

Language, if not English:

**Study type:**

- Descriptive
- Before and after (no control)
- Before and after (with control)
- Randomised controlled trial
- Any other

**Aim of the study:**

**Intervention:**

**Outcome measures:**

**Setting:**

Name of COUNTRY:

Type of environment:

Other descriptors of the academic setting:

Total study period:

If relevant:

“Before” period

**Should this study be included in the review?**

Yes

No

Maybe

Reasons for No or Maybe:

**Participants:**

**Outcome:**

| Outcome | How defined? | How measured? | Comments |
| --- | --- | --- | --- |
|  |  |  |  |
|  |  |  |  |
|  |  |  |  |

**Confounders:**

|  | Tick if controlled/restricted for | Describe how controlled for (and/or other comments) |
| --- | --- | --- |
|  |  |  |
|  |  |  |
|  |  |  |

Quality Scale

|  | Quality score | Explanation of score |
| --- | --- | --- |
| Was the intervention group representative of the population to which the results are generalised? |  | 0 = no randomisation  1 = quasi randomised  2 = randomised |
| Were the comparison/control groups representative of the intervention groups? |  | 0 = no control groups  1 = control groups poorly representative of intervention groups  2 = control groups adequately representative |
| Was there an objective and valid measure of outcome used? |  | 0 = Outcome poorly described or measured  1 = Partially described or valid  2 = Well described and valid |
| Were all the results provided with estimates of uncertainty (ie. Confidence intervals) and statistical significance? |  | 0 = none  1 = some  2 = all |
| Were the main conclusions based on primary study hypotheses? |  | 0 = none  1 = some  2 = all |
| Were all the important confounders measured and controlled for? |  | 0 = no or poorly controlled  1 = partially controlled  2 = adequately controlled |
| TOTAL SCORE |  | 9-12 = good quality  6-8 = average quality  0-5 = poor quality |

**Describe any other important sources of bias:**

**Results:**

| Outcome | How defined? | No. | Crude results (95% CI) | Adjusted results (95% CI) | Adjusted for which confounders? |
| --- | --- | --- | --- | --- | --- |
|  |  |  |  |  |  |
