# Supplementary material for: A Systematic Review of Research on the Meaning, Ethics and Practices of Authorship across Scholarly Disciplines
Source: PLoS One. 2011 Sep 8;6(9):e23477. doi: 10.1371/journal.pone.0023477 (PMC3169533; doi:10.1371/journal.pone.0023477)
Supplement: Table S2 — Overview of included studies. (DOC) [file pone.0023477.s002.doc]

**Table S2.** Characteristics of included studies (studies are in chronological order; only study outcomes relevant for authorship are listed, as some studies also addressed issues other than authorship)*

1. **Zuckerman H, 1967, American Sociological Review**

| Discipline | Multidisciplinary |
| --- | --- |
| Methods | Descriptive study: interviews of Nobel laureates and comparison with matched scientists |
| Data/Sample | 40 pairs of Nobel laureates in the USA and scientists matched in age, field of specialization, type of organizational affiliation, and initial letter of last name, drawn from American Men of Science |
| Age | Not stated |
| Response rate | 74.5% for laureates (41/55) and 44.7% for the scientists (55/123) before matching |
| Comparisons | Scientists matched in age, field of specialization, type of organizational affiliation, and initial letter of last name |
| Outcomes | Position on the author byline in published papers |
| Methodological limitations | † |
| Notes |  |

1. **Zuckerman H, 1968, American Journal of Sociology**

| Discipline | Multidisciplinary |
| --- | --- |
| Methods | Descriptive study: interviews of Nobel laureates |
| Data/Sample | 41 out of 55 Nobel laureates living in the USA |
| Age | Not stated |
| Response rate | 74.5% |
| Comparisons | Authors of a random sample of articles listed in Science Abstracts (physics), Chemical Abstracts and Biological Abstracts from 1920 to 1964 |
| Outcomes | Pattern of name ordering on scientific articles by Nobel laureates |
| Methodological limitations | † |
| Notes | The same data set as in Zuckerman H, 1967, but the paper is not cited (unpublished PhD thesis cited) |

1. **Over and Smallman, 1970, Nature**

| Discipline | Health: Medicine (biomedicine) |
| --- | --- |
| Methods | Descriptive study |
| Data/Sample | 1023 articles published in *The Journal of Physiology* in 1961-1964 |
| Age | NA |
| Response rate | NA |
| Comparisons | NA |
| Outcomes | Fraction of articles from authors with surnames beginning with P-Z |
| Methodological limitations | Sample not well described, results not presented in detail (report in a letter form) |
| Notes | The journal from the study had a policy of alphabetical listing of authors. |

1. **Spiegel and Keith-Spiegel, 1970, American Psychologist**

| Discipline | Social Sciences: Psychology |
| --- | --- |
| Methods | Descriptive study: postal questionnaire survey |
| Data/Sample | A random sample of 1000 American Psychology Association (APA) members from 1966 directory, all psychologist with ≥5 publications in the 1967 Index of Psychological Abstracts (n=261) and psychologists employed by Veterans Administration in the USA (n=260) |
| Response rate | 49% |
| Age | Not stated |
| Comparisons | NA |
| Outcomes | Opinion on deserved authorship in 25 case scenarios, including contributions of students, assistants and paid researchers  Opinion on authorship order  Proposed solutions to multiple authorship  Effect of publication rate on assignment of publication credit |
| Methodological limitations | No details on how the questionnaire was created and whether it was pre-tested, or on when the survey was performed. |
| Notes |  |

1. **Bridgwater CA et al, 1981, American Psychologist**

| Discipline | Social Sciences: Psychology |
| --- | --- |
| Methods | Descriptive study: postal questionnaire survey |
| Data/Sample | 948 academics from 40 randomly selected USA doctoral programs in psychology |
| Age | Not stated |
| Response rate | 27.5% |
| Comparisons | NA |
| Outcomes | Opinion on deserved authorship in case scenarios  Opinion on authorship order |
| Methodological limitations | Low response rate; no details on the sample or on when the survey was performed; no numerical data presented on the opinion on authorship order, only overall percentage of respondents’ agreement for case scenarios presented. |
| Notes | Partial replication of the study by Spiegel and Keith-Spiegel, 1970. |

1. **Werley HH et al, 1981, Research in Nursing and Health**

| Discipline | Health: Nursing |
| --- | --- |
| Methods | Descriptive study: postal questionnaire survey |
| Data/Sample | A random sample of 1693 nursing professionals in the USA |
| Age | Not stated |
| Response rate | 57.9% |
| Comparisons | Subgroup comparison of researchers vs. others and doctoral students vs. others |
| Outcomes | Opinion on deserved authorship in case scenarios  Opinion on authorship order, including contributions of students, assistants and paid researchers  Items with differences in answers for researchers or doctoral students vs. others |
| Methodological limitations | No details on questionnaire adaptation or on when the survey was performed. |
| Notes | Case scenarios developed according to Spiegel and Keith-Spiegel, 1970. |

1. **von Glinow and Novelli, 1982, Academy of Management Journal**

| Discipline | Social Sciences: Business |
| --- | --- |
| Methods | Descriptive study: postal questionnaire survey |
| Data/Sample | 315 professionals affiliated with 21 management journals or Academy of Management division chairpersons or program chairpersons in the USA |
| Age | Not stated |
| Response rate | 48% |
| Comparisons | Subgroup comparison of editors vs. editorial review board members |
| Outcomes | Data collection as deserved authorship  Ordering names on articles  Ethical issues of adding a prestigious name which affects publication |
| Methodological limitations | No details on sample generation (reasons for the choice of journals), on how the questionnaire was created and whether it was pre-tested, or on when the survey was performed; low response rate; only fractions presented, without raw numbers |
| Notes |  |

1. **Over R, 1982, American Psychologist**

| Discipline | Social Sciences: Psychology |
| --- | --- |
| Methods | Descriptive study |
| Data/Sample | Articles in 13 APA psychology journals in 1949, 1959, 1969, and 1979 (*American Psychologist, Journal of Abnormal Psychology, Journal of Applied Psychology, Journal of Comparative and Physiological Psychology, Journal of Consulting and Clinical Psychology, Journal of Educational Psychology, Journal of Experimental Psychology, Psychological Bulletin, Psychological Review, Journal of Counseling Psychology, Journal of Personality and Social Psychology, Developmental Psychology, Professional Psychology*) |
| Age | NA |
| Response rate | NA |
| Comparisons | NA |
| Outcomes | Fraction of alphabetical or non-alphabetical sequencing of authors on published articles |
| Methodological limitations | † |
| Notes |  |

1. **Waltz CF eat al, 1985, Nursing Outlook**

| Discipline | Health: Multidisciplinary |
| --- | --- |
| Methods | Descriptive study: postal questionnaire survey |
| Data/Sample | A random sample of 400 US health professionals from nursing, dentistry, medicine, pharmacy, and social work, who published articles in 10 refereed journals |
| Age | Not stated |
| Response rate | 47.7% |
| Comparisons | Şubgroup comparison of nurses with other health professionals |
| Outcomes | Opinion on deserved authorship in 25 case scenarios, including contributions of students, assistants and paid researchers  Opinion on authorship order  Ethical issues in authorship  Agreement of nurses with other health professionals on opinion in case scenarios |
| Methodological limitations | No details on random sample generation, on questionnaire modification and pre-testing, or on when the survey was performed; low response rate; results presented as percentages only, and only for the nursing subsample |
| Notes | Case scenarios developed according to Spiegel and Keith-Spiegel, 1970.  Only the data on the nursing sample is included in the survey as they were reported as numerical data. |

1. **Gay JT et al, 1987, IMAGE: Journal of Nursing Scholarship**

| Discipline | Health: Nursing |
| --- | --- |
| Methods | Descriptive study: postal questionnaire survey |
| Data/Sample | 431 faculty members of 18 USA schools with doctoral programs in nursing |
| Age | Not stated |
| Response rate | 53% |
| Comparisons | There was comparison between different academic ranks, but data not provided |
| Outcomes | Ethical issues in authorship  Opinion on authorship order  Authorship in student/non-professional – academic collaboration  Proposed solutions to multiple authorship |
| Methodological limitations | No details on questionnaire modification and pre-testing, or on when the survey was performed; only factions presented instead of raw numbers; no data for comparisons between academic ranks subgrups |
| Notes | The study used a modified version of the questionnaire from Spiegel and Keith-Spiegel, 1970. |

1. **van der Kloot and Willemsen, 1991, Nederlands Tijdschrift voor de Psyhologie**

| Discipline | Social Sciences: Psychology |
| --- | --- |
| Methods | Descriptive study: on-site and postal questionnaire survey |
| Data/Sample | 63 participants of the national conference of social-psychological society of researchers in the field of psychometrics, in the Netherlands |
| Age | Not stated |
| Response rate | 63% |
| Comparisons | Subgroup comparison between social psychologists and psychometrics |
| Outcomes | Assessment of 5 types of contributions to a research project: research design, report writing, leadership of the project, data gathering and data analysis  Prevalence of disagreement on authorship with colleagues |
| Methodological limitations | No details on how the questionnaire was created and whether it was pre-tested, or on when the survey was performed. |
| Notes | In Dutch language |

1. **Costa and Gatz, 1992, Psychological Science**

| Discipline | Social Sciences: Psychology |
| --- | --- |
| Methods | Descriptive study: postal questionnaire survey |
| Data/Sample | 234 faculty and 628 students from 7 geographically diverse psychology departments with PhD programs in the USA |
| Age | Not stated |
| Response rate | 53% response rate for faculty and 49% for students |
| Comparisons | Subgroup analysis of faculty and students |
| Outcomes | Opinion on authorship in a case vignette, including contributions of students, assistants and paid researchers  Opinion on how their colleagues would respond to the same vignette |
| Methodological limitations | No details on sample formation (reasons for the choice of 7 departments), on how the vignettes were created and whether it was pre-tested, or on when the survey was performed. |
| Notes |  |

1. **Goodyear RK et al, 1992, Professional Psychology: Research and Practice**

| Discipline | Social Sciences: Psychology |
| --- | --- |
| Methods | Descriptive study: postal questionnaire survey |
| Data/Sample | 126 editorial board members of 3 APA journals (*Journal of Counseling Psychology, Psychological Assessment, Professional Psychology: Research and Practice*) and authors in 2 of these journals (*Journal of Counseling Psychology, Psychological Assessment*) |
| Age | Mean 45 years (SD 9.6) |
| Response rate | 45.2% |
| Comparisons | NA |
| Outcomes | Instances of ethical problems related to authorship issues related to student research |
| Methodological limitations | Reasons for choice of the samples (journals and authors) not presented in detail; low response rate; no details on when the study was performed; numerical results not presented but only reported critical incidents classified |
| Notes | Flanagan’s (1954) critical incident technique was used to identify categories of possible ethical problems that experienced psychologists identify as important |

1. **McCarl BA, 1993, Review of Agricultural Economics**

| Discipline | Social Sciences: Economics |
| --- | --- |
| Methods | Descriptive study |
| Data/Sample | 15225 citations from 5 US journals of agricultural economics 1988-1990 (*American, North Central, Northeastern, Southern and Western Journals of Agricultural Economics*) |
| Age | NA |
| Response rate | NA |
| Comparisons | NA |
| Outcomes | Presence of bias in citations according to the alphabetical position of the author’s surname on the authors’ byline. |
| Methodological limitations | No details on the journal sample |
| Notes |  |

1. **Shulkin DJ et al, 1993, Academic Medicine**

| Discipline | Health: Medicine |
| --- | --- |
| Methods | Descriptive study |
| Data/Sample | Publications of 233 former chairs of departments of medicine from 1979 to 1990 |
| Age | NA |
| Response rate | NA |
| Comparisons | Long- vs. short-term (<10 years) chairs |
| Outcomes | Number of articles with chair as the last author |
| Methodological limitations | No demographic details on the sample |
| Notes |  |

1. **Diguisto E, 1994, Social Science and Medicine**

| Discipline | Health: Medicine (biomedicine) |
| --- | --- |
| Methods | Descriptive study: in-house questionnaire survey |
| Data/Sample | 28 staff members at a research center in Australia |
| Age | Not stated |
| Response rate | 89% |
| Comparisons | NA |
| Outcomes | Rating of 13 possible contributions required for authorship |
| Methodological limitations | Small sample size; no details on the development of the questionnaire and its pre-testing; no details on the sample; no details on when the study was performed |
| Notes |  |

1. **Floyd SW et al, 1994, Academy of Management Journal**

| Discipline | Social Sciences: Business |
| --- | --- |
| Methods | Descriptive study: postal questionnaire survey |
| Data/Sample | 241 authors of articles published in 3 management journals (*Academy of Management Journal, Academy of Management Review, Administrative Science Quarterly*) from January 1988 to April 1990 |
| Age | NA |
| Response rate | 61% |
| Comparisons | NA |
| Outcomes | Constructs for deciding on authorship |
| Methodological limitations | No details on the sample (choice of journals); no details on the content of the questionnaire; no details on when the study was performed; no numerical data from the survey presented but their principal component analysis with varimax rotation |
| Notes |  |

1. **Goodman NW, 1994, BMJ**

| Discipline | Health: Medicine |
| --- | --- |
| Methods | Descriptive study: postal questionnaire survey |
| Data/Sample | 14 first authors of research articles with 3, 7 or more authors in a general medical journal in 1993 |
| Age | NA |
| Response rate | 85.7% |
| Comparisons | NA |
| Outcomes | Contributions identified by the first as relevant for authorship of all authors |
| Methodological limitations | Small sample size; no details on when the study was performed or which journal was studied |
| Notes |  |

1. **Shapiro DW et al, 1994, JAMA**

| Discipline | Health: Medicine |
| --- | --- |
| Methods | Descriptive study: postal questionnaire survey |
| Data/Sample | US authors from a consecutive sample of 200 papers with ≥4 authors in 10 leading biomedical journals (basic science: *American Journal of Physiology, Cell, Journal of Biological Chemistry, Nature, Science*; medical journals: *Annals of Internal Medicine, Circulation, Gastroenterology, JAMA, The New England Journal of Medicine*) |
| Age | NA |
| Response rate | 92% |
| Comparisons | Subgroup analysis of basic vs. clinical authors, multicenter trial authors vs other authors |
| Outcomes | First author’s ratings on which authors made substantial contributions in 7 categories |
| Methodological limitations | No details on when the study was performed, numerical data not presented for some parameters |
| Notes |  |

1. **Wagner MK et al, 1994, Psychological Reports**

| Discipline | Social Sciences: Psychology |
| --- | --- |
| Methods | Descriptive study: postal questionnaire survey |
| Data/Sample | 197 single, first or second authors of 102 articles in a single journal (Journal of Consulting and Clinical Psychology), with more than 1 author |
| Age | NA |
| Response rate | 61% |
| Comparisons | NA |
| Outcomes | Relative values given to contributions for authorship  Relative values of research activities for different author position |
| Methodological limitations | No details on when the study was performed |
| Notes |  |

1. **Davies at al, 1996, Canadian Medical Association Journal**

| Discipline | Health: Medicine |
| --- | --- |
| Methods | Descriptive study: cross-sectional postal and telephone questionnaire survey |
| Data/Sample | Chairs of 16 Canadian university departments of paediatrics and dean’s offices of 16 university medical faculties |
| Age | Not stated |
| Response rate | 15/16 department chairs, 16/16 dean’s offices |
| Comparisons | NA |
| Outcomes | Weight assigned by department chairs to contributions to published research according to author’s position on the byline  Practices in indicating contributions for promotion |
| Methodological limitations | No details on when the study was performed; small sample size |
| Notes |  |

1. **Eastwood et al, 1996, Science and Engineering Ethics**

| Discipline | Multidisciplinary |
| --- | --- |
| Methods | Descriptive study: postal questionnaire survey |
| Data/Sample | 1005 postdoctoral fellows registered with the Office of Research Affairs of the University of California San Francisco, USA, 1992 |
| Age | Not stated |
| Response rate | 33% |
| Comparisons | NA |
| Outcomes | Rank of criteria for justifying authorship  Experiences with authorship problems  Future conduct in assigning authorship |
| Methodological limitations | Low response rate; no details on when the study was performed; number of respondents for individual questions not presented |
| Notes |  |

1. **Slone RM, 1996, American Journal of Roentgenology (ARJ)**

| Discipline | Health: Medicine |
| --- | --- |
| Methods | Descriptive study: postal questionnaire survey |
| Data/Sample | First authors of 275 papers from USA institutions published in *ARJ* in 1992 and 1993 |
| Age | NA |
| Response rate | 72% |
| Comparisons | NA |
| Outcomes | Description of co-authors’ contributions by the first author  Prevalence of undeserved authorship |
| Methodological limitations | † |
| Notes |  |

1. **Bhopal et al, 1997, BMJ**

| Discipline | Health: Medicine |
| --- | --- |
| Methods | Descriptive study: interviews with staff |
| Data/Sample | 66 staff from university medical faculty in Great Britain, stratified sample |
| Age | Not stated |
| Response rate | 94% |
| Comparisons | NA |
| Outcomes | Awareness and use of criteria for authorship  Views of which contributions to research merit authorship  Perceptions of gift authorship  Experiences of authorship problems |
| Methodological limitations | No details on when the study was performed |
| Notes |  |

1. **Brown-Wright DA et al, 1997, Journal of College Student Development**

| Discipline | Multidisciplinary |
| --- | --- |
| Methods | Descriptive study: interview |
| Data/Sample | 151 graduate assistants and 72 faculty members assigned at least one graduate assistant in USA |
| Age | Not stated |
| Response rate | not known (data not provided) |
| Comparisons | NA |
| Outcomes | Contributions for graduate assistants required for inclusion as an author in a publication |
| Methodological limitations | No details on the sample selection and characteristics; no details on when the study was performed; only frequencies presented instead of raw data |
| Notes |  |

1. **Hamilton and Greco, 1997, Journal of Education for Business**

| Discipline | Social Sciences: Multidisciplinary |
| --- | --- |
| Methods | Descriptive study: on-site university questionnaire survey |
| Data/Sample | 200 faculty members (61 business and 139 non-business) from a large southern university in the USA |
| Age | Not stated |
| Response rate | 44.5% (59% for business and 38% for non-business faculty) |
| Comparisons | Subgroup analysis of business and non-business faculty |
| Outcomes | Knowledge of unethical authorship practices among colleagues  Attitude towards inclusion of a non-contributing author  Attitudes towards which contributions deserve authorship, including that of students  Answers to case vignettes about inclusion of non-contributing authors for different purposes |
| Methodological limitations | Low response rate; no details on the sample characteristics; no details on when the study was performed |
| Notes | Non-business faculty included psychology, communicative disorders, history/philosophy, music, sociology, English, foreign languages, political science, communication, and criminal justice |

1. **Netting and Nichols-Casebolt, 1997, Journal of Social Work Education**

| Discipline | Social Sciences: Social work |
| --- | --- |
| Methods | Qualitative study |
| Data/Sample | 2 faculty (n=15 each) and 1 student (n=6) focus groups in a USA university (Virginia Commonwealth University) |
| Age | Not stated |
| Response rate | NA |
| Comparisons | NA |
| Outcomes | Emerging issues in authorship:  1. professional socialization and acculturation (experiences with authorship, value of coauthorship)  2. professional development and growth (factor influencing perceived change in authorship issues)  3. Negotiation/renegotiation (rules when to include someone as an author)  4. Professional responsibility (to co-authors, students, profession) for accuracy and accountability of one’s work |
| Methodological limitations | No explicit theoretical framework; vague aim and objectives; data collection poorly described; no details of sampling procedures for doctoral students; not clear which questions were used to elicit responses and discussion; not clear who conducted the focus groups, where the focus groups were conducted, or how long did they last; no attempt to justify the number of conducted focus groups; data analysis procedure not described; no consideration of the relationship between researchers and participants, |
| Notes |  |

1. **Almeida OP, 1998, Revista ABP-APAL**

| Discipline | Health: Multidisciplinary |
| --- | --- |
| Methods | Descriptive study: questionnaire survey |
| Data/Sample | 50 professionals working at a department of mental health in Sao Paulo (20 physicians and 30 non-physicians), Brazil |
| Age | Not stated |
| Response rate | 86% for all (95% (19/20) for physicians and 80% (24/30) for non-physicians) |
| Comparisons | Compared the opinions of medical and non-medical professionals about qualifications for authorship |
| Outcomes | Percent of respondents agreeing with the importance of a contribution for authorship credit |
| Methodological limitations | Small sample size; no details on when the study was performed |
| Notes | Questions were modified from Bhopal at al 1997; in Portuguese. |

1. **Butler and Ginn, 1998, Canadian Journal of Nursing Research**

| Discipline | Health: Nursing |
| --- | --- |
| Methods | Descriptive study: questionnaire survey |
| Data/Sample | Convenience sample (n=375) of Canadian nurses expected to publish scholarly or research work |
| Age | Not stated |
| Response rate | 52% |
| Comparisons | NA |
| Outcomes | Consensus on opinions of deserved authorship case scenarios |
| Methodological limitations | Convenience sample, not clearly described; no details on when the study was performed; sample described with means and ranges; results presented as number of respondents in agreement, no raw numbers |
| Notes | Replication of study by Werley et al, 1981, using modified case scenarios from Spiegel and Keith-Spiegel 1970 |

1. Drenth JPH, 1998, JAMA

| Discipline | Health: Medicine |
| --- | --- |
| Methods | Descriptive study |
| Data/Sample | Authors of original articles published in *BMJ* in 1975, 1980, 1985, 1990, 1995 |
| Age | NA |
| Response rate | NA |
| Comparisons | Comparison of authorship over the years |
| Outcomes | Order of authors per article for 8 categories: professor, department chairperson, consultant, senior registrar, lecturer and/or registrar, medical student, house offices, miscellaneous |
| Methodological limitations | No details on when the study was performed |
| Notes |  |

1. **Flanagin A** et al, 1998, JAMA

| Discipline | Health: Medicine |
| --- | --- |
| Methods | Descriptive study: postal questionnaire survey |
| Data/Sample | 1179 corresponding authors from the USA of articles published in 3 large (*Annals of Internal Medicine, JAMA, The New England Journal of Medicine*) and 3 small medical journals (*American Journal of Cardiology, American Journal of Medicine, American Journal of Obstetrics and Gynecology*) in 1996 |
| Age | NA |
| Response rate | 69% |
| Comparisons | Subgroup comparison between large-circulation general medical and small-circulation specialty journals |
| Outcomes | Prevalence of articles with honorary authors and ghost authors, as reported by corresponding authors |
| Methodological limitations | No details on when the study was performed; non-USA authors excluded |
| Notes |  |

1. **Hoen et al, 1998, JAMA**

| Discipline | Health: Medicine |
| --- | --- |
| Methods | Descriptive study: postal questionnaire survey |
| Data/Sample | 450 authors of 115 articles published in 1995 in the *Nederlands Tijdschrift voor Geneeskunde*, the Netherlands |
| Age | NA |
| Response rate | 78.2% |
| Comparisons | Discrepancy between authors of the same article on each-other’s contribution |
| Outcomes | Author’s contribution to study design, material, collection of data, statistics, and writing |
| Methodological limitations | No details on how the questionnaire was constructed and whether it was pre-tested; no details on when the study was performed; data on discrepancies between reported contributions not clearly presented |
| Notes |  |

1. **Rose and Fischer, 1998, Ethics and Behavior**

| Discipline | Multidisciplinary |
| --- | --- |
| Methods | Descriptive study: postal questionnaire survey |
| Data/Sample | 1289 graduate students in the physical, biological, engineering, and social science fields (psychology students excluded) from a large southeastern university, USA |
| Age | Not stated |
| Response rate | 21% |
| Comparisons | Male and female students |
| Outcomes | Rating the ethics of a professor as first author  Rating the ethics of a professor submitting the manuscript without showing it or discussing the authorship with the student  Likelihood for a dissatisfied student to report the authorship results, and effectiveness or negative consequences of reporting |
| Methodological limitations | Low response rate; no details on when the study was performed |
| Notes |  |

1. **White AH et al, 1998, Nurse Educator**

| Discipline | Health: Nursing |
| --- | --- |
| Methods | Descriptive study: postal questionnaire survey |
| Data/Sample | Convenience sample of 225 first authors from USA of papers on nursing research in 15 journals |
| Age | Not stated |
| Response rate | 74% |
| Comparisons | NA |
| Outcomes | Factors determining author inclusion and ordering  Problems and concerns about author inclusion and ordering  Justifiable reasons for author inclusion |
| Methodological limitations | Convenience sample; no details on when the study was performed or which journals were included |
| Notes | Questionnaire modified from Shapiro et al, 1994. |

1. **Wilcox LJ, 1998, JAMA**

| Discipline | Health: Multidisciplinary |
| --- | --- |
| Methods | Descriptive study |
| Age | NA |
| Data/Sample | Cases of authorship disputes brought to ombuds office at Harvard Medical and Dental Schools, School of Public Health, and affiliated hospitals, USA |
| Response rate | NA |
| Comparisons | NA |
| Outcomes | Change in number of queries related to authorship between 1991-1992 and 1996-1997 |
| Methodological limitations | † |
| Notes |  |

1. **Engers M et al, 1999, Journal of Political Economy**

| Discipline | Multidisciplinary |
| --- | --- |
| Methods | Descriptive study and mathematical modelling |
| Age | NA |
| Data/Sample | Articles from 7 journals: 2 economics (*Journal of Finance, Journal of Economic History*), 1 law (*Yale Law Review*), 2 social sciences (*American Journal of Sociology, American Psychologist*), 1 natural sciences (*Angewandte Chemie*) and 1 medical sciences (*New England Journal of Medicine*) |
| Response rate | NA |
| Comparisons | NA |
| Outcomes | Percent of articles with alphabetical listing of authors  Construction of a bargaining model for name ordering between two co-authors |
| Methodological limitations | No details on the sample (the total number of analyzed articles not stated) |
| Notes |  |

1. **Louw and Fouche, 1999, South African Journal of Psychology**

| Discipline | Social Sciences: Psychology |
| --- | --- |
| Methods | Descriptive study: hand delivered or postal questionnaire survey |
| Data/Sample | 1039 academic psychologists, non-academic psychologists, full time master’s degree students in research, clinical and counselling psychology at 21 universities in South Africa |
| Age | Not stated |
| Response rate | 40.8% |
| Comparisons | Subgroup analysis of academics, non-academics and students |
| Outcomes | Choices for first authorship in 4 case vignettes on supervisor-student collaboration |
| Methodological limitations | Low response rate; no details on when the study was performed |
| Notes |  |

1. **Rose MR, 1999, Science Editing & Information Management**

| Discipline | Multidisciplinary |
| --- | --- |
| Methods | Descriptive study |
| Data/Sample | Ethics statements from 90 scientific professional organization likely to receive NSF funds in the USA |
| Age | NA |
| Response rate | NA |
| Comparisons | NA |
| Outcomes | Number and content of ethics statements regarding authorship |
| Methodological limitations | No details on when the study was performed |
| Notes |  |

1. **Tarnow E, 1999, Science and Engineering Ethics**

| Discipline | Natural Sciences: Physics |
| --- | --- |
| Methods | Descriptive study: postal questionnaire survey |
| Data/Sample | Postdoctoral fellows in physics (99 randomly picked from a mailing list of all postdocs at a very large national laboratory and 92 randomly picked from an American Physical Society (APS) mailing list of all university postdocs) |
| Age | Not stated |
| Response rate | 59% for institution postdocs and 47% for APS postdocs |
| Comparisons | NA |
| Outcomes | Familiarity with APS authorship guidelines  Opinion on deserving authorship  Discussion of authorship with supervisors  Reasons for inappropriate authorship |
| Methodological limitations | No details on how the questionnaire was developed and pre-tested, no details on when the study was performed; only means for ratings presented |
| Notes | Results were averaged for the 2 groups of postdocs |

1. **Yank V and Rennie D, 1999, Annals of Internal Medicine**

| Discipline | Health: Medicine |
| --- | --- |
| Methods | Descriptive study |
| Age | NA |
| Data/Sample | 121 original research articles published in *The Lancet* from July to December 1997 |
| Response rate | NA |
| Comparisons | NA |
| Outcomes | Taxonomy of researcher’s self-reported contributions published at the end of the article |
| Methodological limitations | † |
| Notes |  |

1. **Bartle et al, 2000, Psychological Reports**

| Discipline | Social Sciences: Psychology |
| --- | --- |
| Methods | Descriptive study: postal questionnaire survey |
| Data/Sample | 428 faculty and 438 students from a random sample of 60 psychology departments offering a graduate degree in psychology from 1996 APA Guide to Graduate Study in Psychology, USA |
| Age | Range 22 to 70 years (mean 39.3, SD 10.9) |
| Response rate | 31.5% for faculty and 15.5% for students (23.3% collectively) |
| Comparisons | Comparison of faculty and student perceptions toward authorship assignment process |
| Outcomes | Dimensions used by psychologists in establishing authorship credit in the past  Dimensions psychologists believe should be used in determining authorship credit  Differences in perceptions toward assignment process between faculty and students, including author order |
| Methodological limitations | Low response rate; no details on when the study was performed; little raw data presented, mostly results from principal component analysis with varimax rotation |
| Notes | Some of the vignettes were based on Spiegel and Keith-Spiegel, 1970, and Fine and Kurdek, 1993 |

1. **Hart RL, 2000, The Journal of Academic Librarianship**

| Discipline | Social Sciences: Information Sciences |
| --- | --- |
| Methods | Descriptive study: e-mail questionnaire survey |
| Age |  |
| Data/Sample | 127 co-authors of all multiple-authored articles (n=54) in *College & Research Libraries* (C&RL) and the *Journal of Academic Librarianship* in 1997 and 1998 |
| Response rate | 77.2% |
| Comparisons | NA |
| Outcomes | Prevalence of methods for assigning authorship credit and order on byline |
| Methodological limitations | No details on how the questionnaire was developed and whether it was pretested; some results presented as frequencies or as means without a measure of variability |
| Notes |  |

1. **Price JH et al, 2000, American Journal of Health Behavior**

| Discipline | Health: Multidisciplinary |
| --- | --- |
| Methods | Descriptive study: postal questionnaire survey |
| Data/Sample | Random sample (n=300) from 631 faculty members from all USA institutions granting graduate degree programs in health education (Directory of Institutions Offering Undergraduate and Graduate Degree Programs in Health) |
| Age | Not stated |
| Response rate | 59% |
| Comparisons | Subgroup analysis for faculty from doctoral degree granting and non-granting programs, and highly published vs. not highly published authors |
| Outcomes | Number of criteria perceived as sufficient for manuscript authorship  Special criteria perceived as sufficient for authorship  Perceptions of guest and ghost authorship  Opinions on the role of health education journal in authorship |
| Methodological limitations | No details on when the study was performed |
| Notes |  |

1. **Chambers R et al, 2001, BMJ**

| Discipline | Health: Medicine |
| --- | --- |
| Methods | Descriptive study |
| Data/Sample | 1456 authors of 550 articles with ≥2 authors, published in *BMJ* from August 2000 to July 2001 |
| Age | NA |
| Response rate | NA |
| Comparisons | NA |
| Outcomes | Position of surname relative to authors according to the initial letter of surname |
| Methodological limitations | Only percentages presented for each alphabet letter, size of categories not stated |
| Notes | Articles included editorials and articles (papers, general practice, information in practice, clinical review, and education and debate) |

1. **Phillips SG et al, 2001, American Medical Writers Association (AMWA) Journal**

| Discipline | Health: Medicine |
| --- | --- |
| Methods | Descriptive study: postal questionnaire survey |
| Data/Sample | 1179 corresponding US authors of articles published in 3 large (*Annals of Internal Medicine, JAMA, The New England Journal of Medicine*) and 3 small medical journals (*American Journal of Cardiology, American Journal of Medicine, American Journal of Obstetrics and Gynecology*) in 1996 |
| Age | Mean 46.9 (SD 9.3) |
| Response rate | 69% |
| Comparisons | NA |
| Outcomes | Opinion of medical writers about acknowledgment for their service |
| Methodological limitations | † |
| Notes | Secondary analysis of the data from Flanagin et al, 1998 |

1. **Reidpath DD and Allotey PA, 2001, Bioethics**

| Discipline | Health: Medicine |
| --- | --- |
| Methods | Descriptive study: prospective e-mail questionnaire survey |
| Data/Sample | Authors of 29 articles published in *BMJ* in 1998 randomized into 2 groups: receiving a general form (n=14) or specific form (n=15) for sharing data-sets from the article |
| Age | Not stated |
| Response rate | 60% for specific and 86% for general form |
| Comparisons | NA |
| Outcomes | Number of authors who requested co-authorship as a stipulation for sharing data |
| Methodological limitations | Small sample size |
| Notes |  |

1. **Altman DG et al, 2002, JAMA**

| Discipline | Health: Medicine |
| --- | --- |
| Methods | Descriptive study: postal and e-mail questionnaire survey |
| Data/Sample | Authors of 943 articles submitted to *BMJ* and *Annals of Internal Medicine* from May to August 2001 |
| Age | Not stated |
| Response rate | 75% |
| Comparisons | NA |
| Outcomes | Frequency of recognition of the contribution of a methodologist (biostatistician, epidemiologist or other) as author on the paper |
| Methodological limitations | No details on when the study was performed |
| Notes |  |

1. **Laband DN, 2002, Labour Economics**

| Discipline | Social Sciences: Economy |
| --- | --- |
| Methods | Descriptive study |
| Data/Sample | Authors of feature articles published in 3 leading economics journals (*American Economic Review*, *Journal of Political Economy*, *Quarterly Journal of Economics*) (n=663) and 3 leading agricultural economics journals (*American Journal of Agricultural Economics*, *Review of Agricultural Economics*, *Journal of Agricultural and Resource Economics*) (n=683) |
| Age | NA |
| Response rate | NA |
| Comparisons | Comparison between economics and agricultural economics journals |
| Outcomes | Prevalence of alphabetized coauthorship  Number of individuals thanked in the article in relation to authors of the article |
| Methodological limitations | Prevalence reported as incidence |
| Notes |  |

1. **Mainous III AG et al, 2002, Family Medicine**

| Discipline | Health: Medicine |
| --- | --- |
| Methods | Descriptive study: e-mail questionnaire survey |
| Data/Sample | 578 corresponding authors with functioning e-mail address of original research articles published in 4 medical journals (*Archives of Family Medicine*, *BMJ*, *New Engl J Med*, *American Journal of Psychiatry*)in 1999 |
| Age | Not stated |
| Response rate | 50.5% |
| Comparisons | NA |
| Outcomes | Frequency of concerns about personal and professional relationships enter authorship decisions  Factors affecting authorship if the person does not satisfy authorship criteria  Restricting number of authors as effective policy |
| Methodological limitations | No details on when the study was performed |
| Notes | Authorship criteria according to the International Committee of Medical Journal Editors (ICMJE) |

1. **Mowatt G et al, 2002, JAMA**

| Discipline | Health: Medicine |
| --- | --- |
| Methods | Descriptive study: web-based questionnaire survey |
| Data/Sample | Corresponding authors of 577 reviews published in 1999 *The Cochrane Library* |
| Age | Not stated |
| Response rate | 63% |
| Comparisons | NA |
| Outcomes | Prevalence of ghost and honorary authors  Contributions of authors listed in the byline and the members of the Cochrane editorial team  Methods for assigning authorship |
| Methodological limitations | † |
| Notes | Authorship criteria according to the International Committee of Medical Journal Editors (ICMJE) |

1. **Tarnow E, 2002, Science and Engineering Ethics**

| Discipline | Natural Sciences: Physics |
| --- | --- |
| Methods | Descriptive study: e-mail questionnaire survey |
| Data/Sample | 27,000 members of the APS with functioning e-mail and a PhD from physics, USA |
| Age | Not stated |
| Response rate | 16% |
| Comparisons | NA |
| Outcomes | Probability of inappropriate authorship  Use of APS guidelines in publishing papers  Opinion whether the deserving author could be determined from the byline  Preferences for different authorship definitions |
| Methodological limitations | No details on when the study was performed; low response rate (full answers were available for 13% of the responses); some results presented a frequencies only |
| Notes |  |

1. **Bhandari et al, 2003, Journal of Bone and Joint Surgery**

| Discipline | Health: Medicine |
| --- | --- |
| Methods | Descriptive study: postal questionnaire survey |
| Data/Sample | 23 members of the editorial board of the American Volume of the *J Bone Joint Surg* |
| Age | Not stated |
| Response rate | 91% |
| Comparisons | NA |
| Outcomes | Fraction of responders agreeing on the different bases for authorship order on a scale from strongly agree to strongly disagree |
| Methodological limitations | Small sample size; no details on when the study was performed |
| Notes |  |

1. **Foote MA, 2003, Biotechnology Annual Review**

| Discipline | Health: Medicine (biomedicine) |
| --- | --- |
| Methods | Descriptive study |
| Data/Sample | 14 biomedical journals (*Ann Int Med, Arch Otolaryngol Head neck Surg, CMAJ, JAMA, Lakarridningen, Lancet, Med J Aust, N Engl J Med, Obstet Gynecol, Rev Esp Cardiol, Tidsskr Nor Laegeforen*) |
| Age | NA |
| Response rate | NA |
| Comparisons | NA |
| Outcomes | Definitions of authorship in journals’ instructions for authors |
| Methodological limitations | No explanation on the choice of journals |
| Notes |  |

1. **Hwang SS et al, 2003, Radiology**

| Discipline | Health: Medicine |
| --- | --- |
| Methods | Descriptive study |
| Data/Sample | 1068 original research articles with ≥3 authors published in *Radiology* from 1998 to 2000 (total 6686 authors) |
| Age | NA |
| Response rate | NA |
| Comparisons | NA |
| Outcomes | Authors’ fulfilment of ICMJE criteria |
| Methodological limitations | † |
| Notes |  |

1. **Bates et al, 2004, JAMA**

| Discipline | Health: Medicine |
| --- | --- |
| Methods | Descriptive study |
| Data/Sample | Research articles published in 2002 volumes of 3 major medical journals, *Ann Int Med* (n=72), *BMJ* (n=107) and *JAMA* (n=81) |
| Age | NA |
| Response rate | NA |
| Comparisons | Comparison of undeserved authorship in three journals with different contribution disclosure practices |
| Outcomes | Number of honorary authors according to authors’ contribution as declared in published articles  Number of articles with honorary authorship |
| Methodological limitations | No details on when the study was performed |
| Notes | Undeserved authorship was qualified as that not meeting ICMJE authorship criteria |

1. Bhandari et al, 2004, Epidemiology

| Discipline | Health: Medicine |
| --- | --- |
| Methods | Descriptive study: postal questionnaire survey |
| Data/Sample | 32 chairs of surgery or medicine departments in all 16 Canadian university medical facilities in 2002 |
| Age | Not stated |
| Response rate | 69% |
| Comparisons | NA |
| Outcomes | Fraction of responders assigning the credit for authorship according to the position of the corresponding author in the list of authors (first or last) |
| Methodological limitations | Small sample size |
| Notes | Questionnaire based on Bhandari et al 2003 |

1. **Buchkowsky and Jewesson, 2004, The Annals of Pharmacotherapy**

| Discipline | Health: Medicine |
| --- | --- |
| Methods | Descriptive study |
| Data/Sample | 500 randomly selected clinical trials published in 5 influential medical journals (*Ann Int Med, BMJ, JAMA, Lancet, New Engl J Med*) in 1981-2000 |
| Age | NA |
| Response rate | NA |
| Comparisons | NA |
| Outcomes | Percentage of reported author affiliation with industry  Percentage of industry employees as co-authors |
| Methodological limitations | † |
| Notes |  |

1. **Cohen MB et al, 2004, MedGenMed**

| Discipline | Health: Medicine |
| --- | --- |
| Methods | Descriptive study: e-mail questionnaire survey |
| Data/Sample | 3500 members of the US and Canadian Academy of Pathology (USCAP) |
| Age | Not stated |
| Response rate | 22.5% |
| Comparisons | Comparison with results from previous study on physicists |
| Outcomes | Prevalence of discussion of authorship among researchers when publishing a paper  Appropriateness of stated authorship according to ICMJE, APS or combined definition  Experiences in changes to the authorship list of own articles |
| Methodological limitations | Low response rate, comparison with historical control |
| Notes | The questionnaire was identical to that in Tarnow E, 2002 |

1. **Etemadi A et al, 2004, Saudi Medical Journal**

| Discipline | Health: Medicine |
| --- | --- |
| Methods | Descriptive study: on-site questionnaire survey |
| Data/Sample | 51 editors of Iranian medical journals funded by the Ministry of Health during a conference in 2001 |
| Age | Mean 47.3 (SD8.7), range 34-63 |
| Response rate | 52.9% |
| Comparisons | NA |
| Outcomes | Percent editors agreeing with contributions qualifying for authorship  Knowledge of ICMJE criteria |
| Methodological limitations | † |
| Notes |  |

1. **Marušić M et al, 2004, Science and Engineering Ethics**

| Discipline | Health: Medicine |
| --- | --- |
| Methods | Descriptive study |
| Data/Sample | 475 authors of 114 research articles submitted to the *Croatian Medical Journal* in 1999-2000 |
| Age | NA |
| Response rate | NA |
| Comparisons | NA |
| Outcomes | Percentage of authors satisfying the ICMJE authorship criteria according to their contribution declaration |
| Methodological limitations | No details on when the study was performed |
| Notes |  |

1. **Meyer and McMahon, 2004, Issues in Accounting Education**

| Discipline | Social Sciences: Business |
| --- | --- |
| Methods | Descriptive study: e-mail questionnaire survey |
| Data/Sample | 172 editorial members of 6 accounting journals and 191 young faculty (members of the American Accounting Association New Faculty Consortia), USA |
| Age | 39 in <35 year group; 35 in 35-40, 61 in 41-50, 26 in >50 (15 no response) for the total sample |
| Response rate | 40.6% for editorial board members and 55.5% for young faculty |
| Comparisons | Comparison between experienced and new researchers |
| Outcomes | Perceived appropriateness of co-authorship behavior  Perceived behaviour occurrence for co-authorship issues, including students  Reported actual knowledge of occurrence for co-authorship issues |
| Methodological limitations | Standard deviation not reported for all averages. |
| Notes |  |

1. **Procyshyn RM et al, 2004, Canadian Journal of Psychiatry**

| Discipline | Health: Medicine |
| --- | --- |
| Methods | Descriptive study |
| Data/Sample | 414 research articles about clinical trials on 3 antipsychotic drugs |
| Age | NA |
| Response rate | NA |
| Comparisons | NA |
| Outcomes | Prevalence of authors employed by the industry |
| Methodological limitations | No details on when the study was performed |
| Notes |  |

1. **Szirony TA et al, 2004, Journal of Nursing Education**

| Discipline | Health: Nursing |
| --- | --- |
| Methods | Descriptive study: postal questionnaire survey |
| Data/Sample | 500 randomly selected nursing faculty members from 100 top ranking of graduate degree-granting institutions in USA |
| Age | Not stated |
| Response rate | 77.6% |
| Comparisons | NA |
| Outcomes | Perceptions on ethical issues in nursing research, including authorship  Opinions on deserved authorship, including that related to student’s work |
| Methodological limitations | † |
| Notes |  |

1. **Apgar and Congress, 2005, Journal of Social Work Education**

| Discipline | Social Sciences: Social work |
| --- | --- |
| Methods | Descriptive study: postal questionnaire study |
| Data/Sample | 240 randomly selected members of the Society for Social Work and Research in 2000, USA |
| Age | Not stated |
| Response rate | 67% |
| Comparisons | NA |
| Outcomes | Perceptions on ethical behaviour related to authorship, especially toward student collaborators |
| Methodological limitations | Some results presented as ranks and percentages, without raw numbers |
| Notes |  |

1. **Apgar and Congress, 2005, Journal of Social Work Education**

| Discipline | Social Sciences: Social work |
| --- | --- |
| Methods | Descriptive study: postal questionnaire survey |
| Data/Sample | 301 randomly selected members of the Society for Social Work and Research in 2002, USA |
| Age | Mean 46.1 years |
| Response rate | 62.8% |
| Comparisons | Comparison between genders and teachers vs. researchers |
| Outcomes | Perceptions on ethical behavior related to authorship  Attitudes toward limits on the number of authors on a paper  Attitudes toward using written agreements among collaborators on authorship  Attitudes towards guidelines for authorship |
| Methodological limitations | Some results presented as ranks or means without a measure of variability |
| Notes |  |

1. **Freda and Kearney, 2005, Western Journal of Nursing Research**

| Discipline | Health: Nursing |
| --- | --- |
| Methods | Descriptive study: e-mail questionnaire survey |
| Data/Sample | 137 editors and associate editors of nursing journals |
| Age | Mean 53 years (range 34-69) |
| Response rate | 66% |
| Comparisons | NA |
| Outcomes | Experiences of editors in dealing with ethical issues in authorship |
| Methodological limitations | † |
| Notes |  |

1. **Hilmer and Hilmer, 2005, American Journal of Agricultural Economics**

| Discipline | Social Sciences: Economics |
| --- | --- |
| Methods | Descriptive study |
| Data/Sample | Data set containing the annual salaries of 326 faculty members with teaching/research appointments within twenty top-ranked PhD-granting U.S. agricultural economics departments, and their publications in literature |
| Age | NA |
| Response rate | NA |
| Comparisons | NA |
| Outcomes | Effect of author order on the byline on economists’ salaries |
| Methodological limitations | † |
| Notes |  |

1. **Joubert G, 2005, South African Family** Practice

| Discipline | Health: Multidisciplinary |
| --- | --- |
| Methods | Descriptive study: semi-structured interviews |
| Data/Sample | 36 first authors of 47 papers, from the Faculty of Health Sciences at a university in South Africa |
| Age | Not stated |
| Response rate | NA |
| Comparisons | NA |
| Outcomes | Fraction of authors who had discussion on authorship before publication  Fraction of authors who experienced problems in authorship  Fraction of papers where authors satisfied the authorship criteria |
| Methodological limitations | Small sample size; no details on when the study was performed |
| Notes |  |

1. **Mixon Jr and Sawyer, 2005, Journal of Economics Studies**

| Discipline | Social Sciences: Economics |
| --- | --- |
| Methods | Descriptive study |
| Data/Sample | Articles published in “first-tier” journals (*American Economic Review, Journal of Political Economy, Quarterly Journal of Economics*), and articles published in “second-tier” journals (*Economic Inquiry, Southern Economic Journal*) in 1995-1999 |
| Age | NA |
| Response rate | NA |
| Comparisons | NA |
| Outcomes | The ratio between the number of authors and the whole research team (sum of the number of authors and the number of individuals thanked for assistance in the acknowledgment footnote) |
| Methodological limitations | Results presented only as fractions; distinction between first-tier and second-tier journals not clear |
| Notes |  |

1. **Pignatelli et al, 2005, Journal of Medical Ethics**

| Discipline | Health: Medicine |
| --- | --- |
| Methods | Descriptive study: semi-directive interviews |
| Data/Sample | 39 senior clinical researchers – principal investigators of clinical research programmes in Lyons, France |
| Age | Mean 52 years (SD 8), range 38-69 |
| Response rate | 94.8% (+ 2 co-authors of principal researchers interviewed) |
| Comparisons | NA |
| Outcomes | Awareness and use of ICMJE authorship criteria  Perceptions about ghost and gift authorship |
| Methodological limitations | Small sample size; not clear who rated the quality of interviews and what the rating instrument was |
| Notes |  |

1. **Sandler and Russell, 2005, Ethics and Behavior**

| Discipline | Social Sciences: Psychology |
| --- | --- |
| Methods | Descriptive study: web-based questionnaire survey, followed by a debriefing statement |
| Data/Sample | 604 APA members and student members from the USA who had participated in drafting at least one scholarly article that (a) was a faculty–student collaboration, (b) was psychology based, and (c) resulted in publication of the article |
| Response rate | 100% |
| Age | Mean 49.39 (SD 11.71) |
| Comparisons | Comparison between men and women, faculty and students, and tenured and non-tenured faculty |
| Outcomes | Prevalence of unethical or unfair authorship assignment  Reasons for not reporting unethical behavior |
| Methodological limitations | No details on when the study was performed; response rate not clearly addressed |
| Notes |  |

1. **Birnholtz JP, 2006, Journal of the American Society for Information Science and Technology**

| Discipline | Natural Sciences: Physics |
| --- | --- |
| Methods | Qualitative study (semi-structured 30 to 60 minute interviews) |
| Data/Sample | 32 individuals affiliated in various capacities with ATLAS and Compact Muon Solenoid (CMS), the two major LHC experiments at Large Hadron Collider (LHC) at CERN |
| Age | Not stated |
| Response rate | NA |
| Comparisons | NA |
| Outcomes | Opinions on authorship problems in high energy physics |
| Methodological limitations | Interview protocol not reported; no detailed description of sample; data analysis procedure superficially described; no second researcher to independently confirm the identified themes or contribute to the analysis; findings are poorly structured; no adequate consideration of the relationship between researchers and participants |
| Notes |  |

1. **Burbonniere MC et al, 2006, American Journal of Occupational Therapy**

| Discipline | Health: Multidisciplinary |
| --- | --- |
| Methods | Descriptive study: in-house questionnaire survey |
| Data/Sample | 30 researchers (investigators, associate members, staff) working at the Canadian Child Centre, Ontario, Canada |
| Age | Not stated |
| Response rate | 37% |
| Comparisons | NA |
| Outcomes | Mean response on a 5-point scale about satisfaction with the process used to develop guidelines |
| Methodological limitations | Small sample size; no details on how the sample was constructed or on questionnaire development and its pre-testing |
| Notes |  |

1. **Brown CL et al, 2006, Journal of Marketing Education**

| Discipline | Social Sciences: Marketing |
| --- | --- |
| Methods | Descriptive study |
| Data/Sample | 3702 multiauthored articles with first author from academic institution in 19 leading marketing journals in 1991-2000 (*Decision Sciences, Industrial Marketing Management, International Journal of Research in Marketing, Journal of the Academy of Marketing Science, Journal of Advertising, Journal of Advertising Research, Journal of Business, Journal of Business Research, Journal of Consumer Research, Journal of International Business Studies, Journal of Marketing, Journal of Marketing Education, Journal of Marketing Research, Journal of Personal selling and Sales Management, Journal of Public Policy and marketing, Journal of Retailing, Marketing Letters, Marketing Science, Psychology and Marketing*) |
| Age | NA |
| Response rate | NA |
| Comparisons | NA |
| Outcomes | Alphabetical ordering of authors on the article |
| Methodological limitations | † |
| Notes |  |

1. **Dhaliwal U** et al, 2006, MedGenMed

| Discipline | Health: Medicine |
| --- | --- |
| Methods | Descriptive study: in-house questionnaire survey |
| Data/Sample | 95 faculty in a teaching hospital in India in 2006 |
| Age | 18% in 30-40 age group, 40% in 41-50, 38% in 51-60 and 3% >60 (1% did not disclose age) |
| Response rate | 81.0% |
| Comparisons | NA |
| Outcomes | Awareness of criteria for authorship  Conflicts concerning authorship issues in the research environment |
| Methodological limitations | † |
| Notes |  |

1. **Einav and Yariv, 2006, Journal of Economic Perspectives**

| Discipline | Social Sciences: Multidisciplinary (Economics and Psychology) |
| --- | --- |
| Methods | Descriptive study |
| Data/Sample | 1. Faculty at the top 35 economics departments in the USA, 2. 252 Econometric Society fellows from the sample 1, 3. Nobel Laureates and Clark Winners, 4. Faculty at the top 35 psychology departments in the USA, 5. authors and paper length for all publications at the *American Economic Review*, *Econometrica*, *Journal of Political Economy*, *Quarterly Journal of Economics* and *Review of Economic Studies* from 1980 to 2002 |
| Age | NA |
| Response rate | NA |
| Comparisons | Subgroup analysis of economics and psychology |
| Outcomes | Relationship between the position of the surname in the alphabet and professional success |
| Methodological limitations | † |
| Notes |  |

1. **Laband and Tollison, 2006, Applied Economics**

| Discipline | Multidisciplinary |
| --- | --- |
| Methods | Descriptive study |
| Data/Sample | Co-authored articles in journals from different fields in 1974-1999: 1. general (n=2, *Science, Nature*), 2. medical (n=2, *New Engl J Med, JAMA*), 3. natural sciences (n=10, *Plant Physiology, Bull Geol Soc America, Jnl Wildlife Management, Forest Science, Soil Si Soc Amer Jnl, Jnl Amer Chemical Soc, Physical Rev A, Quarterly Rev Biology, Trans Amer Math Soc*), 4. economics (n=5, *Jnl Finance, Amer Econ Rev, Jnl Marketing, Jnl Accounting Research, Acad Mgmt Jnl*), 5. social sciences (n=5, *Amer Sociological Rev, Amer Polit Sci Rev, Amer Psychologist, Annal Assoc Amer Geogr, Jnlism & Mass Comm Quart*) |
| Age | NA |
| Response rate | NA |
| Comparisons | NA |
| Outcomes | Percent change in fraction of alphabetical authorship |
| Methodological limitations | Results presented only as fractions |
| Notes |  |

1. **Manton and English, 2006, The Delta Pi Epsilon Journal**

| Discipline | Social Sciences: Business |
| --- | --- |
| Methods | Descriptive study: postal questionnaire survey |
| Data/Sample | 392 tenured or tenure-tracked business faculty members of 15 small to mid-sized universities in Texas which do not offer a doctoral degree program in business in the USA |
| Age | Not stated |
| Response rate | 42.1 |
| Comparisons | NA |
| Outcomes | Prevalence of guest authorship practices  Preferred order of listing co-authors in business publications |
| Methodological limitations | No details on when the study was performed |
| Notes |  |

1. **Marušić A et al, 2006, Current Medical Research and Opinion**

| Discipline | Health: Medicine |
| --- | --- |
| Methods | Single-blind randomized study |
| Data/Sample | Authors of 337 manuscripts submitted to a general medical journal (*Croatian Medical Journal*), receiving 3 different formats of contribution declarations for the submitted manuscript |
| Age | Not stated |
| Response rate | 98.2% (1462 authors of 332 manuscripts) |
| Comparisons | Three different formats of contribution declaration: open-ended, categorical and instructional |
| Methodological limitations | No details on pre-testing of contribution declaration forms |
| Outcomes | Prevalence of authors not deserving authorship according to ICMJE criteria  Prevalence of manuscripts with undeserving authorship |
| Notes |  |

1. **Moore and Griffin, 2006, Studies in Educational Evaluation**

| Discipline | Social Sciences: Education |
| --- | --- |
| Methods | Descriptive study: postal questionnaire survey |
| Data/Sample | 196 US and international authors of articles in 5 American Educational Research Association journals (*American Educational Research Journal, Educational Evaluation and Policy Analysis, Educational Researcher, Review of Educational Research, Review of Research in Education*) |
| Age | Not stated |
| Response rate | 30.6% |
| Comparisons | NA |
| Outcomes | Placement of names of authors  Benefit of sole or co-authored publication |
| Methodological limitations | Low response rated; only percentages reported, without raw numbers |
| Notes |  |

1. **Weltzin et al, 2006, Frontiers in Ecology and Environment**

| Discipline | Natural Sciences: Ecology |
| --- | --- |
| Methods | Descriptive study: on-site questionnaire survey during 2004 Annual Meeting of the Ecological Society of America |
| Age | Not stated |
| Data/Sample | 57 participants at the 2004 Annual Meeting of the Ecological Society of America |
| Response rate | Not known |
| Comparisons | NA |
| Outcomes | Opinions about the qualifications for order of authorship |
| Methodological limitations | Small sample size, response rate not known, no details on how the questionnaire was created and whether it was pre-tested |
| Notes |  |

1. **Baerlocher MO et al, 2007, Journal of Investigative Medicine**

| Discipline | Health: Medicine |
| --- | --- |
| Methods | Descriptive study |
| Data/Sample | 109 articles from *JAMA* (2001-2003), 62 from *CMAJ* (20012003), 106 from *BMJ* (1998-2000), and 94 from *Lancet* (1998-2000), respectively (2586 authors) |
| Age | NA |
| Response rate | NA |
| Comparisons | Comparison among journals |
| Outcomes | Authorship position in the byline and declared contributions |
| Methodological limitations | † |
| Notes |  |

1. **Funk CL et al, 2007, Accountability in Research**

| Discipline | Multidisciplinary |
| --- | --- |
| Methods | Before and after survey study – 3 waves of telephone interview followed by on-line questionnaire survey |
| Data/Sample | 426 postdoctoral fellows starting their NIH F32 fellowship training, USA |
| Age | Not stated |
| Response rate | 93% for wave 1, 90% for wave 2 and 79% for wave 3 |
| Comparisons | Comparison before and after training in responsible conduct of research (RCR) |
| Outcomes | Experience with authorship in peer reviewed journals  Awareness of and attention to guidelines for authorship and publication practices  Behavioral judgements about appropriate authorship |
| Methodological limitations | No control group; no raw data presented, only derivations |
| Notes |  |

1. **Geelhoed RJ et al, 2007, Ethics and Behavior**

| Discipline | Social Sciences: Psychology |
| --- | --- |
| Methods | Descriptive study: postal questionnaire study |
| Data/Sample | 300 randomly selected authors of articles published in 5 major clinically related journals in 2001 (*The Counseling Psychologist, Journal of Counseling Psychology, Professional Psychology: Research and Practice, Journal of Consulting and Clinical Psychology, and Journal of Abnormal Psychology*) |
| Age | Mean 42.3 years (SD 10.4), range 26-65 |
| Response rate | 36% |
| Comparisons | Differences between students and staff, tenured and non-tenured staff and those using or not using authorship guidelines |
| Outcomes | Opinions about decision-making process and outcomes in authorship  Predictors of authors’ satisfaction with both the process and outcome of authorship credit decisions |
| Methodological limitations | No details on how the questionnaire was structured and whether it was pre-tested; low response rate; only percentages presented for some variables; score means presented without the possible score range; numbers in some subgroup comparisons very small |
| Notes |  |

1. **Gotsche PC et al, 2007, PLoS Medicine**

| Discipline | Health: Medicine |
| --- | --- |
| Methods | Cohort study of clinical trial protocols and resulting publications |
| Data/Sample | 44 industry-initiated trials approved by the ethics committees in Copenhagen and Frederiksberg in Sweden in 1994-1995 |
| Age | NA |
| Response rate | NA |
| Comparisons | NA |
| Outcomes | Prevalence of ghost authorship |
| Methodological limitations | † |
| Notes |  |

1. **Hren D et al, 2007, Journal of Medical** Ethics

| Discipline | Health: Medicine |
| --- | --- |
| Methods | Combined study design: questionnaire survey and cluster analysis of authorship criteria perceptions and comparison of students with or without instruction |
| Data/Sample | Medical students with (n=152) or without (n=85) prior instructions on ICMJE criteria, graduate students/physicians (n=125) and medical teachers (n=112) from a single medical school |
| Age | Not stated |
| Response rate | 100% for all medical students and physicians, 72% for medical teachers |
| Comparisons | Comparison of 4 study groups in their ratings of contributions qualifying for authorship |
| Outcomes | Reported importance of research contributions for authorship  Clustering of authorship contributions according to reported importance |
| Methodological limitations | No details on how the questionnaire was structured and whether it was pre-tested |
| Notes |  |

1. **Ilakovac V et al, 2007, CMAJ**

| Discipline | Health: Medicine |
| --- | --- |
| Methods | Descriptive study: test-retest prospective study of differences in disclosing contributions to the submitted manuscript |
| Data/Sample | 270 manuscripts submitted to a general medical journal (*Croatian Medical Journal*) |
| Age | Not stated |
| Response rate | 72.0% (919 authors of 201 manuscripts) |
| Comparisons | Contribution disclosures by the corresponding authors of the same manuscripts at two time points of the survey  Contribution disclosures of individual authors provided by corresponding authors and individual authors themselves |
| Outcomes | Test–retest differences between the authors’ self-declarations, expressed in percent as the gross difference rate (GDR) for each article |
| Methodological limitations | † |
| Notes |  |

1. Kurichi and Sonnad, 2007, Surgery

| Discipline | Health: Medicine |
| --- | --- |
| Methods | Descriptive study |
| Data/Sample | 288 chairs of departments of surgery in 87 US medical schools between 1950 to 2004 and their publication record before, during and after chairing the department |
| Age | NA |
| Response rate | NA |
| Comparisons | NA |
| Outcomes | Position on the byline on articles published before, during and after chairing the department |
| Methodological limitations | † |
| Notes |  |

1. **Manton EJ et al, 2007, Journal of Organizational Culture, Communications and Conflict**

| Discipline | Social Sciences: Business |
| --- | --- |
| Methods | Descriptive study: postal questionnaire survey |
| Age | Not stated |
| Data/Sample | Faculty (tenured or tenure–track) of 117 AASCB-accredited colleges of business with fewer than 100 faculty members and without a doctoral program, USA |
| Response rate | 12.8% for colleges (924 faculty) and 20% for faculty of those colleges |
| Comparisons | NA |
| Outcomes | Prevalence of undeserved authorship  Preferred order of listing co-authors |
| Methodological limitations | Small response rate; no details on how the questionnaire was structured and whether it was pre-tested |
| Notes |  |

1. **Peppercorn J et al, 2007, Cancer**

| Discipline | Health: Medicine |
| --- | --- |
| Methods | Descriptive study |
| Data/Sample | All published breast cancer clinical trials (n=140) from 1993, 1998 and 2003 in 10 English-language medical journals (*J Clin Oncol, Ann Oncol, Breast Cancer Res Treat, Cancer, Eur J Cancer, Br J Cancer, J Natl Cancer Inst, Lancet, JAMA, New Engl J Med*) |
| Age | NA |
| Response rate | NA |
| Comparisons | NA |
| Outcomes | Prevalence of authors with industry affiliation |
| Methodological limitations | † |
| Notes |  |

1. **Tryon GS et al, 2007, Training and Education in Professional Psychology**

| Discipline | Social Sciences: Psychology |
| --- | --- |
| Methods | Descriptive study: postal questionnaire survey |
| Data/Sample | 700 randomly selected American Psychological Association of Graduate Students (APAGS) doctoral students in school psychology in 2001, USA |
| Age | Mean 32.29 years (SD 8.17) |
| Response rate | 47% |
| Comparisons | NA |
| Outcomes | Beliefs about credit for authorship of an articles based on dissertation |
| Methodological limitations | No details on how the questionnaire was structured and whether it was pre-tested |
| Notes |  |

1. **Tungaraza T and Poole R, 2007, British Journal of Psychiatry**

| Discipline | Health: Medicine |
| --- | --- |
| Methods | Descriptive study |
| Data/Sample | All published clinical trials on psychiatric drug treatment (n=190) from 3 psychiatry journals in 2000-2004 (*British Journal of Psychiatry, American Journal of Psychiatry, Archives of General Psychiatry*) |
| Age | NA |
| Response rate | NA |
| Comparisons | NA |
| Outcomes | Prevalence of authors with industry affiliation |
| Methodological limitations | Results presented only as frequencies |
| Notes |  |

1. **Wager E, 2007, MedGenMed**

| Discipline | Health: Medicine |
| --- | --- |
| Methods | Descriptive study |
| Data/Sample | 240 biomedical journals randomly selected from the membership list of WAME (n=120) and from Medline (n=120) |
| Age | NA |
| Response rate | 97.5% could be analyzed |
| Comparisons | NA |
| Outcomes | Presence of guidelines about authorship  Adherence to ICMJE authorship criteria |
| Methodological limitations | † |
| Notes |  |

1. **Birnholtz J, 2008, Journal of Electronic Publishing**

| Discipline | Natural Sciences: Physics |
| --- | --- |
| Methods | Qualitative study: semi-structured 30 to 60 minute interviews |
| Data/Sample | 32 individuals affiliated in various capacities with ATLAS and Compact Muon Solenoid (CMS), the two major LHC experiments at Large Hadron Collider (LHC) at CERN |
| Age | Not stated |
| Response rate | NA |
| Comparisons | NA |
| Outcomes | Opinions on authorship problems in high energy physics |
| Methodological limitations | Interview protocol not reported; no detailed description of sample; data analysis procedure superficially described; no second researcher to independently confirm the identified themes or contribute to the analysis; findings are poorly structured; no adequate consideration of the relationship between researchers and participants |
| Notes | The same study as Birnholtz 2006. |

1. **Ivaniš et al, 2008, Journal of General and Internal Medicine**

| Discipline | Health: Medicine |
| --- | --- |
| Methods | Single-blind randomized study of responses to contribution declaration form with binary or ordinal rating scale for contributions |
| Data/Sample | Authors of 181 manuscripts submitted to a general medical journal (*Croatian Medical Journal*) from January to July 2005, receiving two different formats of contribution declarations for the submitted manuscript |
| Age | Not stated |
| Response rate | 95.0% (826 authors of 172 manuscripts) |
| Comparisons | Two different formats of contribution declaration: binary (yes-no) and ordinal (5 point scale, from non to complete) |
| Outcomes | No. of authors deserving authorship according to ICMJE criteria  No. of manuscripts deserving authorship |
| Methodological limitations | † |
| Notes |  |

1. **Lang T, 2008, American Medical Writers’ Association (AMWA) Journal**

| Discipline | Health: Medicine |
| --- | --- |
| Methods | Descriptive study: questionnaire survey |
| Data/Sample | Convenience sample of 16 AMWA members who were experienced medical writers and editors, USA |
| Age | Not stated |
| Response rate | NA |
| Comparisons | NA |
| Outcomes | Opinions on contributions that constitute authorship |
| Methodological limitations | Small sample size, not details on the respondents; no details on how the questionnaire was constructed and whether it was pretested |
| Notes |  |

1. **Louis et al, 2008, Journal of Higher Education**

| Discipline | Health: Biomedicine |
| --- | --- |
| Methods | Qualitative study: grounded theory approach |
| Data/Sample | 32 high profile researchers in 4 research fields (pharmacology, radiation/oncology, neurology and genetics) from 6 doctoral-granting universities in the USA |
| Age | Not stated |
| Response rate | NA |
| Comparisons | NA |
| Outcomes | Personal guidelines for determining authorship (what, how, and why decisions on authorship are made in research groups) |
| Methodological limitations | † |
| Notes |  |

1. **van Praag CM and van Praag BMS, 2008, Journal of Higher Education**

| Discipline | Social Sciences: Economics |
| --- | --- |
| Methods | Descriptive study |
| Data/Sample | All regular articles (n= 2311) published in the period 1997–99 in 11 mainstream economics journals (*American Economic Review, Economica, Economic Journal, European Economic Review, International Economic Review, Journal of Economic Behavior and Organization, Journal of Economic Perspectives, Journal of Economic Theory, Journal of Political Economy, Quarterly Journal of Economics, Review of Economic Studies*) |
| Age | NA |
| Response rate | NA |
| Comparisons | NA |
| Outcomes | Prevalence of alphabetical ordering of authors |
| Methodological limitations | † |
| Notes |  |

1. **Baerlocher MO et al, 2009, Journal of Clinical Epidemiology**

| Discipline | Health: Medicine |
| --- | --- |
| Methods | Before and after study |
| Data/Sample | 1485 original research articles in 5 medical journals with highest impact factors from the first issue of each month in years before and after the introduction of author contribution declaration: *CMAJ* (1995-2003), *The Lancet* (1994-2000), *JAMA* (1994-2003), *BMJ* (1994-2000), *NEJM* (1994-2003) |
| Age | NA |
| Response rate | NA |
| Comparisons | Comparison of articles before and after the introduction of contribution declaration for authors |
| Outcomes | Number of authors per article |
| Methodological limitations | Results presented as percentages; no controls |
| Notes | Authors with group authorship excluded. |

1. **Hu X, 2009, Journal of the American Society for Information Science and Technology**

| Discipline | Multidisciplinary |
| --- | --- |
| Methods | Descriptive study |
| Data/Sample | All articles in the *Journal of Biological Chemistry* in 1999-2008 and 2008 publications in JBC and *Journal of Immunology, Journal of Virology* and *Proceedings of National Academy of Sciences* |
| Age | NA |
| Response rate | NA |
| Comparisons | Comparison among different journals |
| Outcomes | Prevalence of equal first authorship |
| Methodological limitations | † |
| Notes |  |

1. **Maciejeovsky B et al, 2009, Marketing Science**

| Discipline | Social Sciences: Multidisciplinary (Marketing, Economics and Psychology) |
| --- | --- |
| Methods | Descriptive study: on-line questionnaire survey |
| Data/Sample | 1. 247 faculty members and advanced graduate students from economics (n=45), marketing (n=150) and psychology (n=52) in USA/UK  2. 104 faculty members and advanced graduate students from economics (n=21), marketing (n=46) and psychology (n=37) in USA/UK |
| Age | Not stated |
| Response rate | Not stated |
| Comparisons | Comparison in practices among 3 research fields |
| Outcomes | Opinion on contribution credits based on name-ordering conventions |
| Methodological limitations | No data on response rates or selection or origin of the sample |
| Notes |  |

1. **O’Brien J et al, 2009, Canadian Association of Radiologists Journal**

| Discipline | Health: Medicine |
| --- | --- |
| Methods | Descriptive study: e-mail questionnaire survey |
| Data/Sample | 195 corresponding authors of every fourth original research report published in *JAMA* (2001-2003), *BMJ* (1998-2000), *CMAJ* (2001-2003), and *The Lancet* (1998-2000) |
| Age | Not stated |
| Response rate | 65% |
| Comparisons | NA |
| Outcomes | Personal experiences and perception of honorary authorship |
| Methodological limitations | † |
| Notes |  |

1. **Pulido M et al, 2009, Medicina Clinica**

| Discipline | Health: Medicine |
| --- | --- |
| Methods | Descriptive study: postal questionnaire survey |
| Data/Sample | 1010 Spanish authors in sciences and health who publish regularly in international journals and working at tertiary hospital care, research centers or academic institutions linked to hospitals |
| Age | Mean 45.7 years (SD 9.2), range 24-71 |
| Response rate | 51.9% |
| Comparisons | NA |
| Outcomes | Opinions on necessary contributions for authorship credit  Knowledge of the ICMJE authorship criteria |
| Methodological limitations | No details on how the questionnaire was constructed and whether it was pre-tested |
| Notes | In Spanish language |

1. **Rowan-Legg A et al, 2009, Journal of Medical Ethics**

| Discipline | Health: Medicine |
| --- | --- |
| Methods | Descriptive study |
| Data/Sample | Ethics guidelines published in 103 English-language biomedical journals listed in Abridged Index Medicus in 1995 and 2005 |
| Age | NA |
| Response rate | NA |
| Comparisons | Comparison of guidelines in 1995 and 2005 |
| Outcomes | Presence of criteria on authorship |
| Methodological limitations | Journals included in the study were not stated |
| Notes |  |

1. **Samad A et al, 2009, Pakistani Journal of Medical Sciences**

| Discipline | Health: Medicine |
| --- | --- |
| Methods | Descriptive study |
| Data/Sample | Instructions for authors in 40 Pakistani medical and dental journals |
| Age | NA |
| Response rate | NA |
| Comparisons | NA |
| Outcomes | Presence of criteria on authorship |
| Methodological limitations | † |
| Notes |  |

1. **Wager E et al, 2009, Journal of Medical Ethics**

| Discipline | Multidisciplinary |
| --- | --- |
| Methods | Descriptive study: postal questionnaire survey, with e-mail reminder |
| Data/Sample | 612 editors of all medical, health care, life sciences and social science journals published by Blackwell in 2007 |
| Age | Not stated |
| Response rate | 37.7% |
| Comparisons | Subgroup comparison of editors with > 5 years experience vs. others |
| Outcomes | Perceptions on severity and frequency of authorship problems |
| Methodological limitations | Low response rate; no details on how the questionnaire was constructed and whether it was pre-tested, mean ratings reported without measure of variability |
| Notes |  |

1. **Ahmed HS et al, 2010, Learned Publishing**

| Discipline | Health: Multidisciplinary |
| --- | --- |
| Methods | Descriptive study: semistructured questionnaire survey |
| Data/Sample | 100 participants in a bioethics course in Bangladesh |
| Age | Not stated |
| Response rate | 45% |
| Comparisons | Between junior, mid-level and senior researchers |
| Outcomes | Conflicts experienced with authorship  Outcomes of authorship conflicts  Deserved authorship and order of authors |
| Methodological limitations | Low response rate; no details on how the questionnaire was constructed and whether it was pre-tested; no statistical comparison between groups with different research rank. |
| Notes |  |

1. **Akhaue and Lautenbach, 2010, Annals of Epidemiology**

| Discipline | Health: Medicine |
| --- | --- |
| Methods | Descriptive study |
| Data/Sample | Original research articles from 5 high-impact general medical journals published from 2000 to 2009 (*New Eng J Med*=3347; *JAMA*=3012, *Ann Int Med*=1793, *Lancet*=4819, *BMJ*=4945) |
| Age | NA |
| Response rate | NA |
| Comparisons | trends over the years |
| Outcomes | Number of articles with authors given equal credit |
| Methodological limitations | † |
| Notes |  |

1. **Castleden et al, 2010, Journal of Empirical research on Human Research Ethics**

| Discipline | Multidisciplinary |
| --- | --- |
| Methods | Qualitative study |
| Data/Sample | 15 researchers from Canadian universities who are involved in research with Indigenous communities |
| Age | Not stated |
| Response rate | 15 out of 18 contacted |
| Comparisons | NA |
| Outcomes | Current practices and views on granting authorship to Indigenous research participants |
| Methodological limitations | Only 2 of the 15 interviewed researchers were Indigenous; demographic characteristics of the participants poorly described; analytical process not clearly described; quotations not linked to individual respondents, thus it is not clear if all quoted statements came from 2-3 or 10-15 respondents. |
| Notes |  |

1. **Chan et al, 2010, Journal of Real Estate Literature**

| Discipline | Social Sciences: Business |
| --- | --- |
| Methods | Descriptive study |
| Data/Sample | 4490 multi-authored original research articles from 9 academic real estate journals in 1990-2006: *Real Estate Economics* (n=454), *Journal of Real Estate Finance and Economics* (n=569), *Journal of Real Estate Research* (n=583), *Journal of Housing Economics* (n=273)*, Journal of Regional Studies* (n=497), *Journal of Urban Economics* (n=806), *Land Economics* (n=652), *Regional Science and Urban Economics* (n=583), and *International Real Estate Review* (n=73) |
| Age | NA |
| Response rate | NA |
| Comparisons | NA |
| Outcomes | Percent of alphabetically ordered articles |
| Methodological limitations | † |
| Notes |  |

1. **Frandsen and Nicolaisen, 2010, Journal of Informetrics**

| Discipline | Multidisciplinary: Social Science (Economics and Information Science) and Physics |
| --- | --- |
| Methods | Descriptive study |
| Data/Sample | Articles from 27 economic journals, 12 library information science journals and 3 high energy physics journals published from 1978 to 2007 |
| Age | NA |
| Response rate | NA |
| Comparisons | NA |
| Outcomes | Percentage of articles with names listed in alphabetical order  Association between the number of authors per article and share of articles with alphabetical order of authors |
| Methodological limitations | Raw numbers not presented, only percentages for each year, number of articles assessed in each year and journal not available |
| Notes |  |

1. **House and Seeman, 2010, Accountability in Research**

| Discipline | Natural sciences: Chemistry |
| --- | --- |
| Methods | Descriptive study: e-mail questionnaire survey |
| Data/Sample | 3990 faculty from departments of chemistry from 152 colleges and universities granting PhD degrees in 50 states in USA |
| Age | Not stated |
| Response rate | 14% |
| Comparisons | None |
| Outcomes | Deserving contribution for authorship |
| Methodological limitations | No details how the questionnaire was created, on the full content of the questionaire and whether it was pretested, or on when the survey was performed |
| Notes | The same study as Seeman and House 2010 (a) and Seeman and House 2010 (b) . |

1. **Lacasse and Leo, 2010, PLoS Medicine**

| Discipline | Health: Medicine |
| --- | --- |
| Methods | Descriptive study |
| Data/Sample | Public policies of 50 top-rated academic medical centres in the USA |
| Age | NA |
| Response rate | NA |
| Comparisons | NA |
| Outcomes | Statements on ghost writing in policies of academic medical centres |
| Methodological limitations | † |
| Notes |  |

1. **McDonald RJ et al, 2010, Mayo Clinic Proceedings**

| Discipline | Health: Medicine |
| --- | --- |
| Methods | Before and after study |
| Data/Sample | 307190 articles from 16 medical journals (8 with restricting authorship) published between 1986 to 2006 (*Arch Gen Psychiatry, Ann Int Med, Am J Meuroradiology, Am J Roentgen, BMJ, JAMA; Lancet, New Engl J Med, J Clin Invest, J Aller Clin Immunol, J Natl Cancer Inst, Nature Medicine, Circulation, Blood, Radiology, Hepatology*) |
| Age | NA |
| Response rate | NA |
| Comparisons | Subgroup analysis of medical journals before and after the introduction of authorship restriction policies |
| Outcomes | Number of authors per article in relation to journal’s authorship restriction policy (by number limitation or contribution declaration) |
| Methodological limitations | No proper control group, different journals introduced different policies at different times |
| Notes |  |

1. **Morris SE, 2010, Australian Universities Review**

| Discipline | Multidisciplinary |
| --- | --- |
| Methods | Descriptive study |
| Data/Sample | 39 Australian universities |
| Age | NA |
| Response rate | NA |
| Comparisons | NA |
| Outcomes | Compliance of university policies on authorship with the Australian Code for the Responsible conduct |
| Methodological limitations | † |
| Notes |  |

1. **Nastasee SA, 2010, AMWA Journal**

| Discipline | Health: Medicine |
| --- | --- |
| Methods | Descriptive study |
| Data/Sample | 581 articles from 9 medical journals (*Ann Int Med, Archives Int Med, BMJ, CMAJ, Croat Med J, JAMA, Lancet, New Engl J Med, New Zealand Med J*) published in 2000 (n=334) and 2007 (n=247) |
| Age | NA |
| Response rate | NA |
| Comparisons | NA |
| Outcomes | Frequency of medical writers acknowledgment in journal articles |
| Methodological limitations | The choice of journal not fully explained, results presented as frequencies only |
| Notes | All journal, except one, are members of the International Committee of Medical Journal Editors |

1. **Picard M et al, 2010, ERGO – The Journal of the Education Research Group of Adelaide**

| Discipline | Natural Sciences: Agriculture |
| --- | --- |
| Methods | Descriptive study: questionnaire survey |
| Data/Sample | 19 students and 18 supervisors from School of Agriculture, Food and Wine, University of Adelaide, Australia |
| Age | Not stated |
| Response rate | Not stated |
| Comparisons | Students and supervisors |
| Outcomes | Opinions on deserved authorship and project ownership |
| Methodological limitations | The sample not described, not clear how it was generated; no details on how the questionnaire was constructed and whether it was pre-tested |
| Notes |  |

1. **Rose L et al, 2010, Journal of Clinical Oncology**

| Discipline | Health: Medicine |
| --- | --- |
| Methods | Descriptive study |
| Data/Sample | All clinical trials (n=235) published in the *Journal of Clinical Oncology* between January 2006 and June 2007 |
| Age | NA |
| Response rate | NA |
| Comparisons |  |
| Outcomes | Association of declared authorship contributions and financial ties to industry |
| Methodological limitations | † |
| Notes |  |

1. **Seeman and House, 2010, Accountability in Research (a)**

| Discipline | Natural sciences: Chemistry |
| --- | --- |
| Methods | Descriptive study: e-mail questionnaire survey |
| Data/Sample | 3990 faculty from departments of chemistry from 152 colleges and universities granting PhD degrees in 50 states in USA |
| Age | Not stated |
| Response rate | 14% |
| Comparisons | None |
| Outcomes | Deserving contribution for authorship |
| Methodological limitations | No details how the questionnaire was created, on the full content of the questionnaire and whether it was pretested, or on when the survey was performed |
| Notes | The same study as Seeman and House 2010 (b) and House and Seeman 2010. |

1. **Seeman and House, 2010, Accountability in Research (b)**

| Discipline | Natural sciences: Chemistry |
| --- | --- |
| Methods | Descriptive study: e-mail questionnaire survey |
| Data/Sample | 3990 faculty from departments of chemistry from 152 colleges and universities granting PhD degrees in 50 states in USA |
| Age | Not stated |
| Response rate | 14% |
| Comparisons | None |
| Outcomes | Experience of not getting authorship or acknowledgment on a published paper |
| Methodological limitations | No details how the questionnaire was created, on the full content of the questionnaire and whether it was pretested, or on when the survey was performed |
| Notes | The first papers in the series of 3 papers from the same study (other articles: Seeman and House 2010 (a) and House and Seeman 2010). |

1. **Street JM et al, 2010, Social Science & Medicine**

| Discipline | Multidisciplinary: Biomedicine, Clinical Sciences, Social Sciences |
| --- | --- |
| Methods | Qualitative study |
| Data/Sample | 17 staff and doctoral candidates in health research at 2 Australian universities |
| Age | Not stated |
| Response rate | NA |
| Comparisons | NA |
| Outcomes | Opinions on authorship  Opinions on discipline-based authorship practices  Opinions on order of authors and decision making process  Opinions on existing guidelines and responsibilities of authors |
| Methodological limitations | Interviews were not fully transcribed, and some were not even recorded. There were few senior-level researchers, such as professor in biomedical or clinical sciences. There was no attempt to synthesize the findings on a higher level of abstraction. |
| Notes |  |

1. **Walker RL et al, 2010, BMC Medical Education**

| Discipline | Health: Medicine |
| --- | --- |
| Methods | Descriptive study: e-mail questionnaire survey, with e-mail reminders |
| Data/Sample | 687 corresponding authors of original research articles in journals indexed in Thomson Reuters’s 2006 Journal Citation Report category Medicine, General and Internal, published in June 2007 |
| Age | Not stated |
| Response rate | 69.6% total response rate |
| Comparisons | NA |
| Outcomes | Importance of author order on annual performance review and promotion |
| Methodological limitations | No details on how the questionnaire was constructed and whether it was pre-tested |
| Notes | The question on author order was only one of the questions from the survey |

1. **Welfare and Sacket, 2010, Journal of Academic Ethics**

| Discipline | Social sciences. Education sciences |
| --- | --- |
| Methods | Descriptive study: web-based questionnaire survey |
| Data/Sample | 1346 students (n=891) and faculty (n=455) from 80 US universities with graduate studies in education |
| Age | Mean 37.6 years (range 18-98; 99 missing values) |
| Response rate | Could not be calculated because of unknown number of students at institutions |
| Comparisons | Subgroup comparison between faculty and students |
| Outcomes | Perceptions of common and recommended authorship practices  Perceptions of relative importance of contributions |
| Methodological limitations | Not clear if the sample was representative because of unknown response rate |
| Notes |  |

*Abbreviations: NA – not applicable, SD – standard deviation. The size of the sample or data set is the size planned in the study, and from which the response rate was calculated.

†There were no major methodological deficits identified.
