# Supplementary material for: A Systematic Review of Research on the Meaning, Ethics and Practices of Authorship across Scholarly Disciplines
Source: PLoS One. 2011 Sep 8;6(9):e23477. doi: 10.1371/journal.pone.0023477 (PMC3169533; doi:10.1371/journal.pone.0023477)
Supplement: Table S3 — Results of studies addressing the definition of authorship, contributions for deserved authorship and authorship practices. (DOC) [file pone.0023477.s003.doc]

**Table S3.** Definitions of authorship, contributions for deserved authorship and authorship practices*

| **Research field** | | **Reference** | **Study population** | **Outcome** | **Result (prevalence, score, mean, median or P-value)** |
| --- | --- | --- | --- | --- | --- |
| Social sciences | | Spiegel, 1970^11^ | Psychologists in USA | Single contribution that qualifies for authorship:^a^  - choice of statistical method and data analysis  - testing and interpreting tests | 55%  35% |
|  |  |  |  | Preferred solution to multiple authorship:  - respondent’s own  - writer as senior (first) author, other decisions by group  - contribution declaration in journal, then random byline  - writer as senior (first) author decides on authorship | 31%  27%  27%  13% |
| Social sciences | | Bridgewater,^b^ 1981^12^ | Academic psychologists in USA | Agreement of respondents on qualifying contributions for authorship (mostly research design and article writing) | ≥70% |
| Health | | Werley,^b^ 1981^13^ | Nursing professionals in USA | Single contribution that qualifies for authorship:^a^  - drafting the manuscript  - developing and testing data collection instrument  - testing and interpreting tests  - choice of statistical method and data analysis | 53%  46%  42%  33% |
|  |  |  |  | Preferred solution to multiple authorship:  - amount or importance of contribution  - contribution declaration in journal, then random byline  - writer as senior (first) author, other decisions by group  - writer as senior (first) author decides on authorship | 43%  29%  22%  6% |
|  |  |  |  | Opinion of researchers vs. others on deserved authorship for director of service without contribution | 27% vs. 15% |
| Social sciences | | von Glinow, 1982^14^ | Professionals associated with management journals in USA | Opinion of editors vs. editorial review board on collection of data as deserving authorship | 65% vs. 44% |
| Health | | Waltz,^b^ 1985^16^ | Health professionals in nursing in USA | Authorship should not be deserved for:  - making appointments for study subjects^c^  - typing the manuscript  - only contributing research idea | 92%  89%  89% |
| Social sciences | | van der Kloot, 1991^18^ | Social psychologists and psychometricians in The Netherlands | Scores on a continuum scale of deserving authorship (0=no contribution at all, 100=all contributions by one person):  - writing the article  - designing the study  - leadership of research project  - data analysis  - data collection | 26  21  20  18  15 |
| Health | | Diguisto, 1994^23^ | University research staff in Australia | Means of maximum points on 0-6 scale that should be awarded to contributions for authorship:  - thinking up the idea or drafting a manuscript  - preparing grant application; directly supervising/coordinating; creative input; or planning and carrying out statistical analysis | 5  4 |
| Social sciences | | Floyd, 1994^24^ | Authors of articles published in management journals | Scores on importance of contributions (mean±SD): ^d^  - providing support  - data analysis  - having core idea  - doing the writing  Scores on factors for inclusion and ordering of authors (mean±SD):^d^  - prestige  - arbitrary  - contribution | 1.35±0.52  2.56±0.76  3.11±0.63  4.34±0.65  1.16±0.51  1.82±0.63  4.83±0.55 |
| Health | | Goodman, 1994^25^ | First authors or research articles in general medical journal | Prevalence (95% confidence interval) of authors who satisfied ICMJE authorship criteria | 64% (52% – 75%) |
|  |  |  |  | Median number (range) of contributions to first and last authors | 10 (range 5 – 13) vs. 4 (2 – 6) |
| Health | | Shapiro, 1994^26^ | First authors from USA of research articles in general medical journal | Most frequent (>50%) contributions by all authors as reported by first author:  - provision of resources  - writing and revision  - data collection  - analysis and interpretation of data | 68%  57%  54%  52% |
|  |  |  |  | Most frequent contribution for authors with single contributions:  - provision of resources | 58% |
| Social sciences | | Wagner, 1994^27^ | Single, first or second author in a psychology journal | Mean percentage of importance for authorship:^e^  - writing the paper  - having the idea  - planning the design | 19.3±11.6  16.4±12.0  10.5±7.5 |
| Multidisciplinary | | Eastwood, 1996^29^ | Postdoctoral fellows at a university | Sufficient contribution for authorship (>50% positive responses):  - design of the study  - experiments and collection of data  - analysis and interpretation of data  - writing first draft  - developing testable hypothesis for the study | 91.7%  85.8%  84.6%  69.1%  65.4% |
| Health | | Bhopal, 1997^31^ | Staff from university medical school in UK | Reported agreement with ICMJE authorship criterion:  - conception/design, analysis and interpretation  - drafting or critical revision of article  - final approval  - all 3 criteria should be met | 82%  83%  85%  30% |
|  |  |  |  | Contributions that alone merit authorship (>50% respondents:  - providing statistical advice on ongoing basis  - designing the study  - conceiving research idea | 92%  88%  71% |
|  |  |  |  | Reported attitude on authorship criteria:  - there should be one  - aware of any  - have used any  - heard of ICMJE  - aware of ICMJE criteria | 76%  49%  35%  50%  24% |
| Social sciences | | Hamilton, 1997^33^ | Business and non-business university faculty in USA | Deserving joint authorship for single contribution:  - major editorial revision/data collection/ data manipulation/data interpretation/conceptual contribution/ 25% to 50% of research  - less than 25% research | 75%/81%/85%/90%/ 80%90%  68% |
|  |  |  |  | Authorship for only final preparation and submission:  business vs. non-business | 44% vs. 21% |
| Social sciences | | Netting, 1997^34^ | University faculty and student in focus groups in USA | Emerging themes in authorship:  1) professional socialization and acculturation (first experiences, disciplinary norms, value of co-authoring)  2) professional development and growth (perceived changes in authoring issues)  3) negotiation/renegotiation (ownership, level of contribution, relationship)  4) professional responsibility (to others and profession for accuracy and accountability of work) |  |
| Health | | Almeida, 1998^35^ | Mental health professionals (physicians and non-physicians) in Brazil | Opinions of physicians vs. non-physicians on contributions valid for granting authorship, (P≤0.05):  - study conception  - data collection  - entering data into computer  - providing patients for the study  - approval of publication | 68% vs. 96%  42% vs. 71%  0% vs. 25%  0% vs. 21%  26% vs. 67% |
| Health | | Butler, 1998^36^ | Nurses expected to publish research in Canada | Agreement in modal responses among nurses of different professional status for:  - volunteer or paid member below doctoral level should be author if contribution similar to paid or doctoral level  - nursing staff helping with collecting data and clinical observations acknowledged in footnote | 85%  80% |
| Health | | Hoen, 1998^39^ | Authors of articles published in national general medical journal in The Netherlands | Authors self-reported fulfilment of ICMJE criteria | 63.3% |
|  |  |  |  | Authors not aware of ICMJE criteria | 59.8% |
|  |  |  |  | Discrepancy between own and co-author declaration on contribution (mode, 5^th^ and 95^th^ percentile) | 2 (4 – 8) |
| Health | | White, 1998^41^ | First authors of papers on nursing research from USA | Having knowledge of:  - agency or institution authorship guidelines  - APA authorship ethical guidelines | 15%  60% |
|  |  |  |  | Reported contributions to different aspects of manuscript:^f^  - at least 1 aspect  - 2 or more aspects | 97%  85% |
|  |  |  |  | Prevalence of articles with all authors qualifying for authorship | 59% |
| Multidisciplinary | | Rose, 1999^45^ | Ethics statements from scientific professional organizations in USA | Prevalence of statements on authorship in ethics codes:  - nonspecific (being truthful or accurate, giving proper credit)  - criteria for authorship  - honorary of gift authorship  - including all who merit authorship  - order of authors  - taking responsibility for work and publishing | 39% – 56%  17%  9%  14%  4%  10% |
| Natural sciences | | Tarnow, 1999^46^ | Postdoctoral fellows in physics in USA | Knowledge of association authorship guidelines | 26% |
|  |  |  |  | Authorship criteria never discussed with supervisor | 75% |
|  |  |  |  | Criteria for designating postdocs or others as authors not clearly agreed upon | 61% or 70% |
| Health | | Yank, 1999^47^ | Articles in general medical journal | Contribution declared for authors and in acknowledgment lists:  - wrote paper  - analyzed or interpreted data  - collected data  - performed clinical analysis or management  - performed statistical analysis  - advised on design or analysis  - managed data | 62% vs. 5%  32% vs. 3%  22% vs. 20%  15% vs. 5%  9% vs. 4%  9% vs. 11%  6% vs. 7% |
| Social sciences | | Bartle, 2000^48^ | Faculty and students from psychology departments in USA | Most important contributions for authorship on 1-7 scale (mean±SD):^g^  - writing  - idea generation/design  - data analysis/supervision  - status/seniority  - data collection | 5.9±1.0  5.5±1.4  4.0±1.2  2.9±1.7  2.8±1.3 |
|  |  |  |  | Task that should be most important for authorship (weighted score of average sum of ranks):  1. generating research topic  2. developing research design  3. developing research hypothesis  4. writing first draft  5. writing final draft | 0.81  1.26  1.28  1.32  1.60 |
|  |  |  |  | Opinion of students vs. faculty APA ethical guidelines:^g^  - knowledge of (P<0.05)  - too vague to be useful in practice | 5±1 vs. 6±1  4±1 vs. 4±2 |
| Social sciences | | Hart, 2000^49^ | Co-authors of papers in library science | Importance of research task (mean±SD, scale 1-10):  - writing paper  - collecting data  - analyzing data  - designing study  - revising paper  - reviewing literature  - having original idea | 8.5±1.8  8.5±1.8  8.3±2.0  8.1±2.0  8.1±2.0  7.5±2.3  7.4±2.7 |
| Health | | Price, 2000^50^ | Faculty from institutions granting graduate degrees in nursing in USA | Criterion most important for authorship (>40% response):  - helped writing manuscript  - helped data analysis and interpretation  - helped design study | 48%  45%  45% |
|  |  |  |  | Opinion on no. criteria needed for authorship:  - only one  - 4 or more of 8 offered | 31%  38% |
|  |  |  |  | Role of journals in authorship issues:  - should require signed forms for contribution declaration  - list study parts for which authors are responsible  - provide acknowledgement for minor contributions  - restrict number of authors | 46%  22%  69%  22% |
| Health | | Phillips,^h^ 2001^52^ | Authors of articles in large and small medical journals | Acknowledgement of medical writing assistance as authorship | 16% |
| Health | | Altman, 2002^54^ | Authors of articles in general medical journals | Recognition ofa methodologist as author:  - biostatistician, epidemiologist, other | 65%, 88%, 82% |
| Social sciences | | Laband, 2002^55^ | Authors in economic and agricultural economics journals | Fraction of production team given authorship rights in economics vs. agricultural economics (P<0.01) | 29% vs. 68% |
| Health | | Mowatt, 2002^57^ | Corresponding authors of Cochrane systematic reviews | Contributions (≥70% responses) of authors vs. Cochrane editorial team:  - assessing study quality  - interpreting data  - abstracting data from studies  - screening search results  - revising manuscript  - conception and design | 83% vs. 21%  82% vs. 30%  77% vs. 17%  76% vs. 32%  73% vs. 44%  70% vs. 45% |
| Natural sciences | | Tarnow, 2002^58^ | Members of American Physical Society (APS) | APS authorship guidelines not used in publishing experiences | 92% |
|  |  |  |  | Preference of authorship guideline:  - APS  - ICMJE  - different or no requirements | 64%  15%  12% |
|  |  |  |  | No. of reports for articles with >20 authors:^i^  - undeserved authors per APS guideline – 0-20% vs. 80-100%  - undeserved authors per APS guideline – 0-20% vs. 80-100% | ~98 vs. ~17  ~41 vs. ~78 |
| Health | | Foote, 2003^60^ | Biomedical journals | No. journals without definition of authorship in guidelines | 5 out of 14 |
| Health | | Cohen, 2004^65^ | Members of US and Canadian Academy of Pathology (USCAP) | APS authorship guidelines not used in publishing experiences | 89% |
|  |  |  |  | Expressed preference of authorship guideline:  - newly proposed guideline  - APS  - ICMJE  - different or no requirements | 40%  16%  24%  18% |
| Health | | Etemadi, 2004^66^ | Editors of medical journals in Iran | Criteria for authorship (>50% response) – agreement with/regarded as necessary/thought were from ICMJE:  - writing first draft  - designing study  - active supervision of data collection  - conception of original idea | 89%/36%/48%  82%/30%/48%  74%/22%/30%  63%/26%/26% |
|  |  |  |  | Treatment of patients is eligible for authorship | 23% |
| Health | | Pignatelli, 2005^77^ | Senior clinical researchers in France | Practices in authorship:  - use of any vs. ICMJE criteria for choosing co-authors  - there was intervention of co-authors  - knowledge of any authorship criteria  - knowledge of ICMJE or ICMJE authorship criteria  - use of ICMJE criteria | 89% vs. 14%  79%  51%  51% or 5%  41% |
|  |  |  |  | Agreement with ICMJE criteria:  - conception/design; data acquisition, analysis and interpretation  - writing or revising  - final approval of the version to be published  - all three must be met | 97%  80%  62%  18% |
| Natural sciences | | Birnholtz, 2006^79^ | Researchers in high energy physics (HEP) | Themes in authorship in physics:  - balancing attribution of credit to large group with individual need for recognition  - difference between infrastructural/discovery –oriented contributions to research endeavours  - pragmatic strategies for survival given authorship practices in HEP |  |
| Health | | Burbonniere, 2006^80^ | Researchers at a clinical centre in Canada | Satisfaction with use of in-house authorship guideline (mean, range; scale 1-5) | 4.1 (3 – 5) |
| Health | | Dhaliwal, 2006^82^ | Faculty in teaching hospital in India | Acceptable criteria for authorship (>50% positive response):  - study conception and design  - drafting and revising manuscript  - acquiring data for study  - analysis and interpretation of data  - critical review of the proposal | 100%  88%  78%  71%  53% |
| Multidisciplinary | | Funk, 2007^90^ | NIH postdoctoral fellows in USA | Awareness and use of authorship guidelines at 3 time points after training on RCR | 49%/51%/57% |
| Social sciences | | Geelhoed, 2007^91^ | Authors of research articles in clinical psychology journals | Most common opinions on authorship decision process:  - authorship discussed at idea generation  - formal or informal standards used for decisions  - first authors as deciders on co-authorship | 43%  41%  87% |
|  |  |  |  | Opinion on factors other than effort and contribution affecting authorship decisions:  - taking project leadership  - loyalty or obligation  - power differentials  - publish or perish pressures | 40.4%  32.1%  17.4%  16.5% |
|  |  |  |  | Tenured vs. untenured faculty opinion on factors other than effort or contribution affecting authorship (P<0.05):^j^  - power differentials  - loyalty or obligation  - satisfaction with decision process (mean±SD)  - perceived power relative to others (mean±SD) | 6.3% vs. 30.4%  20.8% vs. 43.5%  4.6±0.9 vs. 4.0±1.0  3.5±1.1 vs. 2.8±1.1 |
|  |  |  |  | Opinion on use vs. no use of guidelines influencing satisfactions with authorship decisions (P<0.05):  - outcome satisfaction  - process satisfaction | 4.8±0.6 vs. 4.3±1.1  4.7±0.6 vs. 4.2±1.1 |
| Health | | Ilakovac, 2007^94^ | Authors of research articles in general medical journal | Reliability of contribution declaration form for corresponding authors (gross difference rate (95% CI), highest – lowest reliability):  - conception/design – provision of study materials | 4.0 (2.0-7.7) – 22.9 (17.6-29.2) |
|  |  |  |  | Percent non- corresponding authors satisfying ICMJE criteria when contributions self reported vs. reported by corresponding author (P<0.001) | 40.5% vs. 28.4% |
| Health | Wager, 2007^100^ | | Guidelines for authors in medical journals | Presence of authorship guidance  Reference to current ICMJE version among those with ICMJE reference | 59%  65% |
| Natural sciences | Birnholtz,^k^ 2008^101^ | | Researchers in high energy physics (HEP) | Emerging themes in HEP authorship:  - individual remains the unit of organization  - larger collaboration means larger range of contributions |  |
| Health | | Ivaniš, 2008^102^ | Authors of research articles in general medical journal | Prevalence of authors satisfying ICMJE criteria when declaring contributions in a binary vs. ordinal rating scale (P<0.05) | 39% vs. 88% |
| Health | | Lang, 2008^103^ | Experienced medical writers from USA | No. respondents out of 16 with opinion that authorship is deserved when medical writer:  - searches and selects literature, chose information for manuscript  - find published cases and write case report  - process raw data and write results and discussion section of manuscript | 14  12  14 |
| Health | | Louis, 2008^104^ | High profile researchers in biomedicine in USA | Identified guiding factors for authorship decisions:  1) fairness  2) reciprocity  3) sponsorship |  |
| Health | | Baerloccher, 2009^106^ | Original research articles in general medical journals | No decrease in number of authors after introduction of contribution disclosure | P=0.984 |
| Health | | Pulido, 2009^110^ | Spanish authors in health who publish in international journals | Most important contributions for any author vs. first author:  - idea, design, hypothesis, planning, conception  - editing manuscript  - technical/experimental work, collection of data  - statistical analysis  - interpretation, discussion, conclusions  - revision, correction, criticism of work | 85%vs. 62%  83%vs. 71%  68%vs. 39%  58%vs. 27%  40%vs. 24%  37%vs. 12% |
|  |  |  |  | Knowledge of ICMJE criteria | 38% |
| Health | | Rowan-Legg, 2009^111^ | Guidelines published in biomedical journals | Prevalence (%, 95% CI) of journals with authorship addressed in guidelines in 2005 vs. 1995 | 72% (63-81%) vs. 40% (31-50%) |
| Health | | Samad, 2009^112^ | Pakistani medical and dental journals | Prevalence of journals with no guidance on authorship | 32.4% |
| Multidisciplinary | | Castleden, 2010^116^ | Researchers involved in research with Indigenous communities in Canada | Identified collective/community authorship as emerging but inconsistent practice of acknowledging |  |
| Natural sciences | | House,^l^ 2010^119^ | Faculty from departments of chemistry in USA | Factors explaining deserved authorship (% variance):  1. responsibility/accountability  2. tangible contributions  3. core intellectual contributions | 25.7%  13.3%  9.3% |
|  |  |  |  | Factors explaining influences on authorship (% variance):  1. graduate school education  2. institutions or outside sources  3. personal values | 31.2%  19.2%  13.9% |
| Health | | McDonald, 2010^121^ | Articles from medical journals | Influence of authorship restriction policies on number of authors from 1986 to 2006 (ANOVA):  - no effect of restriction policy implementation  - no effect of restriction type: numerical restriction or contribution declaration | P=0.52  P=0.61 and P=0.81 |
| Multidisciplinary | | Morris, 2010^122^ | All (n=39) Australian universities | No. universities with authorship policy and policy rating:^m^  1. full compliance  2. significant compliance  3. link to national code provided  4. no policy found | 12  17  4  3 |
| Natural sciences | | Seeman, 2010^126^ | Faculty from departments of chemistry in USA | Prevalence of “acknowledgment” as solution to scenario where person makes suggestion that permits successful competition of research, regardless of who person was or where suggestion made | 51.2% |
|  |  |  |  | Situational differences:  - academics from polymer chemistry or those with non-USA PhD or having 11-30 publications more likely to give less credit  - those who reported having problem with professor or teacher more likely to give nothing and less likely to give co-authorship | P=0.05 and P=0.029  P=0.015 |
| Multidisciplinary | | Street, 2010^128^ | Staff and doctoral candidates in health research at Australian universities | Emerging themes in authorship:  1. work as main qualification  2. denial of deserved authorship rare  3. power structure in ascribing authorship more important for health research then social sciences  4. conventions on authorship order: first author most valuable for all, last author valuable for health researchers  5. ad hoc as common decision process, guidelines rarely used, no instruction received  6. trust, power and responsibility important |  |

*Abbreviations: CI, confidence interval; SD, standard deviation, ICMJE, International Committee of Medical Journal Editors; APA, American Psychological Association, APS, American Physical Society; NIH, National Institutes of Health, USA; RCR, responsible conduct of research.

^a^Only contributions chosen by ≥30% respondents.

^b^Partial or full replication or modification of questionnaire by Spiegel and Keith Spiegel, 1970.^11^

^c^There was >82% agreement on this item with other health professionals from the study (dentistry, medicine, pharmacy, social work).

^d^Results for three matrices are presented as means ±standard deviation of scores, but the max. score was not available from the article.

^e^Expressed as percentage (±standard deviation) of a total task of producing the research project (only the contributions with >10% relative value); there were no differences among authors with different positions on byline (P>0.72); writing the report and having the idea were significantly more important than planning the design, P>0.05).

^f^Inital conception, provision of resources, study design, data collection, data analysis and interpretation, writing first draft, revision of draft.

^g^On a scale from 1 (strongly disagree) to 7 (strongly agree).

^h^Sub-analysis of data from Flanagin et al.^38^

^i^The results were presented in a graph and the numbers in the table are approximations.

^j^There was no information on the scale range for presented mean scores.

^k^The same study as Birnholtz, 2006.^79^

^l^House and Seeman^119^ and Seeman and House^126^ present results from the same study, together with Seeman and House^127^.

^m^Rating of policies: 1. present online, includes authorship criteria (as defined by national code), statement on determining authorship order; 2. present online, includes authorship criteria; 3. not present online but link to national code; 4. not found online after extensive search.
