# Supplementary material for: A Systematic Review of Research on the Meaning, Ethics and Practices of Authorship across Scholarly Disciplines
Source: PLoS One. 2011 Sep 8;6(9):e23477. doi: 10.1371/journal.pone.0023477 (PMC3169533; doi:10.1371/journal.pone.0023477)
Supplement: Table S4 — Results of studies addressing the order of authors on the byline. (DOC) [file pone.0023477.s004.doc]

**Table S4.** Order of authors on the byline*

| **Research field** | **Reference** | **Study population** | **Outcome** | **Result (prevalence/percentage, score, number, P-value, odds ratio, coefficient)** |
| --- | --- | --- | --- | --- |
| Multidisciplinary | Zuckerman, 1967^8^ | Nobel laureates in USA and matched scientists | 1^st^ authorship on a paper:  - laureates vs. others at 20years of age  - laureates vs. others at 40 years of age | a half vs. a third  26% vs. 56% |
| Multidisciplinary | Zuckerman,^a^ 1968^9^ | Nobel laureates in USA and matched scientists | Ratio observed/expected frequency of papers with 6 or more authors and name order pattern for laureates vs. others:  - alphabetical  - alphabetical + last out of sequence | 270.4 vs. 133.3  3.6 vs. 0 |
| Health | Over, 1970^10^ | Articles published in *J Physiol* 1961-1964 | Percent authors with A-E vs. P-Z surnames in a journal with alphabetical author listing | %P-Z less by 12.1% |
| Social sciences | Spiegel, 1970^11^ | Psychologists in USA | Preferred method for authorship order when contributions are equal:  - tossing a coin  - alphabetical order | 60%  33% |
|  |  |  | Prefer pre-study agreement as authorship policy | 83% |
|  |  |  | Authorship order should be determined only by importance of contribution | 82% |
| Health | Werley,^b^ 1981^13^ | Nursing professionals in USA | Preferred method for authorship order when contributions are equal:  - alphabetical order  - according to journal policy  - tossing a coin | 42%  34%  20% |
|  |  |  | Prefer pre-study agreement as authorship policy | 77% |
|  |  |  | Authorship order should be determined only by importance of contribution | 80% |
|  |  |  | Opinion of researchers vs. others that credit should be decided after study | 19% vs. 9% |
| Social sciences | von Glinow, 1982^14^ | Professionals associated with management journals in USA | Preferred method for ordering authors:  - alphabetical ordering of authors  - order based on rank or prestige | 52%  24% |
| Social sciences | Over, 1982^15^ | Articles in psychology journals | No. of articles with alphabetical ordering of authors on articles with 3 authors in 1949, -59, -69, -79 | 14%, 21%, 19%, 16% vs. expected 17% |
| Health | Waltz,^b^ 1985^16^ | Health professionals in nursing in USA | Preferred method for authorship order when contributions are equal:  - first authorship for designing the project | 85% |
|  |  |  | Prefer pre-study agreement as authorship policy | 85% |
|  |  |  | Authorship order should be determined only by importance of contribution | 82% |
| Health | Gay,^b^ 1987^17^ | Educators in nursing USA | Authorship order should be based on contributions | 89% |
|  |  |  | Dean should not get even a footnote for reading manuscript | 71% |
| Social sciences | McCarl, 1993^21^ | Citations in 5 journals on agricultural economics | Lower chance of having a citation as first-author citations when surname on “Z” compared with surname on “A” | 11.9% |
|  |  |  | Alphabetic order maintained:  - for 2-autor articles (vs. 50% predicted)  - for 3-author articles (vs. 17% predicted) | 63%  32% |
| Health | Shulkin, 1993^22^ | Articles by chairs of department of medicine in USA | More last-authorship papers for short-term chairs (<10 years) than for long-term chairs | P<0.01 |
| Health | Shapiro, 1994^26^ | First authors from USA of research articles in general medical journal | Most frequent contributions of first vs. last author:^c^  - initial conception  - design  - provision of resources  - data collection  - analysis and interpretation of data  - writing and revision | 90% vs. 64%  97% vs. 61%  72% vs. 85%  89% vs. 34%  98% vs. 61%  100% vs. 80% |
|  |  |  | Total No. contributions of first vs. last author:^c^  - 0 or 1  - 2 or 3  - 4, 5 or 6 | 0% vs. 10%  3% vs. 29%  97% vs. 61% |
|  |  |  | Significantly more last authors of clinical vs. basic science articles contributed to <2 tasks | 16% vs. 3% |
| Social sciences | Wagner, 1994^27^ | Single, first or second author in a psychology journal | Mean percent contribution for position:^d^  - first to fourth author  - first to fifth author  - first to sixth author | 50%/25%/11%/8%  56%/13%/11%/ 8%/6%  59%/16%/9%/4%/5%/2% |
| Health | Davies, 1996^28^ | Chairs of pediatric departments and deans of medical faculties in Canada | Mean (±standard deviation) score of first author contribution when:^e^  - all collaborators listed  - principal authors named + group acronym  - group acronym + author list in footnote | 9.6±0.8  9.7±0.7  7.4±2.7 |
| Health | Slone, 1996^30^ | First authors from USA on papers from a radiology journal | Reported contributions of first authors vs. 5^th^-10^th^ author:^f^  - research and design  - data collection  - data analysis  - manuscript preparation  - contributed to 3 or 4 categories | 98% vs. 34%  97% vs. 41%  99% vs. 31%  100% vs. 45%  99% vs. 25% |
|  |  |  | Mean (± standard deviation) number of contributions for author position:  - first  - second  - third  - fourth  - fifth to tenth | 63±17  20±12  10±7  5±6  5±5 |
| Health | Butler, 1998^36^ | Nurses in Canada, expected to publish research | Agreement of modal responses among nurses of different professional status that order of authorship should be based on contributions, not status | 80% |
| Health | Drenth, 1998^37^ | Authors of articles in general medical journal 1975-1995 | Prevalence of senior level authors as last authors in 1975 vs. 1995 (P<0.001) | 20.4% vs. 29.0% |
| Health | White, 1998^41^ | First authors from USA on papers on nursing research | Knowledge of agency or institution guidelines for authorship sequencing | 5% |
| Multidisciplinary | Engers, 1999^43^ | Articles from journals on law, economics, social sciences, natural sciences or medicine | Prevalence of alphabetical ordering of authors:  - economic journals (n=2)  - law journal  - social science journals (n=2)  - chemistry journal  - medical journal | 82% and 85%  84%  39% and 43%  53%  6% |
| Health | Yank, 1999^47^ | Articles in general medical journal | Mean number of contributions for byline position (P<0.01): 1^st^ vs. 2^nd^vs. 3^rd^ vs. last | 3.23 vs. 2.51 vs. 2.20 vs. 2.51 |
|  |  |  | First author vs. other contributors reported coordination of study | 43% vs. 20% |
|  |  |  | First and last authors vs. other contributors:  - written manuscript  - designed study  - analyzed data | 84%, 80% vs. 62%  58%, 52% vs. 40%  51%, 32% vs. 32% |
| Social sciences | Hart, 2000^49^ | Co-authors of papers in library science | Most prevalent method of ordering authors:  - in order of significant contribution  - alphabetic listing to indicate equal contribution  - alphabetic, no intent to indicate equal contribution  - other | 46.9%  15.3%  9.2%  28.6% |
| Health | Chambers, 2001^51^ | Articles in general medical journal | First authorship for name starting with letter more common than 3^rd^ of 4^th^ for:  - A, E, F, G, H, I, J, L, M out of first half of alphabet  - P, Y out of second half of alphabet | up to 50%  up to 80% |
| Social sciences | Laband, 2002^55^ | Authors of articles in economic and agricultural economics journals | Prevalence of alphabetized co-authorship in economic vs. agricultural economics journals (P<0.01) | 89% vs. 44% |
| Health | Mowatt, 2002^57^ | Corresponding authors of Cochrane systematic reviews | Reported practices in deciding on authors’ order:  - according to contributions  - alphabetically by surname  - other methods (seniority, senior author last, internal discussion, stage when getting involved) | 76%  2%  22% |
| Natural sciences | Tarnow, 2002^58^ | Members of American Physical Society (APS) | Probability of change after initial authorship list is determined:  - for decrease  - for increase | 4%  12% |
|  |  |  | Peer cannot tell from the list of authors who made greatest contribution | 46% |
| Health | Bhandari, 2003^59^ | Editorial board members of medical journal in USA | Agreement that authorship order should be based on:  - amount of work done  - alphabetical order  - author seniority  - random order  - writing manuscript  - obtaining financial support | 90%  0%  20%  0%  86%  14% |
|  |  |  | Significant change in perception of author’s role when last author vs. first designated as corresponding:  - for first author to critically revise manuscript  - for last author to contribute to study conception and design/critical revision/supervision | 29% decrease  42%/29%/33% increase |
| Health | Bhandari, 2004^63^ | Chairs of surgery or medicine departments in Canada | Change in assignment of authorship credit to first author when last vs. first author is corresponding author:  - study conception and design  - analysis and interpretation  - manuscript critical revision  - statistical analysis  -obtaining funding  - administrative support  - supervision  - major prestigious position | 73% vs. 82%  82% vs. 100%  55% vs. 68%  14% vs. 27%  32% vs. 59%  0% vs. 27%  5% vs. 23%  77% vs. 91% |
|  |  |  | Change in assignment of authorship credit to last author when first vs. last author is corresponding author:  - study conception and design  - analysis and interpretation  - manuscript critical revision  - statistical analysis  - obtaining funding  - administrative support  - supervision  - major prestigious position | 36% vs. 77%  32% vs. 59%  64% vs. 77%  5% vs. 0%  27% vs. 68%  41% vs. 77%  46% vs. 86%  9% vs. 23% |
| Health | Cohen, 2004^65^ | Members of US and Canadian Academy of Pathology (USCAP) | Probability of change after initial authorship list is determined:  - for decrease  - for increase | 3%  18% |
|  |  |  | Peer cannot tell from the list of authors who made greatest contribution | 38% |
| Social sciences | Meyer, 2004^68^ | Editorial members of accounting journals and young accounting faculty members in USA | Perceived behaviour appropriateness/perceived occurrence/actual knowledge of occurrence of co-authorship issues:^g^  - alphabetical ordering of names rather than according to contribution  - ordering reflects prestige or rank rather than contribution | 6.8/7.8/3.4  2.7/4.4/1.7 |
| Social sciences | Apgar, 2005^72^ | Members of Society for Social Work and Research in USA | Rank order (scale 1-5) of views on research tasks in decisions on authorship order:  - writing manuscript  - quantity/quality of writing  - quantity/quality of work  - ownership of data  - expertise  - relationship between collaborators  - payment for services | 4.41  4.20/3.97  3.91/3.64  3.19  2.73  2.22  1.92 |
|  |  |  | Opinions on authorship order:  - using a ranking points system in deciding on authorship order is valuable  - previous authors were less likely to think points system is worthwhile  - there should be no limits on number of authors  - use of written agreements on authorship before research onset | 37.4%  OR=0.32 (95%CI 0.13 – 0.77  87.5%  60.1% |
| Social sciences | Hilmer, 2005^74^ | Faculty members of agricultural economics departments in USA and their publications | Prevalence of alphabetical authorship in co-authored vs. multi-authored articles | 63% vs. 41% |
|  |  |  | Estimated annual salary return to an additional article (P<0.05):  - with alphabetic order  - with alphabetic lead authorship  - with alphabetic not lead authorship | 0.41%  0.32%  0.53% |
| Health | Pignatelli, 2005^77^ | Senior clinical researchers in France | Practice of ordering authorship:  - solely by main author  - after consultation with co-authors  - on request by co-author  - another way | 18%  18%  0  64% |
| Social sciences | Brown, 2006^81^ | Multiauthored articles from academic institutions published n marketing journals | Percent alphabetical ordering of authors:  - from 1991 to 2000  - among 19 journals (range)  - top journals vs. other (P<0.001)  - 2/3/≥4 authors (P<0.001) | 42.9% vs. 46.7%  32.3% – 61.4%  49.2% vs. 43. 1%  58.6%/27.6%/15.8% |
| Social sciences | Einaw, 2006^83^ | Faculty of economic or psychology departments, Econometric Society (ES) fellows, Nobel laureates and Clark Winners, authors of articles in economics journals in USA | Increase in probability for status with each letter closer to the front of the alphabet:  - to be tenured at top 5 economics departments  - to be tenured at top 5 psychology departments  - to be tenured at top 35 economics departments  - to be tenured at top 5 psychology departments  - to be tenured as ES fellow at top 10 economics departments  - to be tenured as Nobel/Clark winner at top 10 economics departments | 0.99%  0.26%  0.11%  0.05%  0.77%  0.18% |
|  |  |  | Percent multiauthored articles with alphabetical authorship in economics journals | 87.7% |
| Multidisciplinary | Laband, 2006^84^ | Articles in journals from medicine, natural sciences, economics, social sciences and general journals | Mean change in prevalence of alphabetical authorship in co-authored articles from 1974 to 1999:  - general journals (*Science*, *Nature*)  - medical journals  - natural sciences journals  - economics journals  - social sciences journals | 47.6% decrease  82.0% decrease  39.1% decrease  9.9% increase  18.6% increase |
| Social sciences | Manton, 2006^85^ | Business faculty in USA | Opinion on method of listing authors:  - most work done  - alphabetical order  - study conceptualization  - seniority  - other | 68.5%  11.6%  10.9%  0.7%  6.9% |
| Social sciences | Moore, 2006^87^ | Authors of articles in educational research journals | Preferred method of authorship order:^h^  - contribution amount  - idea origination  - mechanical decision (e.g., alphabetical order)  - assisting colleagues (e.g., tenure)  - seniority/leadership  - grant recipient  - from thesis/dissertation | 91.7%  36.7%  20.0%  16.7%  15.0%  10.0%  8.3% |
| Health | Baerlocher, 2007^89^ | Articles in general medical journals | Satisfaction of ICMJE criteria 1 and 2, depending on byline position:  - first  - second  - middle  - last | 92.5% – 100.0%  73.4% – 100.0%  56.2% – 98.3%  69.1% – 99.1% |
| Health | Kurichi, 2007^95^ | Chairs of surgery departments in USA medical schools | Likelihood for authorship position in regard to serving as chair:  - to be last vs. first vs. contributing author when chair (P<0.01)  - to be contributing author | 36.8% vs. 28.8% vs. 34.4%  P<0.01 |
| Social sciences | Manton, 2007^96^ | Faculty of colleges of business in USA | Preferred method of listing co-authors:  - most work – first author  - alphabetical listing  - conceptualizing research – first author  - most senior member – first author  - other reasons | 66.9%  16.0%  9.9%  1.1%  5.0% |
| Social sciences | van Praag, 2008^105^ | Articles published in mainstream economics journals | Prevalence of articles with alphabetical authorship | 88% |
|  |  |  | Chance that 1% lower-letter-ranked name increases total and annual publication output | 3.3% |
| Multidisciplinary | Hu, 2009^107^ | Articles in biomedical or multidisciplinary journals | Increase in prevalence of equal first authorship in one biomedical journal | ~8% in 1999 to ~18% in 2008 |
|  |  |  | Prevalence of equal first authorships in 2008 | ~14% – ~18% |
| Social sciences | Maciejeovsky, 2009^108^ | Faculty members and advanced graduate students from economics, marketing and psychology in USA/UK | Prevalence of alphabetical authorship in journals from:  - economics (n=5)  - psychology (n=6)  - marketing (n=4) | 87.5%  49.9%  33.0% |
|  |  |  | Preferences for credit to a position in multiauthored articles relative to single-authored articles (standardized regression coefficient, P<0.05):  - for psychologist to value single authored articles more than other disciplines  - for psychologists to differentiate more carefully between authorship positions | −0.38  –0.38 |
|  |  |  | Inference based on authorship order (standardized regression coefficient, P<0.05):  - for psychologist to favour candidate with more non-alphabetical authorships  - for economists to favour candidate with more alphabetical authorships | 0.60  −0.34 |
|  |  |  | Simulated contribution credit to author by evaluator from same or different discipline:  - author from economics/marketing/psychology – evaluator from economics/marketing/psychology  - author from marketing – evaluator from economics/marketing/psychology | 31.2/30.4/32.9  31.8/30.4/32.9 |
| Health | Akhabue, 2010^115^ | Original research articles from general medical journal | Increasing trend in equal authorships from 2000 to 2009  Prevalence of equal first authors (range)  Prevalence of equal 3 of more first authors (range) | from <1% to 1% – 8.6%  57% – 86%  3% – 14% |
| Social sciences | Chan, 2010^117^ | Multi-authored original research articles from academic real estate journals | No change in prevalence of alphabetical authorship from 1990 to 2006 | 61.7% – 65.3% |
|  |  |  | Likelihood for alphabetical authorship (estimated coefficient, P<0.01):  - more in higher quality articles  - less with increase in authors number  - more with higher academic ranking of authors  - more with European authors | 2.5779  −0.9701  0.1252  0.5828 |
| Multidisciplinary | Frandsen, 2010^118^ | Articles from economics, library information science (LIS) and high-energy physics (HEP) journals | Yearly change in share of articles with alphabetic authorship (P<0.05 or 0.01) from 1978 to 2007:  - 2 or 3 authors in economics journals  - 2-4 authors in LIS journals  - 2 or ≥5 authors in HEP journals | 3% and 7% increase  3% – 10% decrease  2% and 9% increase |
| Health | Walker, 2010^129^ | Corresponding authors of original research articles in medical journals | Opinion on position with greatest merit for promotion:  - first  - last  - corresponding  - other | 75.9%  6.7%  9.2%  8.2% |
|  |  |  | Prevalence of times when authorship order was made:  - prior to manuscript writing  - during manuscript writing  - after manuscript writing | 58%  28%  12% |
|  |  |  | Order changed after initial order decision was made | 12% |

*Abbreviations: CI, confidence interval; SD, standard deviation; ICMJE, International Committee of Medical Journal Editors.

^a^The same study as Zuckerman, 1967.^8^

^b^Partial or full replication or modification of questionnaire by Spiegel and Keith Spiegel, 1970.^11^

^c^P<0.0001 for all comparisons.

^d^Percentages are rounded to full numbers and do not add up to 100 because of contributions of non-authors.

^e^On a scale from 1 (lowest credit) to 10 (highest credit); 2 out of 12 respondents indicated that their institution had a policy on describing contributions in curricula vitae.

^f^Average percentage for additional authors (5^th^ to 10^th^).

^g^On a scale from 1(not appropriate or never) to 9 (entirely appropriate or often) for appropriateness or behaviour occurrence; and from 1 (no firsthand knowledge) to 4 (often observed). SD is not presented as they were reported with averages only for behaviour appropriateness.

^h^More than one choice was possible.
