# Supplementary material for: A Systematic Review of Research on the Meaning, Ethics and Practices of Authorship across Scholarly Disciplines
Source: PLoS One. 2011 Sep 8;6(9):e23477. doi: 10.1371/journal.pone.0023477 (PMC3169533; doi:10.1371/journal.pone.0023477)
Supplement: Table S5 — Results of studies addressing ethical authorship practices. (DOC) [file pone.0023477.s005.doc]

**Table S5.** Ethical and unethical authorship practices*

| **Research field** | **Reference** | | | **Study population** | **Outcome** | | | **Result (prevalence/percentage, number, score, P-value, odds ratio)** |
| --- | --- | --- | --- | --- | --- | --- | --- | --- |
| Social sciences | Spiegel, 1970^11^ | | | Psychologists in USA | Ethical not to give authorship but only footnote to colleague who failed to keep agreement on study work | | | 85% |
|  |  |  |  |  | Ethical to publish multiple publications from the same study, each indicating that it is part of large project | | | 90% |
| Health | Werley,^a^ 1981^13^ | | | Nursing professionals in USA | Ethical not to give authorship but only footnote to colleague who failed to keep agreement on study work | | | 88% |
|  |  |  |  |  | Ethical to publish multiple publications from the same study, each indicating that it is part of large project | | | 87% |
| Social sciences | von Glinow,1982^14^ | | | Professionals associated with management journals in USA | Unethical to add prestigious name without contribution: | | | 87% |
| Health | Waltz,^a^ 1985^16^ | | | Health professionals in nursing in USA | Ethical not to give authorship but only footnote to colleague who failed to keep agreement on study work | | | 89% |
|  |  |  |  |  | Ethical to publish multiple publications from the same study, each indicating that it is part of large project | | | 100% |
|  |  |  |  |  | Ethical to ask about collaboration before doing study after someone’s idea | | | 85% |
|  |  |  |  |  | Ethical to give footnote only to author who quits collaboration after disagreement and another author completes study | | | 82% |
| Health | Gay,^a^ 1987^17^ | | | Health professionals in nursing in USA | Ethical to give authorship for a research team even if one person does not make significant contribution to paper but does to general research effort | | | 81% |
|  |  |  |  |  | Ethical to publish multiple publications from the same study, each indicating that it is part of large project | | | 81% |
|  |  |  |  |  | Ethical not to give authorship but only footnote to colleague who failed to keep agreement on study work | | | 88% |
| Social sciences | van der Kloot, 1991^18^ | | | Social psychologists and psychometricians in The Netherlands | Professors and junior researchers disagree less often about authorship then university teachers and university lecturers | | | P=0.012 |
| Multidisciplinary | Eastwood, 1996^29^ | | | Postdoctoral fellows at a university in USA | Willingness to engage in giving undeserved authorship:  - ethics training vs. no ethics training (P<0.04)  - plans vs. no plans for future academic career | | | 31.8%  38.7% vs. 27.7%  34.0% vs. 28.0% |
| Health | Slone, 1996^30^ | | | First authors from USA on papers from a radiology journal | Reported undeserved authorship for co-authors:  - overall  - when 7-10 co-authors | | | 17%  30% |
|  |  |  |  |  | Reasons for undeserved authorship:  - sense of obligation or fear of offending  - only referring patients  - to gain favour, repay or reciprocate  - pressure or demand from co-author | | | 40%  29%  10%  12% |
|  |  |  |  |  | Decisions about authorship made during planning stages vs. later associated with fewer undeserved authors (P<0.001) | | | 47% vs. 23% |
| Health | Bhopal, 1997^31^ | | | Staff from university medical school in UK | Reported problems with authorship:  - any problem  - not included when authorship was deserved  - included when authorship was not deserved  - not aware of being author  - assigned inappropriate authorship  - incorrect order of authors | | | 64%  48%  38%  32%  30%  23% |
|  |  |  |  |  | Gift authorship:  - perceived as a problem  - very of fairly common problem  - should be banned  - statement on contribution as best practical or effective prevention strategy | | | 69%  66%  62%  56%, 40% |
| Social sciences | Hamilton, 1997^33^ | | | Business and non-business university faculty in USA | Adding non-contributing author is unethical^b^ | | | 85% - 97% |
|  |  |  |  |  | Willingness to add non-contributing author: business vs. non-business^b,c^ | | | 3% – 26% vs. 0% – 8% |
|  |  |  |  |  | Think a colleague would perceive adding non-contributing author as ethical^b,d^ | | | 6% – 18% vs. 0% – 6% |
|  |  |  |  |  | Colleague would add non-contributing author^b,e^ | | | 12% – 44% vs. 0% – 8% |
| Health | Bulter, 1998^36^ | | | Nurses expected to publish research in Canada | Agreement of modal responses among nurses of different professional status on:  1) unethical for someone of higher status to share authorship without substantial contribution  2) unethical for professor who only reads a paper without any change to be co-author  3) failing to follow through the work after planning study deserves only footnote | | | 80% |
| Health | Flanagin, 1998^38^ | | | Corresponding authors from USA on articles in large and small medical journals | Reported prevalence of research articles with undeserved or undisclosed authorship:  - honorary authors  - ghost authors | | | 16%  13% |
|  |  |  |  |  | Reported prevalence of research articles with ghost authors higher in large- (17%) than small- circulation (9%) journals | | | OR=0.49 (95% CI 0.27-0.88) |
| Health | White, 1998^41^ | | | First authors from USA on papers on nursing research | Reported issues, problems and concerns about author inclusion or ordering | | | 16%, 10% |
| Health | Wilcox, 1998^42^ | | | Cases brought to university ombuds office in USA | Authorship issues in cases 1991/92 vs. 1996/97 | | | 2.3% vs. 10.7% |
| Natural sciences | Tarnow, 1999^46^ | | | Postdoctoral fellows in physics in USA | Reported papers where supervisor should not have been author per APS guidelines | | | 14% |
|  |  |  |  |  | Reported papers where 1 or more authors other then supervisor or postdocs should not have been authors per APS guidelines | | | 33% |
|  |  |  |  |  | Reasons for inappropriate authorship (No. entries):  - relationship building  - minor contributions  - previous or expected contributions  - crediting staff in social sense (part of group) | | | 11  11  7  6 |
| Health | Price, 2000^50^ | | | Faculty from institutions granting graduate degrees in nursing in USA | Experiences and opinions on unethical authorship practices in doctoral granting vs. non-granting institutions:  - omitted as author although deserved  - listed as author without permission  - was offered guest authorship  - authored article with a guest author  - been a ghost author  - co-authored article with ghost author  - sometimes important figure should be guest author  - ghost authorship very common or common practice | | | 51% vs. 25%  15% vs. 5%  122% vs. 7%  49% vs. 25%  24%  7%  20%  48% vs. 25% |
|  |  |  |  |  | Experiences and opinions of highly published authors vs. other authors on unethical authorship practices:  - omitted as author although deserved  - authored article with a guest author  - been a ghost author | | | 56% vs. 30%  52% vs. 31%  34% vs. 18% |
| Health | Reidpath, 2001^53^ | | | Authors of articles published in general medical journal | Reported authorship was among stipulations for sharing data-set from their article | | | 4 out of 19 |
| Health | Mainous, 2002^56^ | | | Corresponding authors of research articles in medical journals | Personal or professional concerns enter authorship decision (mean±SD):^f^  - more often if first (P=0.04) or co- author (P=0.004) on more articles, or when articles were important for advancement (P=0.008) | | | 2.9±1.1 |
|  |  |  |  |  | Perceived importance of personal or professional factors for authorship decision when ICMJE criteria not met (score ≥2.0):^f^  - person part of team with only general relation to paper  - provides access to data or patient population  - has administrative power over respondent  - had background information or helped with proposal  - planned active role, but not followed  - provided financial, material or other support  - performed important work but not writing  - mentor to respondent | | | 2.2±1.1  2.9±1.3  2.1±1.3  2.4±1.0  2.0±1.1  2.1±1.2  3.3±1.1  2.0±1.2 |
|  |  |  |  |  | Opinion on effective ways for authorship decisions:^f^  - limiting number on byline  - stating contributions in article  - listing as contributor in acknowledgment | | | 2.7±1.2  3.2±1.3  1.7±0.9 |
| Health | Mowatt, 2002^57^ | | | Corresponding authors of Cochrane systematic reviews | Prevalence of honorary authors or ghost and honorary authors | | | 39%, 2% |
|  |  |  |  |  | Prevalence of ghost authors:  - editorial team member  - named individuals, lay reviewers, peer reviewers, authors of previous reviews | | | 9%  6%  3% |
| Natural sciences | Tarnow, 2002^58^ | | | Members of American Physical Society (APS) | Probability that additional author is inappropriate:  - APS guideline  - ICMJE guideline  - “direct contribution” | | | 23%  67%  59% |
|  |  |  |  |  | Comfort for younger vs. older respondent to deny undeserving authorship:  - comfortable  - not comfortable | | | 21% vs. 45%  39% vs. 20% |
| Health | Hwang, 2003^61^ | | | Research articles in medical journal | Prevalence of undeserved ICMJE authorship^g^ | | | 32.5% |
| Health | Bates, 2004^62^ | | | Research articles in medical journals with different contribution declaration forms | Prevalence of undeserved ICMJE authorship:^g^  - open ended declaration form  - list of coded contributions  - instructional form for qualifying contributions | | | 9.5%  21.5%  0.5% |
| Health | Buchkowsky, 2004^64^ | | | Clinical trials published in medical journals | Increase in author affiliation with industry from 1981/1984 to 1997/2000 | | | 8% – 66% |
| Health | Cohen, 2004^65^ | | | Members of US and Canadian Academy of Pathology (USCAP) | Probability that additional author is inappropriate:  - APS guideline  - ICMJE guideline  - “direct contribution”  - newly proposed guideline | | | 45%  65%  56%  59% |
|  |  |  |  |  | Reported denying undeserved authorship | | | 5% |
| Health | Marušić, 2004^67^ | | | Research articles in general medical journal | Prevalence of undeserved ICMJE authorship | | | 59.2% |
| Social sciences | Meyer, 2004^68^ | | | Editorial members of accounting journals and young accounting faculty members in USA | Perceived behaviour appropriateness/ behaviour occurrence/ actual knowledge of occurrence of co-authorship issues:^h^  - co-author starts project and does not follow, but maintains authorship  - professor lists fellow faculty as author without contribution  - colleagues add each other to own manuscripts without credit  - adding prestigious name to increase chances for acceptance  - professor insists on senior (first) authorship on work co-authored with junior colleague | | | 3.3/6.3/2.5  2.2/4.7/1.9  1.3/4.2/1.5  1.4/4.5/1.5  3.0/4.6/1.4 |
| Health | Procyshyn, 2004^69^ | | | Research articles on antipsychotic drugs in medical journals | Prevalence of authors affiliated with 3 pharmaceutical firms | | | 74.6%/23.3%/5.6% |
| Health | Szirony, 2004^70^ | | | Nursing faculty members in USA | Formal teaching to graduate students about authorship credit in publications | | | 44.3% |
|  |  |  |  |  | Unethical or questionable to deny authorship and grant only acknowledgement to a colleague who performed data analysis and help write results in manuscript | | | 30.9% and 35.6% |
| Social sciences | Apgar, 2005^71^ | | | Members of Society for Social Work and Research in USA | Ethical/undecided/unethical to:  - not give credit to colleagues who helped collect data | | | 2%/8%/90% |
| Health | Freda, 2005^73^ | | | Editors of nursing journals | Reported prevalence of ethical issues about authorship encountered in editorial work | | | 5% |
| Health | Joubert, 2005^75^ | | | Authors of research papers from university in South Africa | Reported prevalence of:  -papers where authors discussed authorship before publication  - papers where authors experienced no problems in authorship  - authors who satisfied ICMJE authorship criteria | | | 48%  64%  51.4% |
| Social sciences | Mixon Jr, 2005^76^ | | | Articles published in more and less prestigious economics journals | Ratio (mean±SD) between number of authors and contributors in acknowledgment (P=0.01):  more prestigious journals (n=2)  less prestigious journals (n=2) | | | 0.21±0.19, 0.19±0.19  0.38±0.30, 0.49±0.32 |
| Health | Pignatelli, 2005^77^ | | | Senior clinical researchers in France | Opinion on gift authorship:  - is questionable practice  - is frequent or very frequent  - must be condemned  - could be reduced by signed declaration from authors/limiting no. of articles needed for promotion/ sharing credit among authors/ contributorship system | | | 59%  77%  56%  69%/36%/25%/51 |
|  |  |  |  |  | Opinion on ghost authorship:  - is questionable practice  - is frequent or very frequent  - must be condemned  - have a proposal for banning this practice | | | 92%  40%  84%  36% |
|  |  |  |  |  | Reported experience:  - was granted authorship without knowledge  - omitted as author although deserving contribution  - received gift authorship  - participated in granting gift authorship  - wrong order of authors on byline | | | 62%  41%  59%  49%  41% |
| Health | Dhaliwal, 2006^82^ | | | Faculty in teaching hospital in India | Reported conflict over authorship total:  - for article where author (ownership of data/gift authorship/other issues)  - for article where not author (ownership of data/gift authorship/other issues) | | | 39%  27% (24%/43%62%)  25% (58%/37%/42%) |
| Social sciences | Manton, 2006^85^ | | | Business faculty in USA | Reported experience with co-authors:  - with very little contribution  - with no contribution | | | 35.6%  18.5% |
| Health | Marušić, 2006^86^ | | | Authors of articles in general medical journal | Prevalence of authors not satisfying ICMJE criteria according to contribution declaration form (P<0.001):  - open ended  - categorical  - instructional | | | 62.6%  54.7%  18.7% |
| Multidisciplinary | | Funk, 2007^90^ | | NIH postdoctoral fellows in USA | Ethically appropriate responses (mean±SD) to case vignettes at 3 time points after training on RCR:  - no. appropriate responses to 11 items (score 0-11)  - rate of appropriate responses for 3 individuals in case vignette (score 0-3) | | | 4.9±1.8/5.0±1.7/4.9±1.9  2.2±0.7/2.0±0.7/1.7±0.8 |
| Social sciences | | Geelhoed, 2007^91^ | | Authors of articles in clinical psychology journals | Experiences about fairness and ease of authorship decision process:  - completely or very satisfied with process  - unwarranted authorship (tenured vs. untenured faculty, P<0.05)  - deserved authorship not granted  - disagreement about authorship | | | 84%  14.7% (6.4% vs. 23.8%)  6.4%  4.6% |
| Health | | Gotsche, 2007^92^ | | Clinical trial protocols and publications from Sweden | Prevalence of ghost authorship | | | 75% (95% CI 60%-87%) |
| Health | | Hren, 2007^93^ | | Medical students with or without instruction on ICMJE criteria, physicians and medical faculty in Croatia | Students without instruction rate critical revision of manuscript and final approval of article lower than other groups^i^ | | | P<0.001 |
|  |  |  |  |  | Cluster analysis for all groups showed conception/design, analysis /interpretation, drafting manuscript as most important cluster; with final approval clustering close only for students with instructions^i^ | | |  |
| Social sciences | | Manton, 2007^96^ | | Faculty of colleges of business in USA | Reported that co-authors did very little/no work | | | 40.9%/89.0% |
| Health | | Peppercorn, 2007^97^ | | Articles on breast cancer clinical trials in medical journals | Prevalence of pharmaceutical company authorship on published studies | | | 26% |
| Health | | Tungaraza, 2007^99^ | | Published clinical trials on psychiatric drug treatment | Prevalence of industry-authored studies | | | 40% |
| Health | | O’Brien, 2009^109^ | | Corresponding authors of original research articles in general medical journals | | Reported experience or opinion:  - asked to give authorship for provision of data for which respondent was charged  - asked to give authorship for obtaining data they had already collected  - been on article with honorary coauthor  - presence of renowned clinician name increase importance of article to respondent | | 18.4%  16.0%  51.6%  60.3% |
|  |  |  |  |  |  | Honorary authorship has potential adverse effects:  - for honorary author^j^  - for coauthors | | 73.4%  83.2% |
|  |  |  |  |  |  | Honorary authorship and patient care:  - can affect patient care  - was personally involved when honorary authorship affected patients | | 52.5%  2.4% |
| Multidisciplinary | | Wager, 2009^113^ | | Editors of journals published by Blackwell | | Reported experience in their journals  - host/gift authorship never occurred  - authorship disputes never occurred | | 30% /30%  28% |
|  |  |  |  |  |  | Perceived severity/confidence/frequency of ethical issues in their journals (mean rating):^k^  - gift authorship  - disputed authorship  - ghost authorship | | 0.67/0.51/1.08  0.58/9.90/0.81  0.37/0.61/0.48 |
|  |  |  |  |  |  | Perceptions (mean rating)^j^ of editors with >5 or ≤5 years experience about trends in ghost authorship | | 3.00 vs. 3.65 |
| Health | Ahmed, 2010^114^ | | Participants in bioethics course in Bangladesh | | | | Experiences of authorship conflict:  - aware of conflicts in own or other institutions  - had conflict with co-authors^l^ | 71%  31% |
| Health | Lacasse, 2010^120^ | | Public policies of academic medical centres in USA | | | | Prevalence of policies explicitly banning ghostwriting  Prevalence of policies banning ghostwriting in practice | 20%  6% |
| Health | Nastasee, 2010^123^ | | Articles in medical journals | | | | Increase in acknowledgment of medical writing from 2000 to 2007 | 5.1% – 11.3% |
| Health | Rose, 2010^125^ | | Clinical trials published in oncology journal | | | | Odds (95% CI) for authors reporting financial ties to industry:  - industry vs. non-industry sponsored studies  - authors satisfying ICMJE criteria  - sponsor employees excluded | 5.0 (3.0-6.0) vs. 2.5 (1.3-4.8)  3.6 (2.6-5.0)  3.6 (2.5-5.1) |
| Natural sciences | Seeman,^m^ 2010^127^ | | Faculty from departments of chemistry in USA | | | | Experience of not getting authorship or acknowledgment when thought it was deserved:  - by professor or teacher  - colleague from own/other institution | 50%  35%  41%/42% |
|  |  |  |  |  |  |  | Experience of discovering to be author on paper:  - after its submission but publication  - after its acceptance but publication  - after it was in print  - all of above | 20%  15%  25%  5.3% |

*Abbreviations: CI, confidence interval; SD, standard deviation, OR, odds ratio; NIH, National Institutes of Health, USA; RCR, responsible conduct of research.

^a^Partial or full replication or modification of questionnaire by Spiegel and Keith Spiegel, 1970.^11^

^b^Request for authorship from either department head/mentor, department head/accreditation, colleague/tenure, colleague/promotion, colleague/raise, student/job.

^c^Significant differences for requests by department head for unit accreditation and colleague for raise.

^d^Significant difference for requests by department head for unit accreditation.

^e^Significant differences for all requests.

^f^On a scale from 1 (never) to 5 (always) or 1 (not effective) to 5 (very effective). For perceived importance of personal of professional factors in authorship decision when ICMJE criteria were not met, respondents for whom publications were important rated higher administrative power (P=0.009) and collaboration in the past (P=0.04) as factors; junior faculty had higher ratings for administrative power (P=0.001), planned active role but not followed (P=0.03), mentor (P=0.001), and potential collaboration in future (P=0.02).

^g^Fulfilment of the 3^rd^ ICMJE criterion (approval of the version to be published) was considered satisfied for all articles.

^h^On a scale from 1 (not appropriate or never) to 9 (entirely appropriate or often) for appropriateness or behaviour occurrence; and from 1 (no firsthand knowledge) to 4 (often observed). SD is not presented as they were reported with averages only for behaviour appropriateness.

^i^Data presented as figures and cluster diagrams, P values reported.

^j^Includes exposure to misconduct or fraud allegations (29.1%), undermines credibility (24.4%), resentment from colleagues (17.4%) for honorary author, and dilution of relative contributions (54.1%) and resentment to honorary author (10.2) for co-authors.

^k^On a scale from 0 (not a problem, not at all confident, never) to 3 (very serious problem, highly confident, very often); for trend the scale was from 1 (decreasing a lot) over 3 (increasing slightly) to 5 (increasing a lot).

^l^Most common types of conflict: order of authorship violated, genuine authorship deprived, compelled to give authorship without contribution, authorship promised but not given later.

^m^The same study as House and Seeman^119^ and Seeman and House.^126^
