# Supplementary material for: A Systematic Review of Research on the Meaning, Ethics and Practices of Authorship across Scholarly Disciplines
Source: PLoS One. 2011 Sep 8;6(9):e23477. doi: 10.1371/journal.pone.0023477 (PMC3169533; doi:10.1371/journal.pone.0023477)
Supplement: Table S6 — Results of studies addressing authorship in researcher – student/non-researcher collaborations. (DOC) [file pone.0023477.s006.doc]

**Table S6.** Authorship in researcher – student/non-researcher collaborations*

| **Research field** | **Reference** | **Study population** | **Outcome** | **Result (prevalence, score, P-value, odds)** |
| --- | --- | --- | --- | --- |
| Social sciences | Spiegel, 970^11^ | Psychologists in USA | Opinion on deserved authorship for:  - paid consultant who makes substantial contributions  - qualified sub-doctoral assistant who makes substantial contributions  - student assistant who collected and statistically analyzed data  - volunteer assistant who helped with the study  - service chief, program administrator, head of the lab only | 45%  91%  19%  4%  14%, 18%, 11% |
|  |  |  | Preferred outcome for student-professor collaboration (both designed the study, student collected and analyzed data and wrote article):  - first authorship for student  - first authorship for professor  - random decision | 58%  32%  4% |
| Health | Werley,^a^ 1981^13^ | Nursing professionals in USA | Opinion on deserved authorship for:  - paid consultant who makes substantial contributions  - qualified subdoctoral assistant who makes substantial contributions  - student assistant who collected and statistically analyzed data  - volunteer assistant who helped with the study  - service chief, program administrator | 28%  78%  14%  3%  15%, 12% |
|  |  |  | Preferred outcome for student-professor collaboration (both designed the study, student collected and analyzed data and wrote article):  - first authorship for student  - acknowledgment for professor  - first authorship for professor | 52%  30%  14% |
|  |  |  | Opinion on deserved authorship:  - researchers vs. others for superficial supervision of doctoral thesis  - doctoral students vs. others on no recognition for volunteer research team member  - doctoral students vs others on professor’s authorship after reading article and making no comments or few editorial revisions | 19% vs. 10%  0% vs. 4%  11% vs. 6%, 20% vs. 12% |
| Health | Waltz,^a^ 1985^16^ | Health professionals in nursing in USA | Opinion on deserved authorship for :  - paid research assistant who makes substantial contributions^b^  - paid or volunteer subdoctoral personnel if they make same contributions as doctoral fellows^b^  - having a lab where student works but not contribution  - professor asked by student to be author without contribution | 85%  85%  15%  4% |
| Health | Gay,^a^ 1987^17^ | Educators in nursing in USA | In faculty-doctoral student collaboration, authorship should be decided before study | 78% |
|  |  |  | Volunteers who make similar contributions as others deserve authorship | 85% |
|  |  |  | Footnote for volunteer graduate student who helps with statistical analysis | 77% |
|  |  |  | No authorship for faculty who provide minimal consultation to student work | 50% |
|  |  |  | Faculty who makes little or no contribution to student research should not share authorship | 80% |
|  |  |  | Faculty who serve as academic advisors to student project should be acknowledged in footnote | 55% |
|  |  |  | Publication from dissertation is an independent research effort of student | 67% |
|  |  |  | When student and faculty collaborate, student is the first author on manuscript | 65% |
|  |  |  | When student updates literature review, analyzes data and writes manuscript of a faculty-designed study, coauthorship should be alphabetical | 82% |
|  |  |  | When students cannot fulfil research commitment, faculty receives first authorship even if prior agreement was that students are first authors | 56% |
| Social sciences | Costa, 1992^19^ | Psychology students and faculty in USA | Faculty vs. students views of authorship order for published dissertation with different level of faculty input:^c^  High faculty input – student first author, faculty second  High faculty input – faculty first author, student second  Low faculty input – student first author, faculty second  Low faculty input – faculty first author, student second | 65.2% vs. 46.3%  17.4% vs. 46.3%  20.0% vs. 42.0%  0 vs. 3.2% |
| Social sciences | Goodyear, 1992^20^ | Editorial board members and authors of psychology journals in USA | Reported critical incidents related to student research:^d^  1) taking other’s ideas or manuscripts  2) failure to give warranted credit  3) giving unwarranted credit |  |
| Multidisciplinary | Brown-Wright, 1997^32^ | Graduate assistants and faculty members in USA | Assistance in analysis of research data warrants authorship for graduate assistant – faculty vs. assistants | 88% vs. 96% |
| Multidisciplinary | Rose, 1988^40^ | Graduate students in physics, biological, engineering and social sciences in USA | Opinion of men vs. women that professor as first author on manuscript if from dissertation (mean±SE):^e^ | 1.8±0.2 vs. 1.6±0.2 |
|  |  |  | Professor submitting manuscript without discussing authorship with student (mean±SE): ^e^  - unethical in any case (men vs. women)  - unethical if dissertation  - unethical if student did work | 2.2±0.1vs. 1.8±0.1  1.9±0.1  1.5±0.1 |
|  |  |  | Perceived reporting of authorship problems (mean±SD):^f^  - likelihood to talk to the dean, file complaint, contact journal  - effectiveness of talking to the dean, filing complaint, contacting journal  - consequences of talking to the dean, filing complaint, contacting journal | 2.7±1.6, 2.1±1.4, 1.6±1.1  3.9±1.8, 3.75±1.9, 3.1±2.0  5.4±1.6, 5.7±1.7, 6.0±1.7 |
| Social science | Louw, 1999^44^ | Academic and non-academic psychologists and masters’ degree students in South Africa | Choice of first authorship by academics/ non-academics/ and students:  - supervisor first author when supervisor initiates project, both student and supervisor active  - student first author when student initiates project, both student and supervisor active  - supervisor first author when both jointly initiate project, both are active, student writes first draft but loses interest after several manuscript submissions  - supervisor first author when both active, student writes dissertation, supervisor writes manuscript and adds literature | 75%/ 73%/ 84%  82%/87%/92%  79%/87%/90%  75%/80%/79% |
| Social science | Bartle, 2000^48^ | Faculty and students from psychology departments in USA | Agreement of faculty vs. students with student-faculty collaboration:^g^  - students are sufficiently expert to warrant 1st authorship  - faculty and student status should not influence authorship  -student should be 1^st^ author from master thesis | 5±1 vs. 4±2  6±1 vs. 5±2  5±2 vs. 4±2 |
| Social sciences | Meyer, 2004^68^ | Editorial members of accounting journals and young accounting faculty members in USA | Perceived behaviour appropriateness/ behaviour occurrence/ actual knowledge of occurrence of co-authorship issues:^h^  - professors uses graduate students to do literature review, data collection and analysis but does not acknowledge  - professor agrees to chair dissertation only if joint authorship  - professor uses parts of graduate student work for his paper without acknowledgment  - major professor appears as senior (first) author on a manuscript from dissertation | 2.5/5.0/2.0  3.2/4.5/1.7  1.6/4.8/1.7  2.7/4.5/1.6 |
| Health | Szirony, 2004^70^ | Nursing faculty members in USA | Opinion that it is unethical vs. questionable practice of thesis advisor when student leaves and no communication possible regarding manuscript:  - to write and submit article with advisor first and student second author  -to write and submit article with student first and advisor second author  - to write article with help of department chair and submit it with student, advisor and chair as authors | 72.4% vs. 21.6%  61.8% vs. 30.9%  62.9% vs.26.5% |
|  |  |  | Opinion that it is unethical vs. questionable to deny authorship and grant only acknowledgement to a paid graduate student who completes data analysis and help write results and creates some tables in manuscript | 21.4% vs. 27.6% |
| Social sciences | Apgar, 2005^71^ | Members of Society for Social Work and Research in USA | Ethical/undecided/unethical to:  - co-author article with current student  - independently write article or develop study using student’s idea  - assume first authorship on article written by student | 91%/7%/2%  8%/31%/61%  5%/10%/85% |
|  |  |  | Attitudinal differences:^i^  - women less likely to think that using student’s idea was ethical  - teachers less likely then researchers to be undecided about being first author on paper written by student | odds=0.27  odds=0.18 |
| Social sciences | Sandler, 2005^78^ | APA members and students with a publication from student-faculty collaboration in USA | Involvement in a perceived unethical or unfair authorship assignment:  - total  - men vs. women (P=0.02)  - nontenured vs. tenured (P<0.001)  - number (mean±SD) of perceived incidents: men. vs. women (P<0.001) | 27.3%  37.0% vs. 63.0%  40.7% vs. 59.3%  21.5±19.8 vs. 13.6±12.9 |
|  |  |  | Why unfair (>10% positive response):  - someone/student/faculty received too much or some credit  - someone/student received too much or some credit | 25.9%/23.4%/17.9%  13.9%/13.9% |
|  |  |  | Why not reported (> 10% positive response):  - fear of negative consequences  - respondent instigated event  - incident did not reach level of importance | 25.4%  11.4%  10.9% |
| Natural sciences | Weltzin, 2006^88^ | Participants of ecology meeting in USA | Opinion on first authorship for:  - student who collaborates in all aspects of research with a professor and writes the manuscript  - researcher who develops the idea, receives grant, and technician collects data and supervises student who assist with data collections  - professor/student who collaborate together in ideas, research and writing, and professor does final revision and submits manuscript | 84%  78%  46%/46% |
| Social sciences | Geelhoed, 2007^91^ | Authors of articles in clinical psychology journals | Opinion of students vs. faculty on influences on authorship decision making (P<0.05):  - differences in power influence authorship  - perceived less power than other authors (mean±SD)^j^ | 38.5% vs. 14.1%  2.5±1.4 vs. 3.3±1.2 |
| Social sciences | Tryon, 2007^98^ | Doctoral students in school psychology in USA | Different opinions (mean score±SD, scale range 1-6) on:  - developing dissertation idea  desirable to be student vs. advisor  ethical to be student vs. advisor  - to be first or sole author  desirable to be student vs. advisor  desirable to be student vs. advisor | 5.2±1.4 vs. 4.7±1.1  5.6±1.1 vs. 4.9±1.3  4.9±1.8 vs. 2.1±1.5  4.3±1.8 vs. 1.9±1.4 |
| Natural sciences | Picard, 2010^124^ | Students and supervisors from agriculture school in Australia | No. agreeing with authorship statements – students (n=19) vs. professors (n=18):  - if you work for an article, you can expect authorship  - if students plans publication, supervisor would expect co-authorship  - on co-authored paper from thesis, supervisor decides on first author  - assistance with writing and editing merits authorship  - supervisor should help student write thesis or papers | 14 vs. 12  3 vs. 5  3 vs. 3  3 vs. 3  4 of 11 vs. 11 of 17 |
| Social sciences | Welfare, 2010^130^ | Students and faculty from US universities with graduate studies in education | Opinion of students vs. faculty for common practices (P<0.05):  - professor sole author when paid student helps collect and enter data  - professor sole author when paid student helps collect, enter and analyze data, writes literature review  - professor sole author when paid student conduct and transcribes interviews, code data  - professor sole author when student has idea, work together on all other parts of research and writing  - student sole author when student does project as class assignment and professor helps with editing  - student sole author when completes thesis and professor gives guidance  - professor sole author when paid student finds literature and write overview and professor revises overview and writes the rest | 66.4% vs. 56.8%  11.1% vs. 4.0%  49.6% vs. 64.6%  2.9% vs. 0.8%  60.5% vs. 57.0%  53.7% vs. 34.5%  40.5% vs. 42.8% |
|  |  |  | Opinion of students vs. faculty for recommended practices (P<0.05):  - processor sole author when paid student helps collect, enter and analyze data and professor writes manuscript  - professor sole author when paid student helps collect, enter and analyze data, writes literature review  - student sole author when student has idea, work together on all other parts of research and writing  - student sole author when completes thesis and professor gives guidance  - professor sole author when paid student finds literature and write overview and professor revises overview and writes the rest | 8.8% vs. 12.7%  1.8% vs. 0.8%  5.6% vs. 2.6%  59.6% vs. 49.2%  13.8% vs. 24.5% |
|  |  |  | For all scenarios, recommended authorship was greater than perceived practice | P<0.01 |
|  |  |  | Students’ opinion of importance higher than professors’ for:  - collecting qualitative data  - entering data into statistical programme  - analyzing the data  - writing literature review/introduction section  - writing methods section  - total time spent on project | P≤0.05 for all contributions |

*Abbreviations: CI, confidence interval; SD, standard deviation; SE, standard error; APA, American Psychological Association.

^a^Partial or full replication or modification of questionnaire by Spiegel and Keith Spiegel, 1970.^11^

^b^Agreement with other health professionals from the study (dentistry, medicine, pharmacy, social work).

^c^Faculty with higher academic rank or more teaching time gave students more credit (r=0.16, P<0.05, r=0.20, P<0.01, respectively); faculty assigned more credit to students that students did and students assigned more credit to the advisor then the faculty (F(1,418)=25.96, P<0.001; 3-way ANOVA).

^d^No numerical results presented.

^e^On a scale from 1 (highly unethical) to 7 (highly ethical).

^f^On a scale from least (1) to most (7) likelihood, effectiveness and possibility of negative consequences.

^g^On a scale from 1 (strongly disagree) to 7 (strongly agree); only statistically significant results presented.

^h^On a scale from 1 (not appropriate or never) to 9 (entirely appropriate or often) for appropriateness or behaviour occurrence; and from 1 (no firsthand knowledge) to 4 (often observed). SD is not presented as they were reported with averages only for behaviour appropriateness.

^i^Multinomial logit analysis; P<0.05 for gender and P<0.0 for teaching vs. research.

^j^Scale range of the Likert scale not reported.
